# Genomic variation and biogeography of Antarctic haloarchaea

**DOI:** 10.1186/s40168-018-0495-3

**Published:** 2018-06-20

**Authors:** Bernhard Tschitschko, Susanne Erdmann, Matthew Z. DeMaere, Simon Roux, Pratibha Panwar, Michelle A. Allen, Timothy J. Williams, Sarah Brazendale, Alyce M. Hancock, Emiley A. Eloe-Fadrosh, Ricardo Cavicchioli

**Affiliations:** 1 School of Biotechnology and Biomolecular Sciences, UNSW Sydney, Sydney, New South Wales 2052 Australia; 2 i3 Institute, University of Technology Sydney, Sydney, New South Wales Australia; 3 Department of Energy Joint Genome Institute, Walnut Creek, CA USA; 4 Present Address: Climate Change Cluster, Department of Environmental Sciences, University of Technology Sydney, Sydney, New South Wales Australia; 5 Present Address: 476 Lancaster Rd, Pegarah, Australia; 6 Present Address: University of Tasmania Institute of Marine and Antarctic Studies, Antarctic Gateway Partnership and Antarctic Climate and Ecosystem Research Centre, Battery Point, Tasmania Australia

**Keywords:** Haloarchaea, Halobacteria, Antarctica, Genome variation, Metagenomics, Pan-genome, Genomic islands, Replicons, Virus infection, Biogeography

## Abstract

**Background:**

The genomes of halophilic archaea (haloarchaea) often comprise multiple replicons. Genomic variation in haloarchaea has been linked to viral infection pressure and, in the case of Antarctic communities, can be caused by intergenera gene exchange. To expand understanding of genome variation and biogeography of Antarctic haloarchaea, here we assessed genomic variation between two strains of *Halorubrum lacusprofundi* that were isolated from Antarctic hypersaline lakes from different regions (Vestfold Hills and Rauer Islands). To assess variation in haloarchaeal populations, including the presence of genomic islands, metagenomes from six hypersaline Antarctic lakes were characterised.

**Results:**

The sequence of the largest replicon of each *Hrr*. *lacusprofundi* strain (primary replicon) was highly conserved, while each of the strains’ two smaller replicons (secondary replicons) were highly variable. Intergenera gene exchange was identified, including the sharing of a type I-B CRISPR system. Evaluation of infectivity of an Antarctic halovirus provided experimental evidence for the differential susceptibility of the strains, bolstering inferences that strain variation is important for modulating interactions with viruses. A relationship was found between genomic structuring and the location of variation within replicons and genomic islands, demonstrating that the way in which haloarchaea accommodate genomic variability relates to replicon structuring. Metagenome read and contig mapping and clustering and scaling analyses demonstrated biogeographical patterning of variation consistent with environment and distance effects. The metagenome data also demonstrated that specific haloarchaeal species dominated the hypersaline systems indicating they are endemic to Antarctica.

**Conclusion:**

The study describes how genomic variation manifests in Antarctic-lake haloarchaeal communities and provides the basis for future assessments of Antarctic regional and global biogeography of haloarchaea.

**Electronic supplementary material:**

The online version of this article (10.1186/s40168-018-0495-3) contains supplementary material, which is available to authorized users.

## Background

Sequencing new strains of a microbial species often uncovers genes not previously characterised as belonging to that species. The total pool of genetic material comprised by all members of a species is referred to as the ‘pan-genome’ [1]. It consists of the core genome that is common to all members of a species, plus all the flexible genome content that is present in some members of the species. By accumulating metagenome data for abundant environmental species, pan-genomes are beginning to be defined—for example, the genome of the marine *Prochlorococcus* sp. contains about 2000 genes (half core and half flexible), yet more than 13,000 genes of the species have been identified, with the pan-genome estimated to be on the order of 85,000 genes [2].

The flexible genome content can be contained in ‘genomic islands’ that are thought to derive from horizontal gene transfer events and can be identified as regions with low coverage when metagenome reads are mapped onto individual genomes [3]. As genomic islands can encode cell surface genes and variation in cell surface genes can arise in response to viral infection, genomic islands can be important vehicles for modulating virus-host interactions [2–4].

**Fig. 1:**
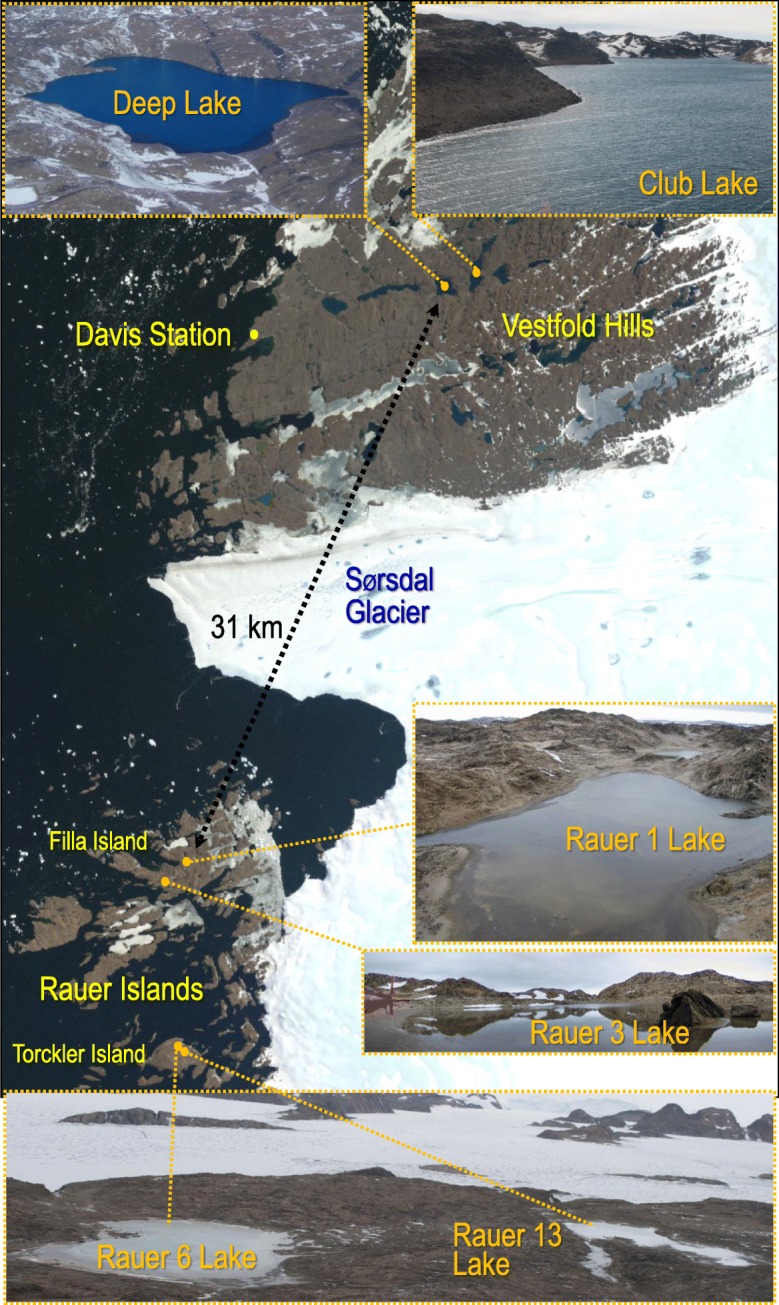
Hypersaline lakes in the Vestfold Hills and Rauer Islands sampled for metagenomics. Photo credits: Alyce Hancock (Rauer 1 Lake, Rauer 3 Lake); Sarah Payne (Rauer 6 Lake, Rauer 13 Lake, Club Lake); Rick Cavicchioli (Deep Lake); Landsat Image Mosaic of Antarctica (LIMA) project for the satellite image. *Hrr*. *lacusprofundi* ACAM34 isolated from Deep Lake [16] and R1S1 from Rauer 1 Lake [24]

Population-level genomic variation, genomic islands, and cell surface variation that mediates defence against viral infection have been described for a number of global hypersaline environments that support the growth of archaea and bacteria [5–13]. Studies have been performed on warm hypersaline systems from Chile, Spain and Australia [8–13] and a cold lake (Deep Lake) from the Vestfold Hills region of Antarctica [5–7]. Deep Lake is located ~ 9 km ENE from Davis Station (Fig. 1). The lake is perennially cold and water temperatures can drop to − 20 °C, although surface water temperatures can rise to around 10 °C for short periods in summer [5, 14–17]. Despite the low water temperatures, lake water does not freeze due to the high salinity, which is approximately 10 times marine salinity [17]. The haloarchaea that dominate Deep Lake in Antarctica (*Halohasta litchfieldiae*, DL31 and *Halorubrum lacusprofundi*) are different to the predominant species from warm environments (*Haloarcula*, *Haloferax volcanii*, *Haloquadratum walsbyi* and *Halobacterium salinarum*) [5], but it is not clear what factors control this distribution (e.g. environment, distance) and to what extent haloarchaeal genetic elements are shared globally.

Genome sequences are available for isolates of four distinct genera from Deep Lake: *Hht*. *litchfieldiae* which represents ~ 44% of the community, DL31 (unknown genus closely related to *Halolamina*) which represents ~ 18%, *Hrr*. *lacusprofundi* which represents ~ 10% and DL1 (*Halobacterium* sp.) which represents a minor fraction (~ 0.3%) [5]. Haloarchaeal genomes often comprise multiple replicons (chromosomes, megaplasmids, plasmids/viruses) [18]. The Antarctic haloarchaea contain multiple replicons that vary in size from 29 kb to 2.9 Mb, except for *Hht*. *litchfieldiae* strain tADL which has a single 3.3-Mb replicon [5].

A characteristic of these Antarctic haloarchaea is the sharing of long (> 5 kb), high-identity (~ 100% nucleotide identity) regions (HIRs) of DNA [5]. Because the lake supports ‘promiscuous’ intergenera gene exchange, it is difficult to define the pan-genome for the individual species. Yet, understanding this is important for evaluating the factors that control and mediate exchange and for establishing whether biogeographic boundaries exist for the global haloarchaea gene pool; the latter is particularly important for genetically characterising the Antarctic communities and assessing the likelihood of foreign species invading [14].

The microbial community of Deep Lake has been well studied [14] compared to Club Lake which is a large hypersaline system that neighbours Deep Lake (Fig. 1). A limited characterisation of microorganisms has been reported for a number of shallow hypersaline lakes within the Rauer Islands which are located ~ 30 km away from Deep Lake (Fig. 1) [19–23]. All these Antarctic hypersaline lakes are marine-derived [15, 19]. However, the concentration of major ions (Na, K, Ca, Mg, Cl) in the lakes is not identical. For example, Rauer 1 Lake and Deep Lake contain similar concentrations of Na (~ 70 g L^−1^), but Deep Lake has a tenfold higher concentration of Ca (2 g L^−1^) [17, 19]. The lakes provide unique opportunities for learning how microbial communities have evolved from marine to hypersaline conditions in this part of Antarctica.

*Hrr*. *lacusprofundi* strain ACAM34 was first isolated from Deep Lake around 30 years ago [16] and represents the first haloarchaeal species isolated from a cold environment [14]. *Hrr*. *lacusprofundi* strain R1S1 was recently cultivated from a laboratory enrichment of water sampled from Rauer 1 Lake [24]. In order to expand our understanding of strain-specific genome variation, in this study, we defined genomic traits of *Hrr*. *lacusprofundi* strain R1S1 from Rauer 1 Lake vs the type strain ACAM34 from Deep Lake and experimentally tested the susceptibility of each strain to infection by a newly isolated virus DLHTHV (Deep Lake head-tailed halovirus). DLHTHV was isolated from a summer 2014 Deep Lake water sample from a new strain of *Hrr*. *lacusprofundi* (DLSEC4) which lysed when grown in liquid medium (Erdmann and Cavicchioli, unpublished results). The virus was thereafter propagated using strain ACAM34. To gain knowledge of population-level genomic variation and assess endemism, we analysed metagenomes from samples collected from four hypersaline Rauer Island lakes, Club Lake and a Deep Lake time series (2006, 2008, 2013–2014) and used the data to characterise biogeographic patterns of genome evolution.

## Methods

### Genome sequencing and genome analyses

DNA was isolated from *Hrr*. *lacusprofundi* strain R1S1 as previously described [24] and sequenced using paired-end MiSeq Illumina technology at the Ramaciotti Centre for Genomics (UNSW Sydney, Australia), producing a total of 718,229 read-pairs with a bulk read length of 250 nucleotides. Genome assembly was performed using SPAdes [25]. Using the Mauve Contig Mover [26], 27 of the assembled R1S1 contigs, totalling ~ 2.7 Mb, were mapped onto the *Hrr*. *lacusprofundi* ACAM34 primary replicon (note: *Halorubrum lacusprofundi* ACAM34 = *Halorubrum lacusprofundi* ATCC 49239). Gaps between contigs were manually closed using PCR and Sanger sequencing, resulting in the closure of all but one gap. For the remaining gap, sequencing of a ~ 900 bp PCR product generated using the primers 5′-CGCTCATCGGAGTGTAG and 5′-GTGGGAACGGATGGAAC resulted in sequence termination (from either end) after ~ 410 nucleotides, leaving ~ 80 bp unsequenced. At the equivalent position in the ACAM34 primary replicon is an 80 bp region (between Hlac_2468 and Hlac_2469) with high GC content (76%) and a number of single nucleotide repeats that may obstruct DNA polymerase when performing sequencing reactions. The 2.7-Mb contig for the primary replicon plus 46 other contigs collectively represent the R1S1 genome and are available on Integrated Microbial Genomes (IMG) [27] with taxon ID 2671180119. Average nucleotide identity (ANI) was calculated using the ANI calculator [28]; genome synteny plots were created using NUCmer [29] with the minimum cluster length (-c flag) set to 500; The Artemis Comparison Tool (ACT) [30] was used for manually analysing variation between R1S1 and ACAM34 genomes using ACT files created on IMG [27]; CONTIGuator [31] was used for mapping R1S1 secondary replicon contigs onto the ACAM34 secondary replicons to distinguish between shared and unique secondary replicon content and for generating secondary replicon ACT files; multiple sequence alignments were created using Clustal Omega [32]; archaeal Clusters of Orthologous Genes (arCOGs) [33] were assigned using COGnitor in the COG software package [34]; R1S1 CRISPR sequences were identified with CRISPRfinder [35] and CRISPR spacer targets were identified as described previously [6]; mapping of sequencing reads onto R1S1 contigs was performed using Bowtie version 2.3.2 [36]; R1S1-specific HIRs were identified with BLASTN (standalone BLAST+ 2.2.30) [37] by using the secondary replicon sequences that were unique to R1S1 (i.e. absent in ACAM34) and finding matches (sequence identity ≥ 99%) in the genomes of *Hht*. *litchfieldiae* tADL, DL31 and DL1. Genome sequences for *Hht*. *litchfieldiae* strain tADL (single replicon), DL31 (primary and two secondary replicons), *Hrr*. *lacusprofundi* strain ACAM34 (primary and two secondary replicons) and DL1 (primary and one secondary replicon) were previously described [5] and accessed through IMG.

### Metagenome sequencing and analysis

Descriptions are provided (with photographs) for the 2013–2015 lake sampling expedition (Fig. 1; Additional file 1: Supplementary results), all the samples used for metagenomics (Additional file 2: Table S1) and the 33 metagenomes analysed (Additional file 2: Table S2). Biomass was collected by filtering water by sequential filtration through a 20-μm prefilter onto 3.0-, 0.8- and 0.1-μm filters, as described previously [5, 38]. The biomass of the flow through from the 0.1-μm filter from Deep Lake (summer 2006 and 2014) and Club Lake (summer 2014) samples was concentrated by tangential flow filtration using a Pellicon 2 Filter fitted with Biomax 50 (50 kDa) polyethersulphone membranes (Millipore, Sydney, NSW, Australia). DNA was extracted from biomass as described previously [5, 38]. DNA was sheared to 300 bp using the Covaris LE220 and size-selected using SPRI beads (Beckman Coulter). The fragments were treated with end-repair, A-tailing and ligation of Illumina-compatible adapters (IDT, Inc.) using the KAPA-Illumina library creation kit (KAPA Biosystems). qPCR was used to determine the concentration of the libraries prior to sequencing on the Illumina HiSeq-2500 to yield 150 bp paired-end reads at the DOE Joint Genome Institute. Quality-filtered metagenomic sequences for each sample were assembled with Megahit (version 1.0.6) [39], and all contigs > 200 bp were uploaded and annotated by the IMG pipeline [40]. The nucleotide sequences of seven loci from the primary replicons of R1S1 and ACAM34 (7 loci × 2 alleles = 14 query sequences) were used as query sequences in BLASTN searches (standalone BLAST+ 2.2.30) [38] to interrogate the Antarctic metagenomes. The query sequences contained the nucleotide sequences of the genes at each loci plus ~ 500 nucleotides upstream and downstream of the loci. Only matches with sequence identity ≥ 99% were accepted in order to minimise false-positive identifications. These analyses were performed to assess the representation of loci that were unique to each strain (e.g. *Hrr*. *lacusprofundi* ACAM34 provirus Hlac-Pro1), thereby assessing the representation of these strain markers within the *Hrr*. *lacusprofundi* populations of each lake. For metagenome read mapping of sampling sites that contained haloarchaea (i.e. not Rauer 1 Lake), reads from size fractions were combined to provide nine metagenome pools representing Rauer 3, 6 and 13 lakes; Club Lake; and Deep Lake summer 2006, 2008, 2013 and 2014 and winter 2014. Read mapping was performed using the BWA-MEM algorithm [41], with the resulting mapping files converted from SAM into BAM format, sorted (in the process removing soft- and hard-clipped reads) and indexed using Samtools [42]; coverage depth per nucleotide was obtained using the Samtools depth option. Per base position depth of coverage was binned (primary replicons, 5 kb; secondary replicons, 1 kb) and the median value used to infer abundance of the genomic region within the metagenome. From the binned median values of each of the nine metagenomes, a correlation matrix was produced and hierarchically clustered (scipy v0.19.1) [43] and the resulting dendrograms were subjected to optimal leaf ordering (polo v0.5) [44]. The distribution of coverage for each primary replicon within each metagenome was estimated by histogram binning, where coverage was assumed to be normally distributed. As these distribution estimates possessed a right-sided tail, the largest bin was identified as the primary mode and the surrounding monotonically decreasing region balanced around the mode was used for maximum likelihood estimation of mean and variance (scipy v0.19.1) [43]. Low-coverage regions for primary replicons within each metagenome were identified as regions (> 1 kb) with coverage below a stringent cutoff and defined by the overall mean for the replicon minus three standard deviations. For contig-based relative taxon abundance and clustering analyses, metagenome (Additional file 2: Table S2) contigs were aligned against the NCBI non-redundant protein database (ftp://ftp.ncbi.nlm.nih.gov/blast/db/FASTA/nr.gz) using the LAST alignment tool, followed by taxonomic assignment using MEGAN 6 long reads algorithm [45, 46]. Species abundances were calculated by summing the coverages of contigs assigned to species level. For each sample, species abundances from different filter fractions were averaged. The relative species abundances were calculated as percentages of the total species abundances. Data were reported for *Hht*. *litchfieldiae*, *Hrr*. *lacusprofundi*, DL31 and DL1, with all other species grouped as other archaea, bacteria, eucaryotes or viruses and projected as a scatter plot. The relative abundances were used for clustering and scaling analyses using Primer v7 [47]. Rauer 1 Lake was excluded because domain-level abundance of archaea was negligible (0.3%). The data were transformed using a square root transformation and a Bray-Curtis similarity matrix was used to assess the resemblance between samples. Unweighted pair group method with arithmetic mean (UPGMA) was used for clustering samples based on their similarities, resulting in a dendrogram with samples as leaves. A non-metric multi-dimensional scaling (nMDS) plot based on the Bray-Curtis similarity between samples was generated using recommended settings to show the two-dimensional positioning of each sample. The UPGMA cluster was overlaid on the nMDS plot to provide similarity readings. For contig recruitment to replicons, contigs ≥ 1 kb from each metagenome were compared to the replicons of the genomes of *Hht*. *litchfieldiae* tADL, *Hrr*. *lacusprofundi* ACAM34 and R1S1, DL31 and DL1 using nucmer from the MUMMER 3 toolkit [48]. Only hits spanning at least 5 kb and with ≥ 80% nucleotide identity were considered. The percentage of genome covered by metagenome contigs was calculated based on the hits identified by nucmer cumulated over the entire replicon. The corresponding read coverage was calculated by summing the number of reads mapped to all contigs with a nucmer hit to the replicon and expressed as a percentage of the total number of reads mapped to all contigs. Read mapping was computed with bbmap (https://sourceforge.net/projects/bbmap/, default parameters).

### Viral infection

*Hrr*. *lacusprofundi* ACAM34 and R1S1 were grown in modified-DBCM2 medium, cells infected with DLHTHV, and electron microscopy performed as described previously [24]. The virus was propagated and lysate obtained for infection studies using strain ACAM34. Virus particles (in growth medium) or growth medium (negative control) were mixed with host cultures at a multiplicity of infection of 1 and incubated for 3 h at room temperature. Samples (100 μl) were diluted into 40 ml fresh medium and incubated with shaking (120 rpm) at 30 °C. Growth was monitored as optical density (OD) at 600 nm, with starting OD adjusted to 0.05. Cells from 2 ml of uninfected and infected cultures were harvested after 3 days by centrifugation at 8000×*g*, and cell pellets were washed twice with growth medium to remove residual free virus particles. DNA was extracted from cells and infection analysed by PCR as described previously [24], using primers specific to an 890 bp region of the virus (5′-GAGCCTGCAGAAGAGCCCGATC and 5′-GAGTCGGTGGTCTGCGTGATCTC). Plaque assays were performed by incubating lysates with host cells for 1 h at room temperature, performing soft agar (4% agar, 50 °C) overlays on modified-DBCM2 solid medium (16% agar) and assessing plaque formation after 6–8 days incubation at 30 °C.

## Results

### Comparative analysis of R1S1 and ACAM34 primary replicons

Sequencing and assembly of *Hrr*. *lacusprofundi* R1S1 DNA produced a draft genome comprising 47 contigs (Table 1). The largest contig was generated via manual gap closure and represents a 2.7-Mb replicon matching the ACAM34 primary replicon. Aside from one 80 bp stretch of high GC DNA that could not be sequenced, the primary replicon is completely sequenced. ANI was 99.8 over 98% of encoded gene sequences, GC content was ~ 67% and each replicon possessed two rRNA gene clusters.

**Table 1:** Genome characteristics of *Hrr*. *lacusprofundi* strains ACAM34 and R1S1

	ACAM34	R1S1
Whole genome		
Genome (kb)	3693	3468
GC content (%)	64.0	64.7
Number of DNA scaffolds	3	47
Number of protein-coding genes	3665	3501
Primary replicon		
Primary replicon size (kb)	2735	2698
GC content (%)	66.7	66.8
Number of protein-coding genes	2745	2700
Number of rRNA gene clusters	2	2
ANI	99.8% identity over 98% of encoded genes	
Secondary replicons		
Number of replicons/contigs	2 circular replicons	46 linear contigs
Size (kb)	957 (525 and 431)	769
GC content (%)	55 and 57	43–62; 57 average
Number of protein-coding genes	920	801
ANI	97% identity over 30% of encoded genes	
Length of shared sequences (kb)	240 kb	
Length of unique sequences (kb)	717	529
Number of unique genes	651	544

**Fig. 2:**
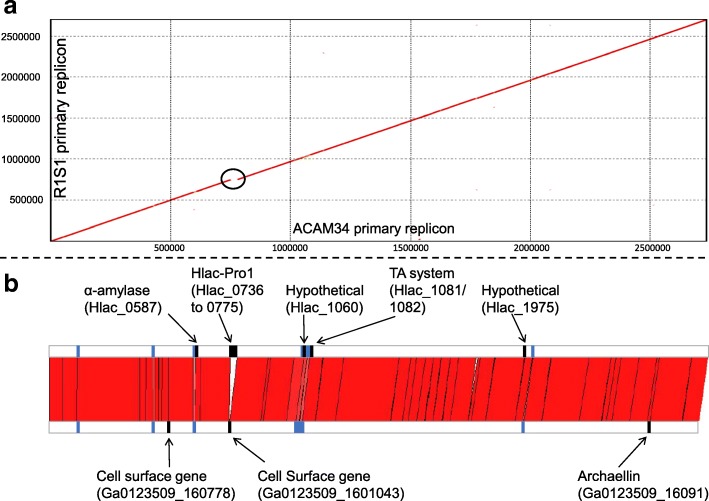
High similarity between R1S1 and ACAM34 primary replicons. **a** NUCmer plot [29] of R1S1 and ACAM34 primary replicons. The black circle highlights the Hlac-Pro1 provirus that is absent in R1S1. **b** Synteny between R1S1 and ACAM34 primary replicons. The red area connects sequences of the ACAM34 (upper horizontal bar) and R1S1 (lower horizontal bar) primary replicon that share high nucleotide identity: identity was > 99% with the exception of five regions with < 99% identity (blue bars; see Additional file 2: Table S6). Unique genes are highlighted as annotated black bars (Additional file 2: Table S4). In order to commence the alignment at the same sequence for both replicons, 80 ‘Ns’ were added to the end of the R1S1 replicon (to represent the unsequenced nucleotides) and the first 289,989 nucleotides were relocated to the end of the replicon

The primary replicon of R1S1 was ~ 37 kb shorter with 45 fewer genes than ACAM34. Other than this, the replicons were highly syntenic with no major rearrangements (Fig. 2). Sequences unique to a primary replicon included the Hlac-Pro1 provirus, 27 transposase genes, eight protein-coding genes from seven distinct loci (Fig. 2; Additional file 2: Tables S3, S4) and a number of short duplications and non-coding RNAs (Additional file 2: Table S5). R1S1 lacks Hlac-Pro1 (Fig. 2), which is 29 kb in length, consists of 38 predicted ORFs (many of which are similar to the BJ1 virus) and is thought to be defective [49]. Seventeen transposase genes were in unique locations in the primary replicon of ACAM34 and ten in R1S1, with three from both strains disrupting ORFs (Additional file 2: Table S3). In total (not considering transposase genes), strain-specific sequences accounted for only 5 and 32 kb of the R1S1 and ACAM34 primary replicons, respectively.

Genes unique to ACAM34 included two that form a putative toxin-antitoxin (TA) system, plus the only unique gene with an assigned metabolic function, an α-amylase (Additional file 2: Table S4). Three genes encoding predicted cell surface proteins were unique to R1S1, with one located where Hlac-Pro1 was integrated in ACAM34. Another was an archaellin gene (*flaB*), providing R1S1 with consecutive archaellin genes (Ga0123509_16091/16092) compared to only one (Hlac_2557) in ACAM34. Hlac_2557 is 98% identical to Ga0123509_16092, they both have 43–44% identity with Ga0123509_16091 and all three archaellin sequences share a conserved N-terminal region (amino acids 1–55) (Additional file 2: Figure S1). Ga0123509_16091 is 100% identical to a protein detected in a metaproteomic study of Deep Lake [6], with the corresponding metagenomic contig possessing both of the archaellin genes encoded by R1S1.

The majority of the two primary replicons, including intergenic regions, shared 99–100% sequence identity. Only five genomic regions (> 1 kb) with conserved gene content had < 99% sequence identity (Fig. 2; Additional file 2: Table S6), and all five regions contained one or more genes encoding cell surface proteins or proteins involved in the biosynthesis of cell surface structures. The S-layer glycoprotein had the lowest identity (54%). The largest region with < 99% sequence identity (region 4 in Additional file 2: Table S6) was ~ 37 kb in length and contained multiple genes predicted to perform N-glycosylation of cell surface structures (e.g. S-layer and archaella), including the oligosaccharyltransferase *aglB* which is the most conserved component of the N-glycosylation pathway in archaea [50].

### Analysis of *Hrr*. *lacusprofundi* secondary replicons

**Fig. 3:**
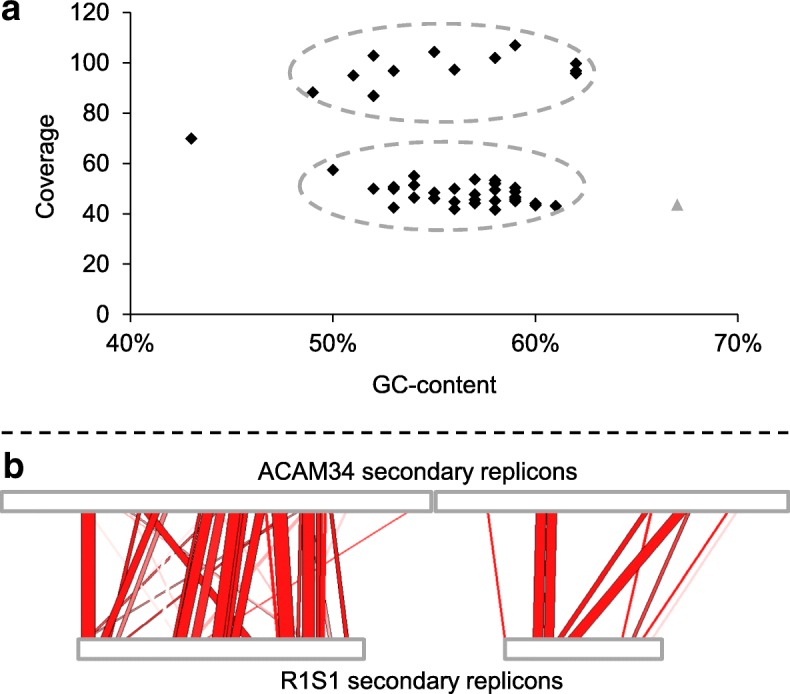
Analysis of secondary replicons. **a** GC/coverage plot of R1S1 contigs representing the secondary replicons (black diamonds) and primary replicon (single grey triangle). Clusters of contigs forming putative secondary replicons of ~ 220 (coverage of 42–57) and ~ 545 kb (coverage of 87–107) are highlighted with hatched ovals. The single contig outside of the two clusters (43% GC, coverage of 70) encoded four genes annotated as DNA methyltransferase, restriction endonuclease, phage integrase, and hypothetical protein. Not included are two small contigs (1 and 1.3 kb) with high coverage (352 and 162), encoding a transposase and an ATPase, respectively. **b** Contig mapping of R1S1 contigs to ACAM34 secondary replicons. The red area connects sequences of the ACAM34 (upper horizontal bar) and R1S1 (lower horizontal bar) secondary replicons that share ≥ 80% nucleotide identity, with regions of higher identity shown in darker red. For R1S1, the two horizontal bars represent concatenations of contigs containing mapped sequences (R1S1 contigs not mapping to ACAM34 secondary replicons are not shown). The panel highlights the low degree of conservation between R1S1 and ACAM34 secondary replicons. Mapping was performed with CONTIGuator [31] and visualised using ACT [30]

Most of the 46 additional contigs (total 769 kb) that were not part of the primary replicon could be separated into two distinct clusters, with 31 contigs (total 545 kb) having a read depth of 42–57 and 12 contigs (total 217 kb) a read depth of 87–107 (Fig. 3a). Therefore, R1S1 appears to be similar to ACAM34 in containing two secondary replicons, with the smaller R1S1 replicon having a higher copy number than the larger one. Most (98.6%) of the 1.4 million sequencing reads mapped onto the assembled contigs (primary plus secondary replicons), indicating that the secondary replicons were almost complete. For subsequent analyses of the R1S1 secondary replicons, the 46 contigs were pooled.

The average GC content of the R1S1 and ACAM34 secondary replicons was similar (~ 57%) and ~ 10% lower than the primary replicon (~ 67%, Table 1). The ANI between ACAM34 and R1S1 secondary replicons was high (97%), but this was calculated on only ~ 30% of genes which aligned with ≥ 30% sequence identity over ≥ 70% of their length (Table 1). The low proportion of conserved genes was also reflected by contig mapping which found only 240 kb of the 769 kb of R1S1 contig sequences aligned to the ACAM34 secondary replicons (Fig. 3b), designating 529 and 717 kb of the secondary replicons as unique to their respective strains (Table 1). Despite the differences in gene content, the representation of arCOGs functional classes [33] was broadly similar between the ACAM34 and R1S1 secondary replicons (Additional file 2: Figure S2).

The sequences unique to the R1S1 secondary replicons were used to search for HIRs shared with Deep Lake species *Hht*. *litchfieldiae* tADL, DL31 and DL1. Eleven regions, 2–14 kb in length, were identified, including a 3.7-kb region that was common to R1S1, *Hht*. *litchfieldiae* tADL and DL1 (Additional file 2: Table S7, Figure S3). In total, these regions represent 67 kb of HIRs that are specific to R1S1 (i.e. not ACAM34) and the other three Deep Lake genera. Similar to ACAM34, the HIRs unique to R1S1 mapped to secondary replicons of DL31 and DL1 (Additional file 2: Table S7) [5]. For *Hht*. *litchfieldiae* tADL, which contains a single replicon, the R1S1 HIRs mapped to regions of the genome where HIRs were previously identified (Additional file 2: Figure S4) [5].

A type I-B *cas* gene cluster with an adjacent 69 spacer CRISPR array was identified on a R1S1 secondary replicon contig (Additional file 2: Table S8). However, rather than being similar to the ACAM34 type I-B system, the R1S1 CRISPR locus is nearly identical to the DL1 CRISPR (also located on a secondary replicon) (Additional file 2: Table S8). The 30 nucleotide repeat sequence and six of the Cas sequences are 100% identical, and the two other Cas sequences are 99% identical. The CRISPR/Cas region represents a 9388 bp HIR (Additional file 2: Table S8) that is shared between the R1S1 strain of *Hrr*. *lacusprofundi* and DL1. While the R1S1 and DL1 Cas and repeat sequences have high identity, none of the spacer sequences are conserved. Analysis of the spacers from the R1S1 and ACAM34 type I-B system showed that one of the R1S1 spacers matched Hlac-Pro1, while the spacers from ACAM34 did not (Additional file 2: Table S9). Conceivably, the R1S1 spacer may provide immunity to Hlac-Pro1-related viruses.

### Analysis of susceptibility to infection by DLHTHV

**Fig. 4:**
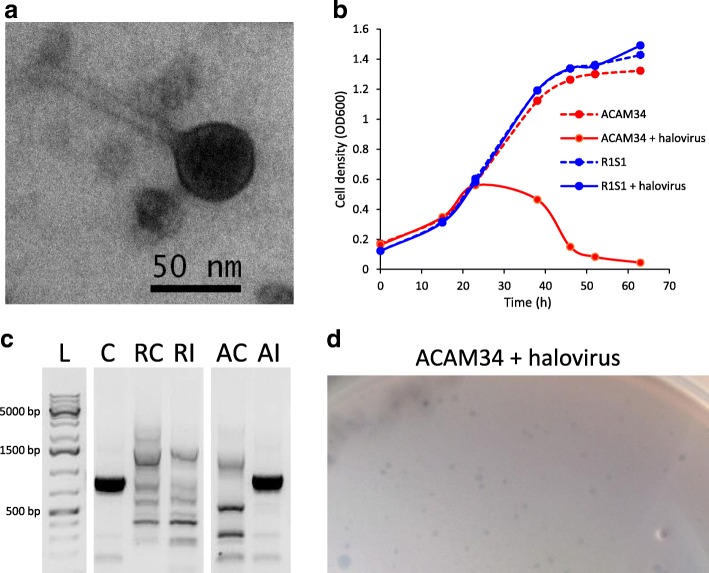
Infection of ACAM34 and R1S1 with Antarctic halovirus DLHTHV. **a** Transmission electron micrograph of the halovirus. **b** Effect of halovirus infection on growth of ACAM34 and R1S1. Growth retardation was observed during infection of ACAM34 but not R1S1. **c** Confirmation of infection of ACAM34 using PCR specific to the halovirus. L GeneRuler 1 kb Plus DNA Ladder (Thermo Fisher Scientific), C purified halovirus DNA control, RC R1S1 uninfected, RI R1S1 infected, AC ACAM34 uninfected, AI ACAM34 infected. The halovirus-specific PCR product is visible as a thick black band (lanes C and AI). The same concentration of template DNA was used for all samples. The original gel image was modified by removing gel lanes (indicated by gaps) to improve visual presentation. **d** Plaque assay showing plaques formed (small zones of clearing) from infection of ACAM34 with the halovirus. No plaques were formed with infection of R1S1

To assess whether strain differences in cell surface proteins (primary replicons) and/or type I-B CRISPR systems (secondary replicons) might confer differential susceptibility to viruses, DLHTHV that was recently isolated from Deep Lake (Erdmann and Cavicchioli, unpublished results; see the ‘Methods’ section) was used for infection studies (Fig. 4a). Co-incubation with the virus did not negatively impact the growth of R1S1 but did result in strong growth retardation of ACAM34 (Fig. 4b). PCR analysis using primers specific to the virus with DNA extracted from the strains after infection resulted in a product from ACAM34 but not R1S1 (Fig. 4c). Furthermore, plaques formed when ACAM34 was infected with the virus but did not form when R1S1 was incubated with the virus (Fig. 4d). The data demonstrate a clear difference in the susceptibility of the two strains to this halovirus.

### Metagenome analysis of community composition

To examine taxonomic and genomic variation in the environment, assembled metagenomes from six Antarctic hypersaline lakes were analysed (Fig. 1, Additional file 2: Table S1). Metagenomes were generated from biomass collected by sequential size filtration using water collected from four lakes from the Rauer Islands (Rauer 1, 3, 6 and 13 lakes) and two lakes from the Vestfold Hills (Deep Lake and Club Lake). The samples were collected during the austral late-spring/summer of 2013–2015. In addition, for Deep Lake, samples were collected during winter 2014 and summer 2006 and 2008. In total, 33 metagenomes were generated representing ~ 5.2 Gb of sequence data (Additional file 2: Table S2).

*Hrr*. *lacusprofundi* 16S rRNA gene sequences (Additional file 2: Table S10) and the genes unique to each *Hrr*. *lacusprofundi* strain (Table 2) were detected in metagenomes from all lakes except Rauer 1 Lake. Strain R1S1 was isolated from Rauer 1 Lake water that was sampled September 2014, but the metagenome was generated from January 2015 biomass. R1S1 was cultivated from a laboratory enrichment [24], so the species may genuinely be a minor component of the Rauer 1 Lake community.

**Table 2:** Presence of genes unique to ACAM34 and R1S1 in metagenomes from six Antarctic hypersaline lakes

Genes unique to ACAM34	Deep Lake (17)		Club Lake (4)		Rauer 1 Lake (3)		Rauer 3 Lake (3)		Rauer 6 Lake (3)		Rauer 13 Lake (3)		Genes unique to R1S1
Single archaellin	0	17	0	4	0	0	0	3	0	3	0	3	Two archaellins
No	0	17	0	4	0	0	0	2	0	3	0	3	Cell surface protein (Ga0123509_160778)
α-Amylase	17	17	4	4	0	0	3	1	1	3	0	3	No
Provirus Hlac-Pro1	0	17	0	4	0	0	0	3	1	3	0	3	Cell surface protein (Ga0123509_1601043)
ArsR-like protein (Hlac_1060)	17	17	4	4	0	0	2	2	3	2	3	3	No
TA system	17	17	4	4	0	0	2	3	3	3	3	3	No
Hypothetical protein (Hlac_1975)	0	0	0	0	0	0	0	0	1	0	0	0	No

The genes unique to a strain and the gene content present at the same location in the other strain (plus ~ 500 bp either side of the locus) (Additional file 2: Table S4) are shown as the first and last columns. When no gene was present in a strain at the position of a unique gene in the other strain, ‘No’ was stated. The number of metagenomes analysed for each of the six lakes is shown in parentheses. Numbers in columns below each lake indicate the number of metagenomes that contained unique genes or the corresponding loci from the other strain, for ACAM34 (left column) and R1S1 (right column). Hlac_1060 is common in haloarchaea and has an ArsR-like helix-turn-helix domain (DNA-binding) that is often present in metal-regulated transcription regulatory proteins. Hlac_1975 has no identifiable domains (using InterProScan [66])

**Fig. 5:**
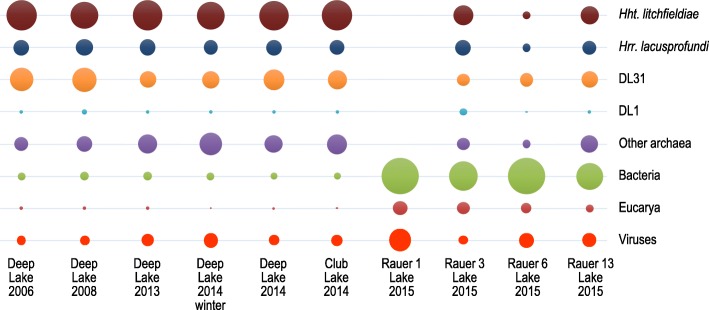
Relative abundance of lake taxa assessed from read coverage and taxonomic assignment of contigs assembled from metagenome data. The scatter plot depicts the relative species abundances of taxa in five samples from Deep Lake, one from Club Lake, and one sample each from lakes in the Rauer Islands (Rauer 1, 3, 6 and 13). Relative abundances are directly proportional to the sizes of the circles in the plot. All samples are from summer except the sample labelled Deep Lake 2014 winter (Additional file 2: Table S1). Abundances were obtained from the coverages of the metagenome contigs assigned to species level and relative abundances shown as percentages of the total species abundance for each sample (Additional file 2: Table S12). Data are shown for *Hht*. *litchfieldiae*, *Hrr*. *lacusprofundi*, DL31 and DL1, with all other species grouped as other archaea, bacteria, eucarya or viruses

*Hht*. *litchfieldiae* tADL and DL31 16S rRNA gene sequences were present in the same lakes as *Hrr*. *lacusprofundi*, while DL1 sequences were present in four of the same lakes (Additional file 2: Table S10). The median read coverage values of the primary replicons of *Hrr*. *lacusprofundi* ACAM34, *Hht*. *litchfieldiae* tADL, DL31 and DL1 in the nine pooled metagenomes (see the ‘Methods’ section) was used to assess the relative abundance of the four species (Additional file 2: Table S11). The relative abundance of the overall lake taxa was further assessed from read coverage and taxonomic assignment of contigs assembled from the metagenome data (see the ‘Methods’ section) (Fig. 5; Additional file 2: Table S12). The high representation of *Hht*. *litchfieldiae*, DL31 and *Hrr*. *lacusprofundi* in all lakes except Rauer 1 Lake was apparent, with bacteria and to a lesser degree eucaryotes contributing more to the Rauer Islands lake communities.

### Metagenome analysis of *Hrr*. *lacusprofundi* genomic variation

The prevalence of *Hrr*. *lacusprofundi* strain-specific loci was assessed by searching for the alleles of the eight protein-coding genes from the seven loci plus the Hlac-Pro1 virus (Table 2; Additional file 2: Table S4). The three cell surface protein genes unique to R1S1, including the tandem archaellin genes, were present in 29–30 metagenomes, whereas the corresponding ACAM34 alleles were not present in any of the metagenomes (Table 2). The ACAM34 Hlac-Pro1 genes were only present in a single metagenome from Rauer 6 Lake. In contrast, the TA system and an ArsR-like gene (Hlac_1060) specific to ACAM34 were present in most metagenomes (30) along with the corresponding R1S1 alleles (30 and 28). The ACAM34 specific α-amylase gene and the corresponding R1S1 allele were both present in Deep Lake and Club Lake metagenomes. In the Rauer Island lakes, the representation of the R1S1 α-amylase allele was higher in Rauer 3 Lake (R1S1 allele in 3/3 metagenomes vs ACAM34 allele in 1/3 metagenomes) and Rauer 13 Lake (R1S1 allele in 1/3 vs ACAM34 allele in 0/3) but equivalent in Rauer 6 Lake (both alleles in all three metagenomes). The ACAM34 specific hypothetical gene Hlac_1975 was not present in any of the metagenomes, and the corresponding R1S1 allele was only found in one metagenome from Rauer 6 Lake.

This analysis of strain-specific loci (Table 2) shows marked differences occurred in allelic representation. The R1S1 cell surface proteins and the tandem archaellins dominated the lake populations in both the Vestfold Hills and Rauer Islands, whereas a more equal representation of the alleles for the TA system and ArsR-like gene Hlac_1060 occurred across the same lakes. Different again was the representation of Hlac-Pro1 and the hypothetical gene Hlac_1975 which were evidently uncommon within the lake populations, while the representation of the α-amylase allele was variable, with regional and lake-specific patterns present.

### Metagenome analysis of genomic variation between haloarchaeal genera

**Fig. 6:**
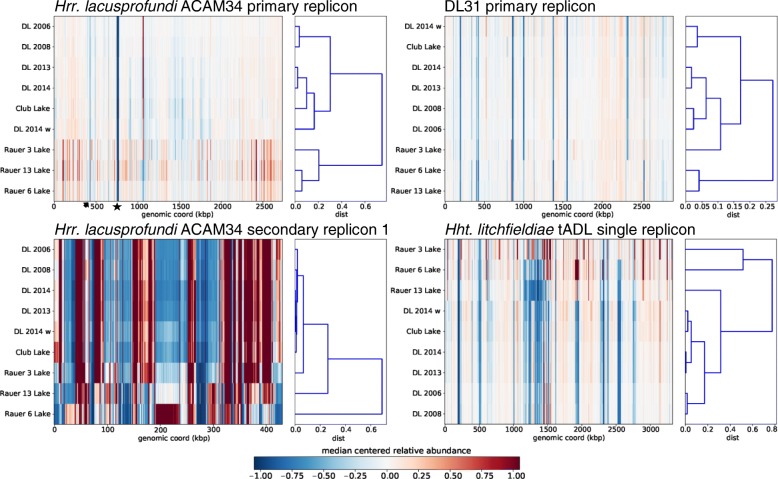
Genomic islands and biogeographic patterns of haloarchaea in hypersaline lakes from the Vestfold Hills and Rauer Islands. Reads from nine pooled metagenomes (Additional file 2: Table S11) were mapped onto the primary and secondary replicons of *Hrr*. *lacusprofundi* ACAM34, DL31 and *Hht*. *litchfieldiae* tADL. For a given reference sequence (replicon), the heat map shows centred and scaled by per location, median depth of coverage for each metagenome. Columns represent genomic bins on the reference, while rows represent depth of coverage for each geographic location. Hierarchical clustering of the correlation matrix was used to order rows and the resulting dendrogram is shown on the right. The heat-maps for the primary replicons highlight the differences in genomic islands present on primary replicons between the three species (also see coverage plots in Additional file 2: Figure S5). Features on genomic islands of the ACAM34 primary replicon are highlighted: provirus Hlac-Pro1 (star), S-layer gene (arrow). The heat map for the ACAM34 secondary replicon consists mainly of regions with either high or low coverage, highlighting high variability of secondary replicons within populations (also see the equivalent plots for the other secondary replicons in Additional file 2: Figure S6). The HCA reveals biogeographical clusters distinguishing the Rauer Island lakes from the Vestfold Hills lakes. All metagenomes were from summer except for Deep Lake 2014 winter (w). DL Deep Lake

The read coverage information (except for DL1 for which coverage was too low) was used to identify genomic islands (low-coverage regions) present within the populations (Additional file 2: Figure S5). On average, only ~ 31 kb (~ 1%) of the ACAM34 primary replicon had low coverage in each of the pooled metagenomes (Fig. 6; Additional file 2: Figure S5). The main contributors were Hlac-Pro1 (~ 21 kb; low coverage in all metagenomes) and the S-layer gene (low coverage in eight of nine metagenomes). In contrast, the primary replicons of DL31 and *Hht*. *litchfieldiae* had a higher proportion of low coverage, ~ 161 kb (5.5%) and ~ 433 kb (13%), respectively (Fig. 6; Additional file 2: Figure S5). Compared to the primary replicons, the metagenome read mapping of the secondary replicons was uneven with areas of both low and high coverage (Fig. 6; Additional file 2: Figure S6), indicative of high variability of secondary replicon genomic content within the lake populations.

**Fig. 7:**
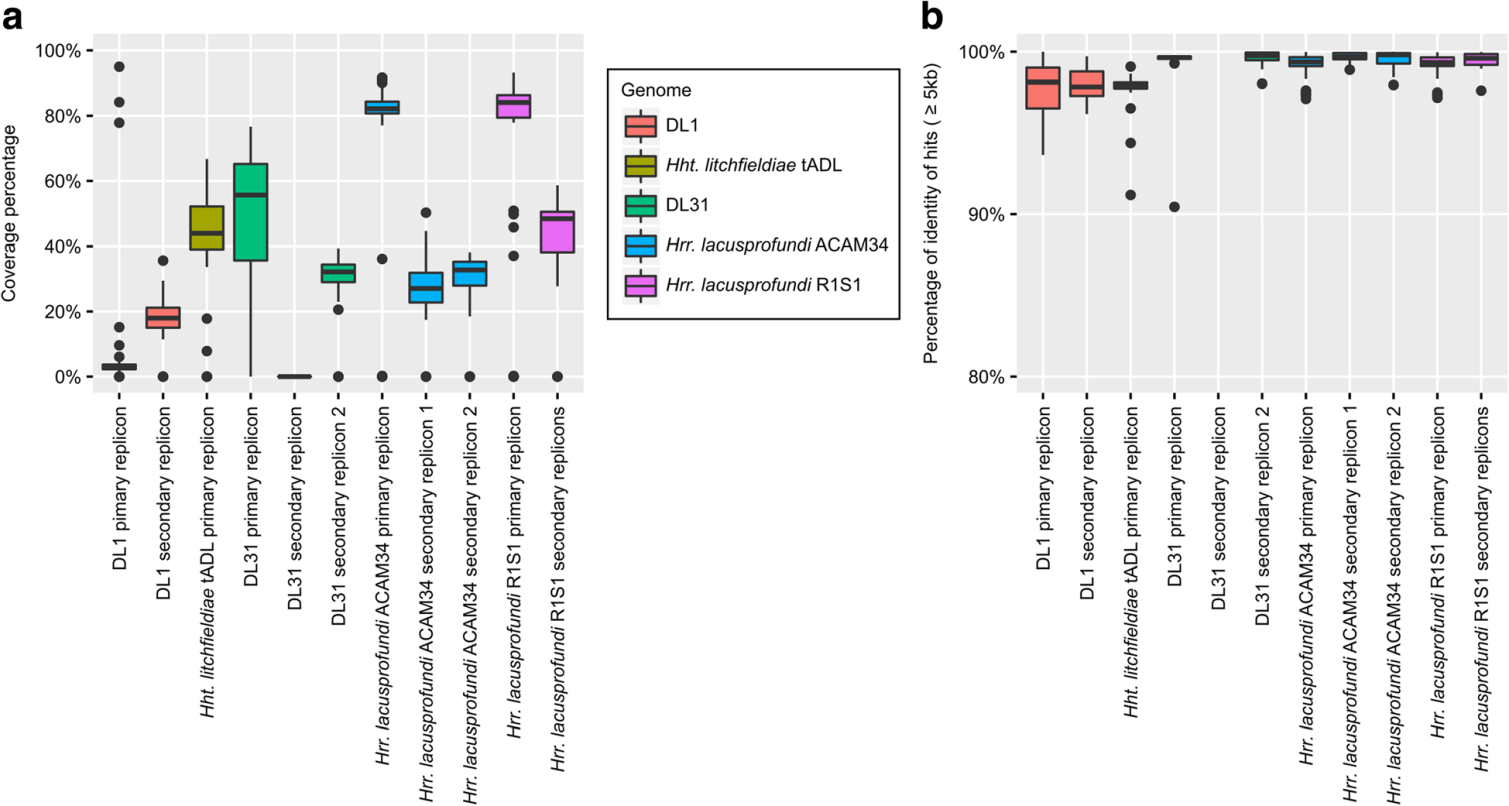
Mapping of de novo assembled metagenome contigs to the replicons of DL1, *Hht*. *litchfieldiae* tADL, DL31, *Hrr*. *lacusprofundi* ACAM34 and *Hrr*. *lacusprofundi* R1S1. **a** Sequence coverage for each replicon expressed as the percentage of the replicon covered by contigs assembled de novo from metagenomes. Coverage was calculated separately for each metagenome (Additional file 2: Table S2) for each replicon, except for R1S1 secondary replicons where the coverage was calculated as the average across all secondary contigs (Additional file 2: Table S13). Mapping of metagenome contigs to replicons is shown in Additional file 2: Figure S7. **b** Percentage of nucleotide identity for hits ≥ 5 kb between contigs and reference replicons, averaged by metagenome. **a**, **b** Lower and upper hinges correspond to the first and third quartiles, whiskers extend no further than ± 1.5 × inter-quartile range and outliers are shown as dots

Contigs assembled from the metagenome data were mapped to the replicons of *Hrr*. *lacusprofundi* R1S1 and ACAM34, and *Hht*. *litchfieldiae* tADL and DL31 (Fig. 7; Additional file 2: Figure S7). The pattern of contig mapping was similar to the read coverage results (Fig. 6; Additional file 2: Figures S5, S6). Contigs covered 84 and 82% of the R1S1 and ACAM34 primary replicons, respectively, but only 56 and 44% of the primary replicons of DL31 and *Hht*. *litchfieldiae*, respectively (Fig. 7a; Additional file 2: Table S13). In contrast, contig coverage of all the secondary replicons was considerably lower (Fig. 7a; Additional file 2: Table S13). The average sequence identity of recruited contigs was ≥ 98% for all primary replicons across all metagenomes, confirming that the regions of the replicons covered by the contigs are very stable (Fig. 7b; Additional file 2: Table S13).

### Biogeographical and temporal variation

Hierarchical cluster analysis (HCA) of the read coverage distributions for the nine pooled metagenomes was used to determine what relationship existed between populations of *Hrr*. *lacusprofundi*, *Hht*. *litchfieldiae* and DL31 from the different lakes and sampling periods (Fig. 6; Additional file 2: Figure S6). Even though the primary replicon of *Hrr*. *lacusprofundi* had comparatively little genomic (Fig. 2) or metagenomic variation, HCA revealed distinct clustering (Fig. 6). Moreover, the clustering patterns were similar for the replicons of *Hrr*. *lacusprofundi*, *Hht*. *litchfieldiae* and DL31 (Fig. 6; Additional file 2: Figure S6). UPGMA clustering and nMDS analysis that was based on species abundances obtained from metagenome contig coverages revealed a similar relationship between the sampling sites (Additional file 2: Figure S8). The biggest difference between clusters related to biogeography: separation between populations in the Vestfold Hills vs the Rauer Islands lakes. Temporal differences were evident for Deep Lake summer metagenomes with the earlier dates (2006 and 2008) more similar to each other than the recent dates (2013 and 2014). Moreover, Club Lake which was sampled in summer 2014 clustered with Deep Lake metagenomes from the 2013–2014 period.

## Discussion

### Genomic variation—response to viruses

The genomic comparison between R1S1 and ACAM34 revealed highly conserved primary replicons (Fig. 2) and genetically variable secondary replicons (Fig. 3b). Many of the genomic differences appear to relate to interactions with viruses. Proviruses have previously been identified in haloarchaeal genomes [49], including distinct proviruses in different strains of *Hqr*. *walsbyi* [9]. Consistent with this, the presence/absence of Hlac-Pro1 in ACAM34/R1S1 represented the largest contiguous sequence difference between the primary replicons. Unlike ACAM34, the R1S1 CRISPR spacers include a match to Hlac-Pro1 (Additional file 2: Table S9) and may therefore provide immunity to the virus and perhaps explain why it is not integrated in the R1S1 primary replicon.

Infectivity is also likely to be affected by the cell surface differences of the strains, particularly the S-layer glycoprotein which exhibited only 54% identity; this type of variation has previously been reported for Deep Lake haloarchaea [6]. Cell surface variation of haloarchaea is generally inferred to be a response to virus infection pressure [8, 9, 11], consistent with observations for environmental bacteria such as marine *Prochlorococcus* spp. where strains can accumulate mutations in cell surface genes after exposure to infecting viruses [4].

Variation of *Hrr*. *lacusprofundi* cell surface may also arise from variant glycosyltransferases and the oligosaccharyltransferase AglB (Additional file 2: Table S6). The posttranslational attachment of glycans to cell surface structures by N-glycosylation is characteristic of haloarchaeal S-layer and archaella proteins [50–52] and is important for protein stability [53, 54]. It can also occur on haloarchaeal viral proteins and affect the recognition of host cell surface receptors [55]. In the methanogen *Methanococcus voltae*, changes in glycosylation were speculated to derive from mutations in genes involved in glycan synthesis or attachment [50, 56], and mutation of bacterial glycosyltransferase genes was found to change substrate specificity and affect the sugars utilised for glycosylation [57]. Genes thought to be involved in the glycosylation of cell surface proteins have also been identified on a *Hqr*. *walsbyi* genomic island [8]. It is therefore possible that the variation that exists in the genes involved in the N-glycosylation pathway within the *Hrr*. *lacusprofundi* population increases the variety of glycan compositions that can be attached to cell surface proteins, thereby altering susceptibility to infecting viruses.

R1S1 also possesses tandem archaellin genes, and this allele is dominant in the lake populations compared to the single archaellin gene possessed by ACAM34 (Table 2; Additional file 2: Figure S1). Multiple copies of archaellin genes are not uncommon in haloarchaeal genomes [58], and *Hht*. *litchfieldiae* strain tADL contains a total of seven archaellin genes of which most are expressed in Deep Lake [6]. In *Haloarcula marismortui*, switching expression between two genes provides cells with distinct morphologies and antigenic properties [58]. Hence, if R1S1 does switch between archaellins, it could reduce infections by viruses that bind to specific structural features of archaella.

Differences between the R1S1 and ACAM34 secondary replicons included the distinct type I-B CRISPR systems. The replacement of entire CRISPR systems between strains has been observed in *Sulfolobus* [59]. However, it was noteworthy that the R1S1 CRISPR/Cas sequences represented a HIR shared with DL1. DL1 represents a relatively minor component in Antarctic hypersaline lake communities (Additional file 2: Table S12). Nevertheless, network analyses previously identified DL1 to be involved in extensive sharing of HIRs between genera in Deep Lake [5]. While frequent horizontal gene transfer of CRISPR systems has been inferred from phylogenetic analyses [60, 61], this Antarctic type I-B CRISPR system appears to be the first reported example of a virtually identical system present in axenic cultures of two distinct genera. The presence of distinct spacer sequences for each of the CRISPR system in R1S1 and DL1 indicates specific histories of responses to invasion.

Consistent with the genomic inferences, the Antarctic halovirus infection studies provided experimental evidence of the differential susceptibility of the strains (Fig. 4). The development of this infection system in combination with the development of genetics for *Hrr*. *lacusprofundi* [62] provides useful avenues for future research aimed at elucidating the roles of the specific host evasion and defence mechanisms.

### Genomic variation—lifestyle and biogeography

**Fig. 8:**
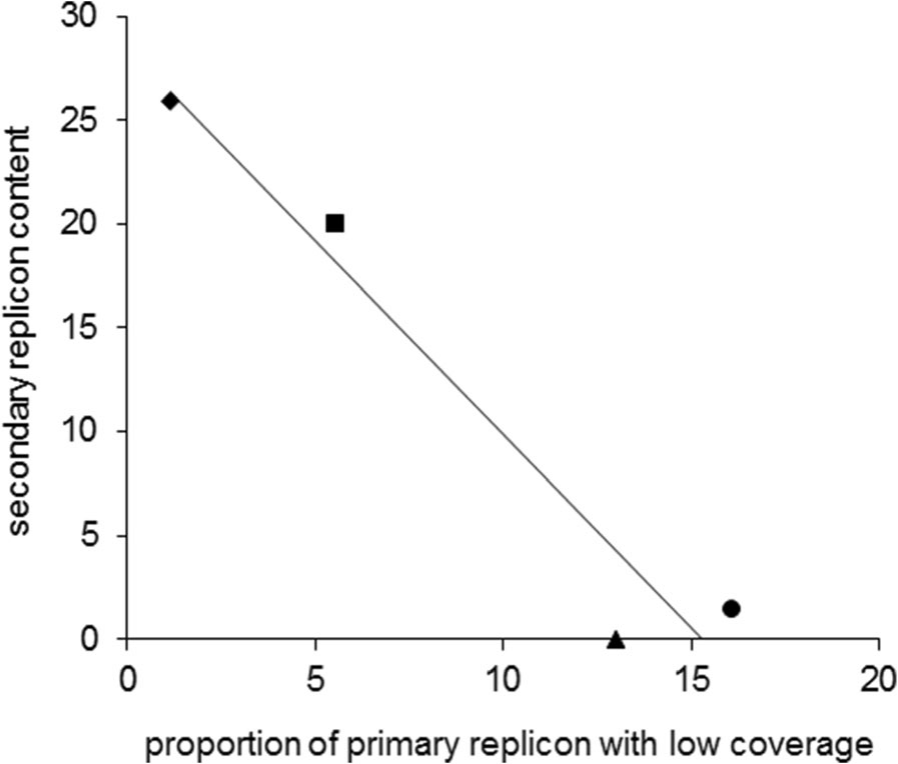
Relationship between genomic structuring and location of variation. Correlation between the proportion of the genome that is contained in secondary replicons and the percentage of the primary replicon that has low coverage. *Hrr*. *lacusprofundi* ACAM34 (black diamond), DL31 (black square), *Hht*. *litchfieldiae* tADL (black triangle), *Hqr*. *walsbyi* HBSQ001 (black square). For *Hqr*. *walsbyi* HBSQ001, low coverage corresponds to previously identified genomic islands [8]. The calculated correlation coefficient (*R*^2^) is 0.94

The genomic (Fig. 2) and metagenomic (Figs. 6, 7; Additional file 2: Figures S5, S7) analyses show that the primary replicon of *Hrr*. *lacusprofundi* is highly conserved (~ 1% variation between R1S1 and ACAM34 and 1% with low metagenome coverage), whereas the primary replicons of two *Hqr*. *walsbyi* strains were reported to possess ~ 10% variation and ~ 16% low metagenome coverage [8, 10]. In contrast to the primary replicons, the *Hrr*. *lacusprofundi* secondary replicons constitute a relatively large proportion of the genome (22–26%; Table 1) and they accommodate the bulk of the genomic variation (Table 1; Figs. 3b, 6, and 7; Additional file 2: Figures S6, S7), while the secondary replicons of *Hqr*. *walsbyi* represent only a small proportion of the genome (2–3%) [9]. DL31 is similar to *Hrr*. *lacusprofundi* in containing a large proportion of variable secondary replicon content (Additional file 2: Figures S5–S7) but contains a higher proportion of variable content on its primary replicon. *Hht*. *litchfieldiae* is a ‘minimalist’ in terms of replicon structuring as it possesses a single replicon which therefore contains all genomic variation (Figs. 6, 7; Additional file 2: Figures S5, S7). By comparing across these four haloarchaea, there appears to be a relationship between genomic structuring and location of variation. This becomes apparent when plotting the proportion of the genome that is present as secondary replicons vs the percentage of the primary replicon that has low coverage (Fig. 8). While genomic islands represent flexible genome content and likely confer adaptive traits including niche and viral adaptation [4–6, 8–10, 12, 63, 64], our analysis demonstrates that the way in which haloarchaea accommodate variability relates to the replicon structuring of their genomes.

The variation observed in *Hrr*. *lacusprofundi* strain-specific alleles (Table 2) likely reflects regional (Vestfold Hills vs Rauer Islands) and lake-specific environmental differences. The Rauer Island lakes are shallow and undergo significant seasonal changes including freezing in winter and being subject to potentially large changes in salinity from snow melt and evaporation (Additional file 2: Table S1) [19]. In contrast, Deep and Club lakes do not freeze and are more physically stable, large and deep aquatic systems. The clustering analyses describe a pattern of genomic variation across the lakes that is consistent for the three dominant Antarctic haloarchaeal genera (Fig. 6; Additional file 2: Figures S6, S8), with the biggest factor distinguishing populations being geographic location. Conceivably this variation could be explained by the marked regional limnological differences, as well as by distance and a barrier (Sorsdal Glacier; Fig. 1) affecting dispersal.

## Conclusions

In this study, we demonstrated the relevance of replicon structuring in accommodating genomic variation and showed the importance of intergenera exchange of HIRs in shaping the genomic repertoire of Antarctic haloarchaeal communities. By providing evidence of HIR intergenera exchange outside of Deep Lake, the study demonstrated the broader contribution it makes to Antarctic haloarchaeal communities and identifies HIRs as distinctive features of the haloarchaeal pan-genome. Limnological distinctions were inferred to affect the genomic composition of the Antarctic haloarchaea, with the observed variation in genes encoding cell surface structures and the outcome of the virus infectivity studies particularly emphasising the importance of virus-host interactions.

Temperature, annual light cycle and geographic isolation are the biggest factors distinguishing the Antarctic systems from the rest of the world [14]. Antarctica itself contains 16 biologically distinct, ice-free regions [65]. It was previously shown that the community in Deep Lake lacks the high representation of species (*Haloarcula* spp., *Hfx*. *volcanii*, *Hqr*. *walsbyi* and *Hbt*. *salinarum*) that are typically found in non-Antarctic hypersaline environments [5]. In this study, by expanding assessments to Club and Rauer Island lakes, stronger evidence has been obtained that points to *Hrr*. *lacusprofundi*, *Hht*. *litchfieldiae*, DL31 and to a lesser degree DL1 being endemic to Antarctica. Advancing understanding of the haloarchaeal pan-genome and endemism will be greatly facilitated by identifying equivalent hypersaline systems in other regions of Antarctica (possibly in the McMurdo Dry Valleys) and in cold hypersaline systems elsewhere in the world (e.g. Tibetan Plateau) and characterising the genomes of the indigenous haloarchaea.

## Additional files

Additional file 1: **Supplementary results**: Sampling during the 2013–2015 season. (PDF 13433 kb)

Additional file 2: Figures S1-S8 and Tables S1-S13: **Figure S1.** Archaellin protein sequence alignment. **Figure S2.** arCOG functional classes of genes present on ACAM34 and R1S1 secondary replicons. **Figure S3.** arCOG functional classes of the genes within HIRs specific to R1S1 and DL1, DL31 and *Hht*. *litchfieldiae* tADL. **Figure S4.** New HIRs present in R1S1 that are shared with *Hht*. *litchfieldiae* tADL. Figure S5 Genomic islands on primary replicons of Antarctic haloarchaea. **Figure S6.** Metagenome coverage and HCA of selected secondary replicons. **Figure S7.** Contigs assembled from metagenomes mapped to replicons. **Figure S8.** Clustering and scaling of samples. **Table S1.** Description of the Rauer Islands and Vestfold Hills hypersaline lakes sampled in this study. **Table S2.** Antarctic lake metagenomes used in this study. **Table S3.** Unique transposases of ACAM34 and R1S1. **Table S4.** Unique protein-coding genes. **Table S5.** Unique sequence duplications and non-coding RNAs on the R1S1 primary replicon. **Table S6.** Regions with low sequence similarity between ACAM34 and R1S1 primary replicons. **Table S7.** New HIRs identified in R1S1. **Table S8.** HIR conserved between *Hrr*. *lacusprofundi* R1S1 and DL1 that encodes a type I-B CRISPR system. **Table S9.** R1S1 CRISPR spacer matching Hlac-Pro1 in ACAM34. **Table S10.** Presence of 16S rRNA gene sequences for known Antarctic haloarchaeal species in Antarctic hypersaline lakes. **Table S11.** Relative abundance of *Hrr*. *lacusprofundi* ACAM34, *Hht*. *litchfieldiae* tADL, DL31 and DL1 in Antarctic lake metagenomes. **Table S12.** Relative abundance of lake taxa assessed from read coverage and taxonomic assignment of contigs assembled from metagenome data. **Table S13.** Genome coverage and percent identity for contigs mapped to replicons of *Hrr*. *lacusprofundi* R1S1 and ACAM34, *Hht*. *litchfieldiae* tADL, DL31 and DL1. (PDF 7786 kb)
