# Supplementary material for: Genomic variation and biogeography of Antarctic haloarchaea
Source: Microbiome. 2018 Jun 20;6:113. doi: 10.1186/s40168-018-0495-3 (PMC6011602; doi:10.1186/s40168-018-0495-3)

Additional file 1

Genomic variation and biogeography of Antarctic haloarchaea

Bernhard Tschitschko, Susanne Erdmann, Matthew Z. DeMaere, Simon Roux, Pratibha Panwar, Michelle A. Allen, Timothy J. Williams, Sarah Brazendale, Alyce M. Hancock, Emiley A. Eloe-Fadrosh and Ricardo Cavicchioli

Supplementary Results

Sampling during the 2013 – 2015 season

Sampling during the 2013 – 2015 season.

Rauer Islands: Rauer 1 Lake (Filla Island) (photo credits: Alyce Hancock)

GPS Co-ordinates: S68˚48.49’, E077˚51.303’

Elevation: 11m

Date: 11/01/2015

Time: 1.15pm

Weather: Cloudy, slight wind (less than 5 knots), warm ~+2˚C

Water Temperature: 6 degrees (thermometer)

Approximate Depth of Water at Sampling Site: 2m (most of lake lake less than 30cm deep, a few small 2-3m depressions) - Sample taken by dipping the 25L drum over the edge of the depression.

Approximate Volume Filter: 50L filtered at 8pm on 11/01/2015 for 60min

Sediment type (bottom of lake): sandy/silty

Sediment type (edge of the lake): rocky/sandy

Observations: No ice on the lake or snow around the lake. Quite a bit of fine sediment in the lake, some on the 3.0um filter.

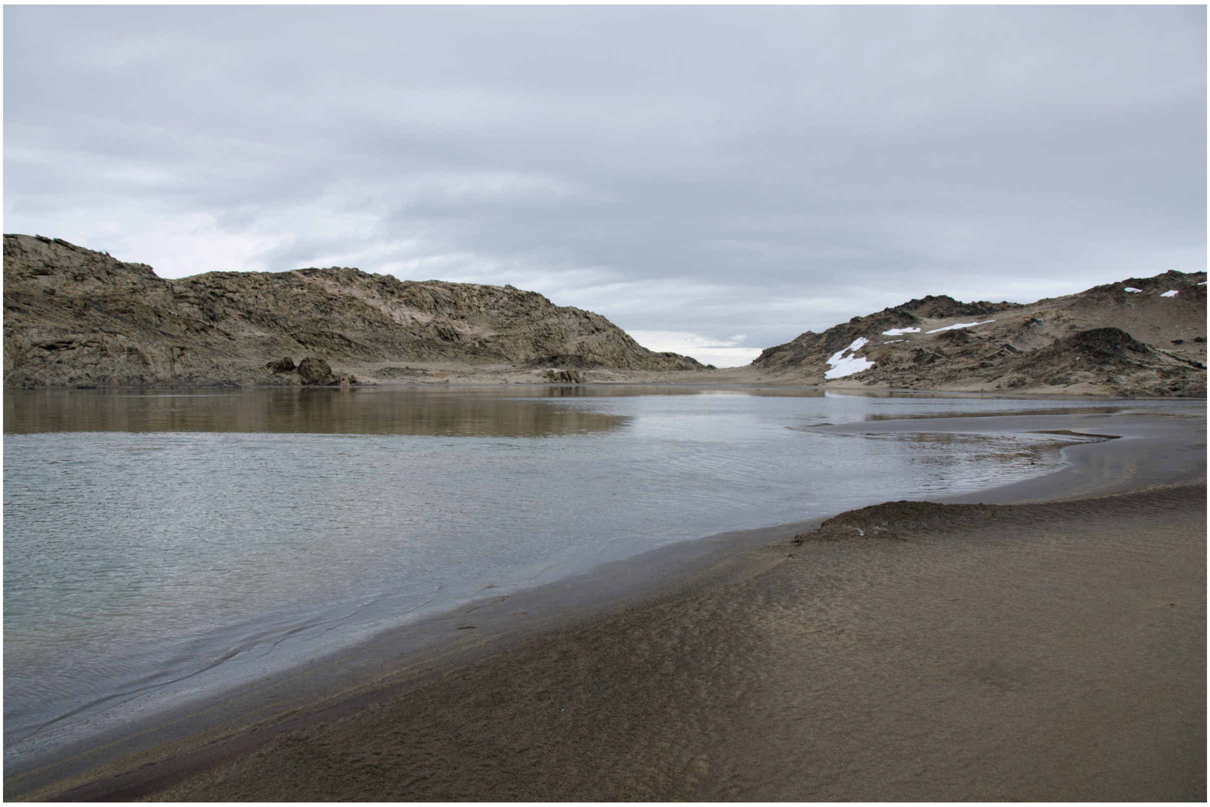

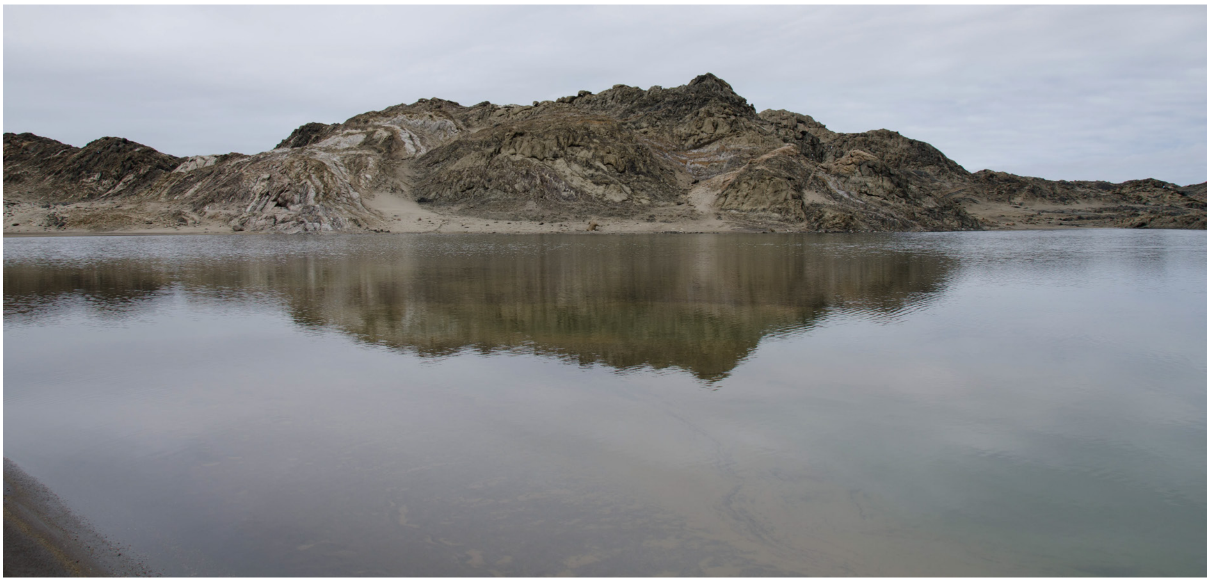

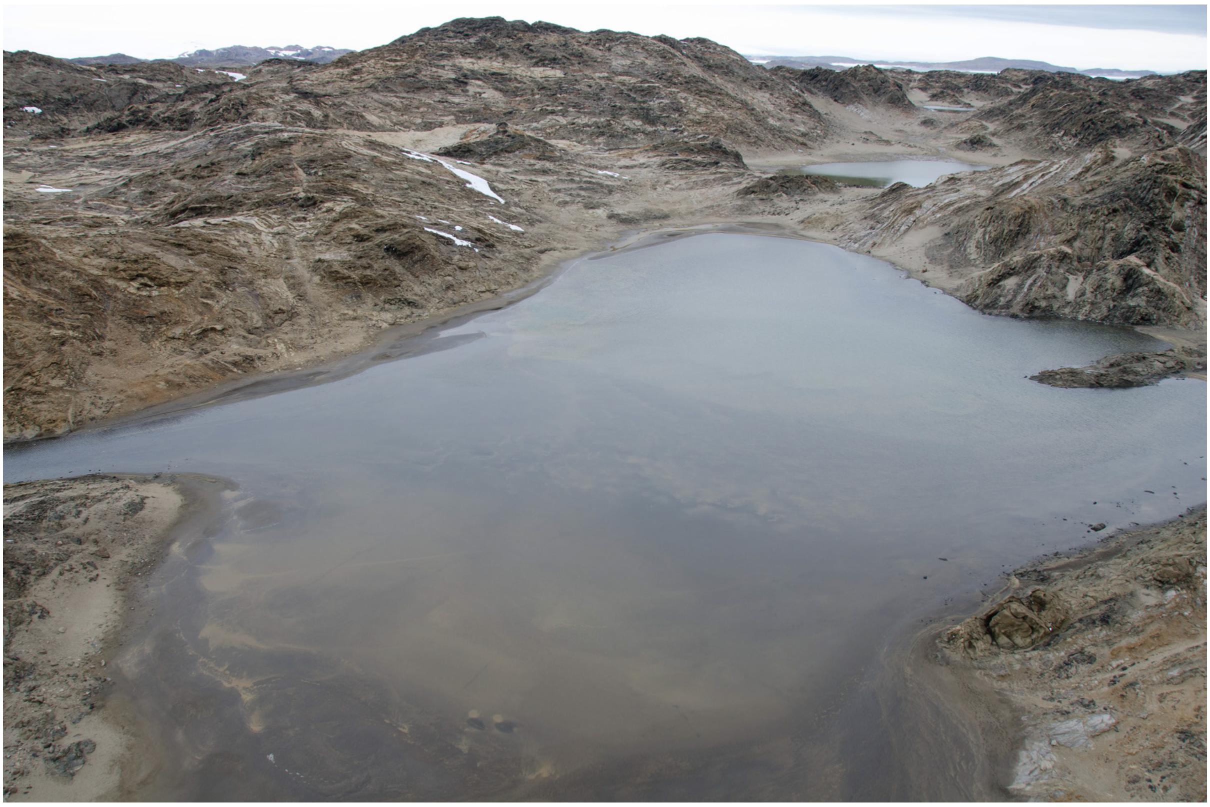

<u>Rauer Islands: Rauer 3 Lake (Filla Island)</u> (photo credits: Alyce Hancock)

GPS Co-ordinates: S68˚48.88’, E077˚49.64’

Elevation: 4m

Date: 11/01/2015

Time: 2.15pm

Weather: Cloudy, slight wind (less than 5 knots), warm ~+2˚C

Water Temperature: 10.5 degrees (thermometer)

Approximate Depth of Water at Sampling Site: less than 30min deep

Approximate Volume Filter: 50L filtered at 2pm on 12/01/2015 for 35min

Sediment type (bottom of lake): sandy/silty

Sediment type (edge of the lake): rocky/sandy

Observations: No ice on the lake or snow around the lake. Water clear.

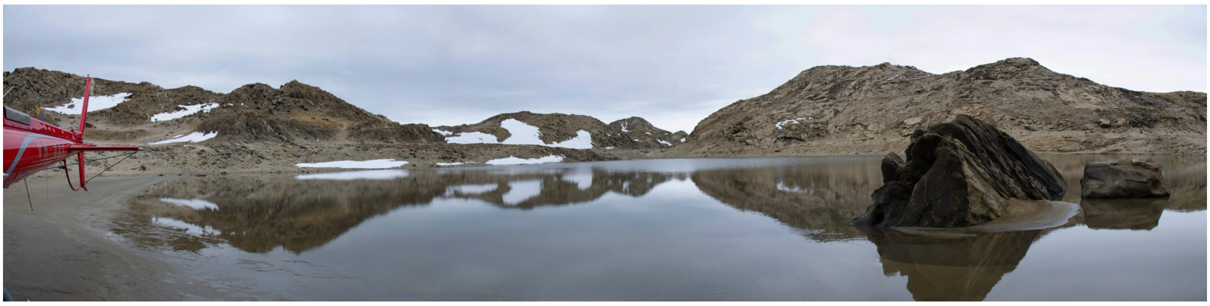

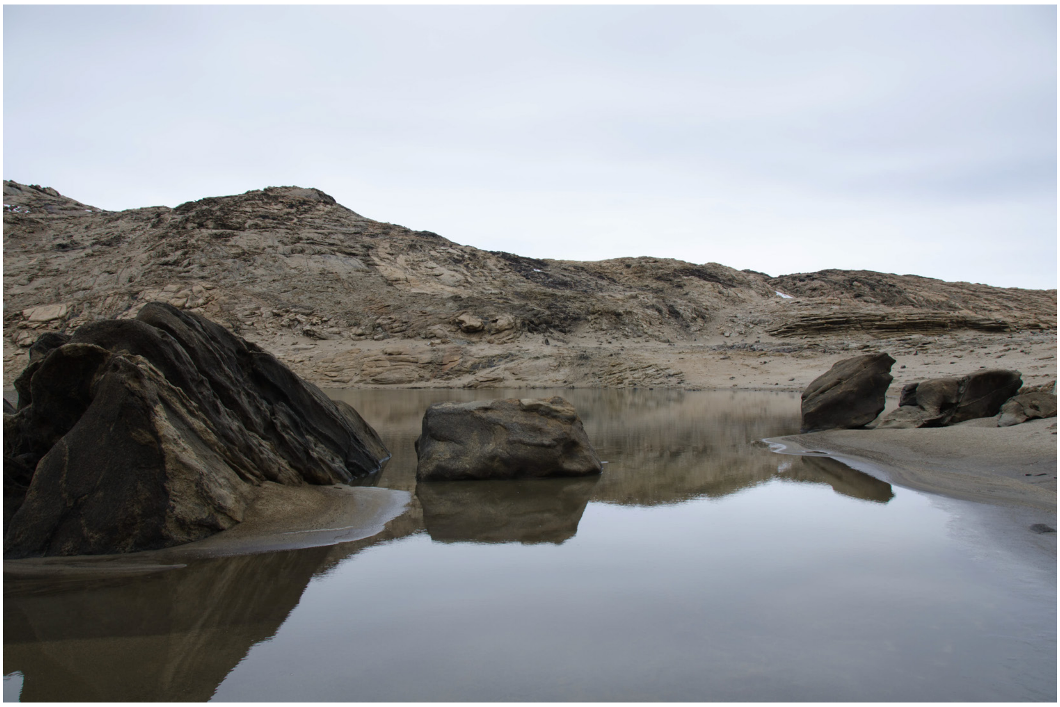

<u>Rauer Islands: Rauer 6 Lake (Torckler Island)</u> (photo credits: Sarah Payne)

GPS Co-ordinates: S68˚53.27’, E077˚50.37’

Date: 11/1/2015

Time: 1pm

Weather: Cloudy, wind <5kn, Temp ~0-2˚C

Water Temperature: 9˚C

Approximate Depth of Water at Sampling Site: 10-15cm

Approximate Volume Filter: 50L (Filtered at 4.30pm on 12/1/2015)

Sediment type (bottom of lake): Bottom sand/silt covered in a thick salt crystal crust

Sediment type (edge of the lake): Wide open edges of large flat rock

Observations: The lake was very shallow <30cm. There was a crust of salt crystals on the sediment (which make the lake look frozen from the air).

There also appeared to be stratification in the lake with a clear layer of ~10cm on the top and another layer of ~5cm underneath on the bottom. These layers weren’t visible until they were disturbed and a visible haze produced when these layers were mixed.

The sample was taken using a jug taking water mainly from the top layer but trying to disturb the layers so that some water from the bottom layer was also collected. It is likely the sample is mainly water from the top layer with some bottom layer water.

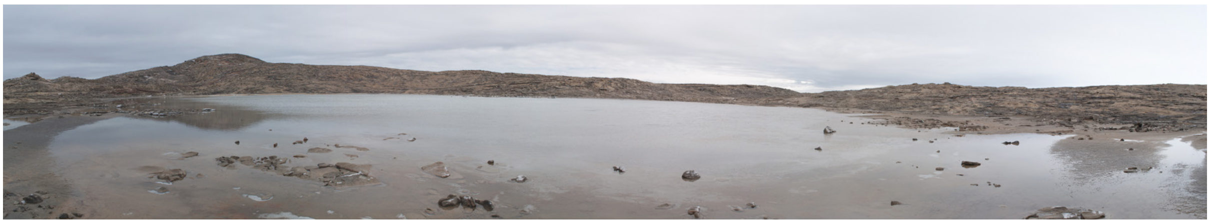

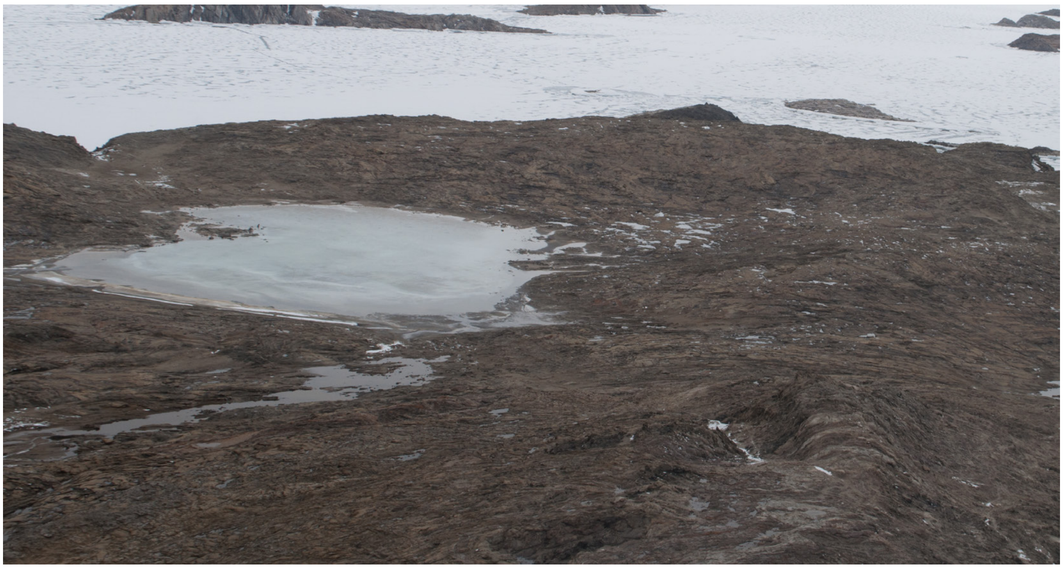

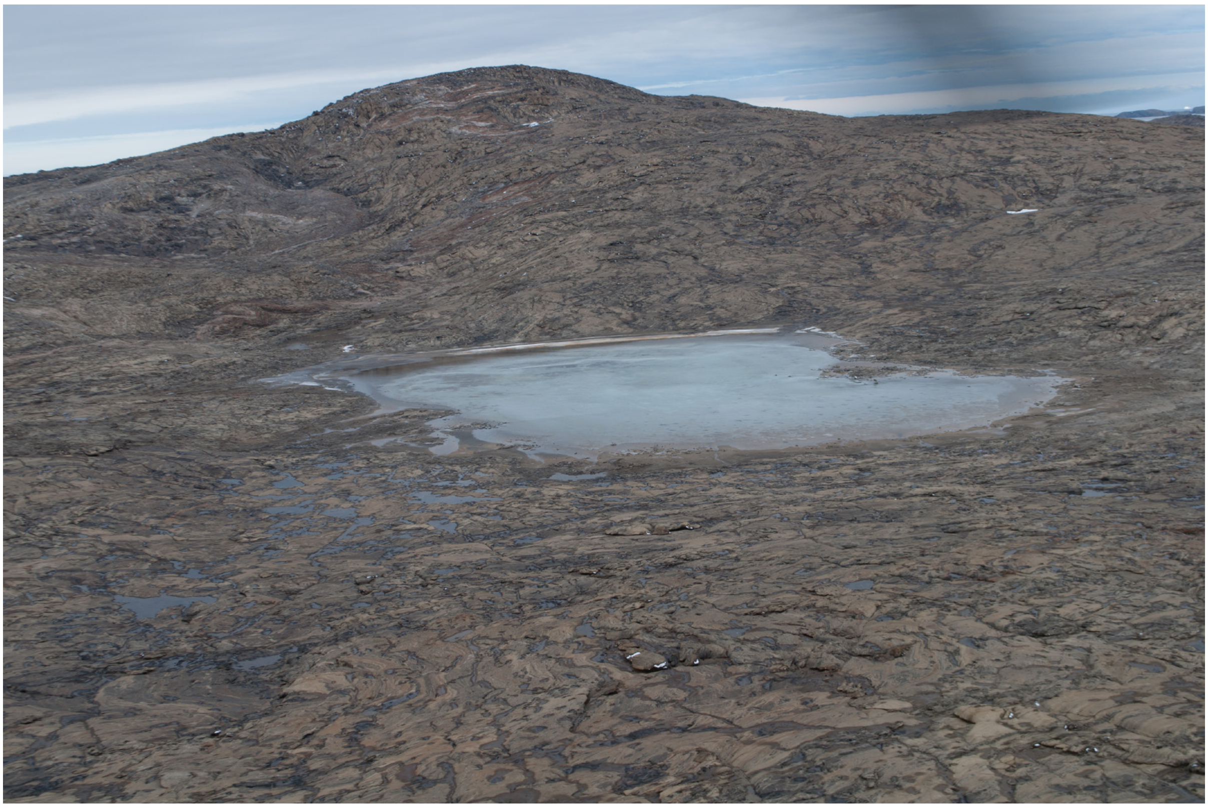

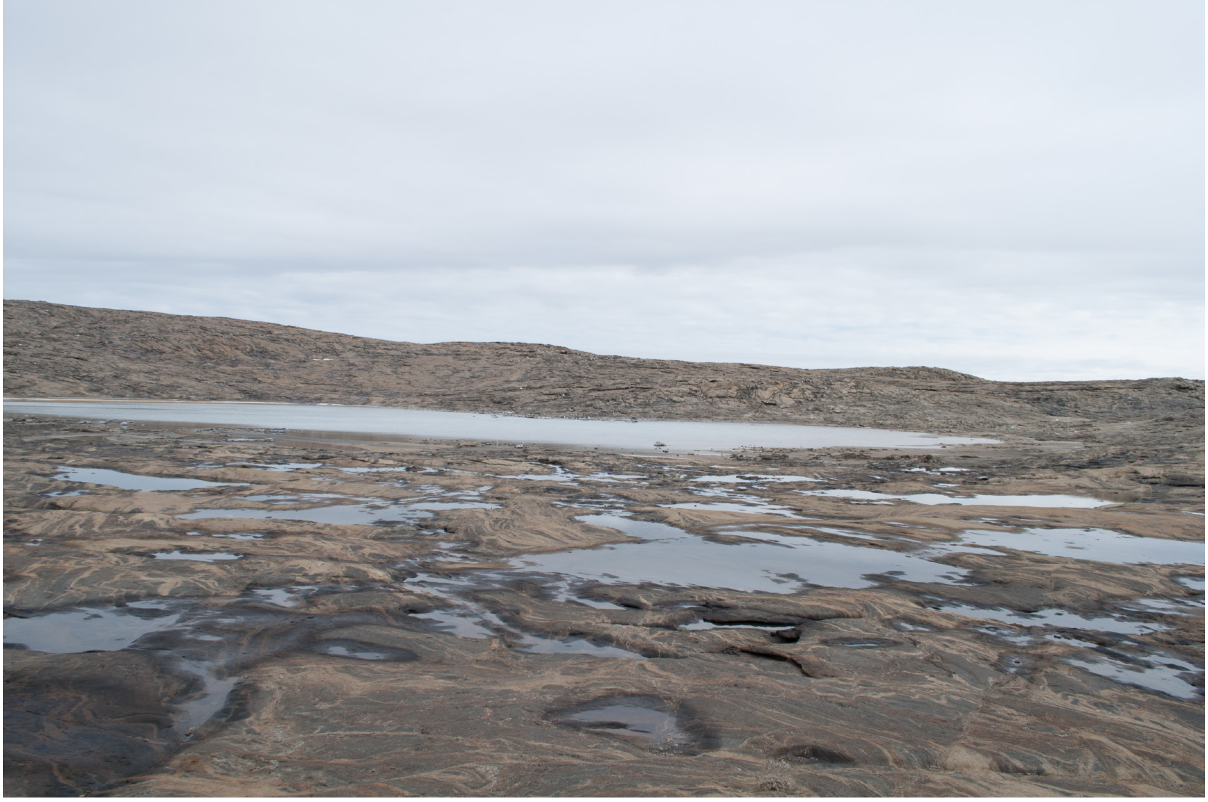

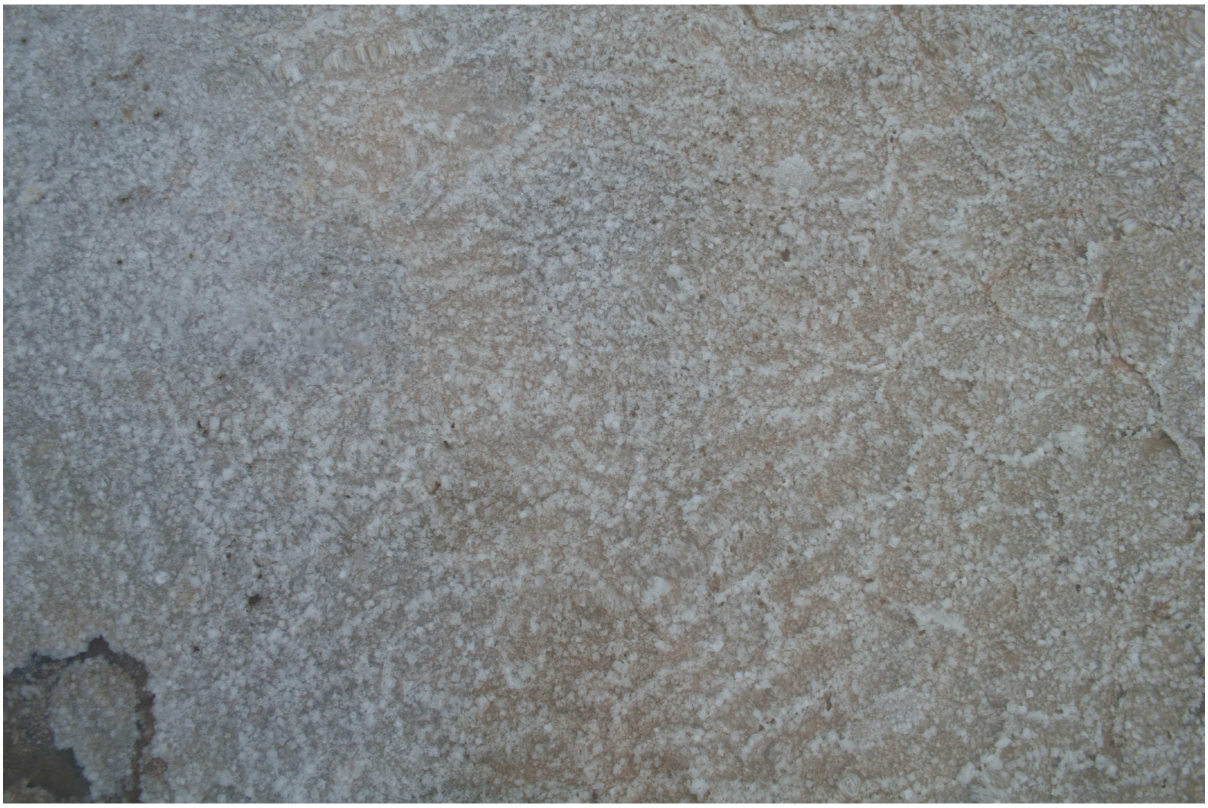

Salt crystals on the bottom of the lake

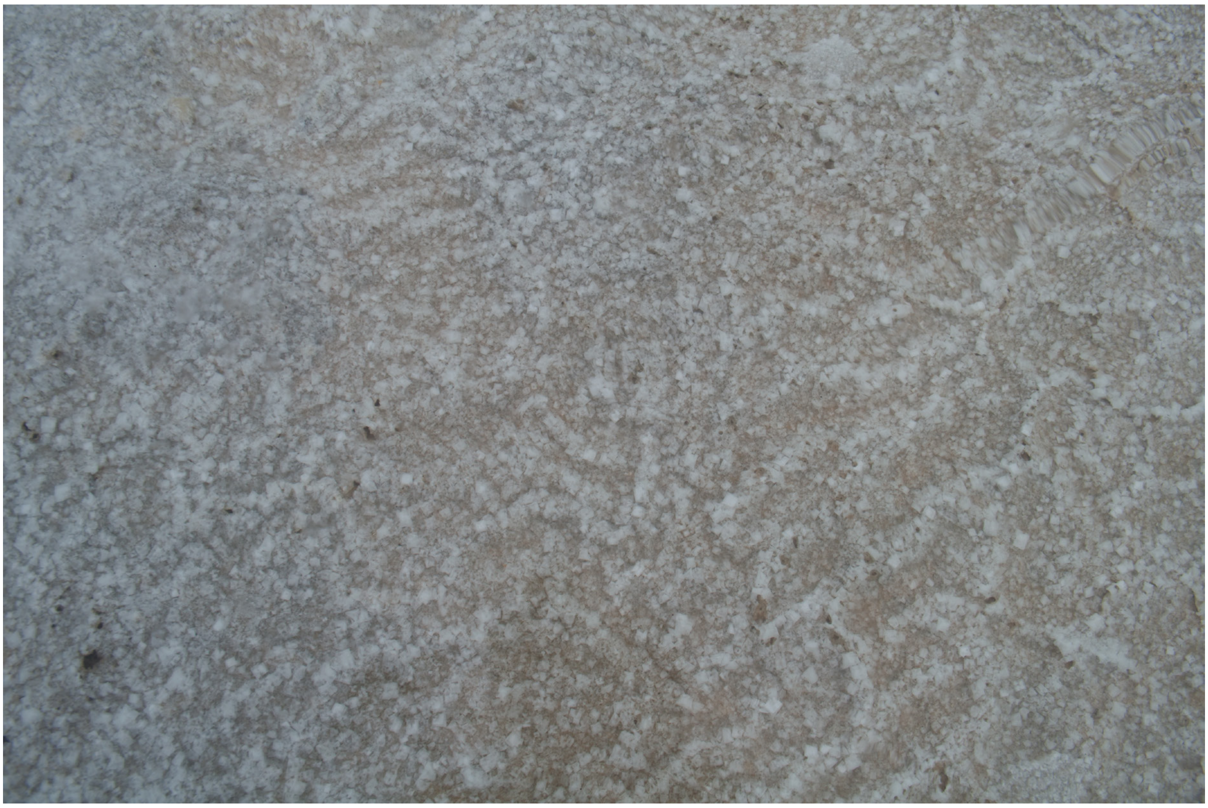

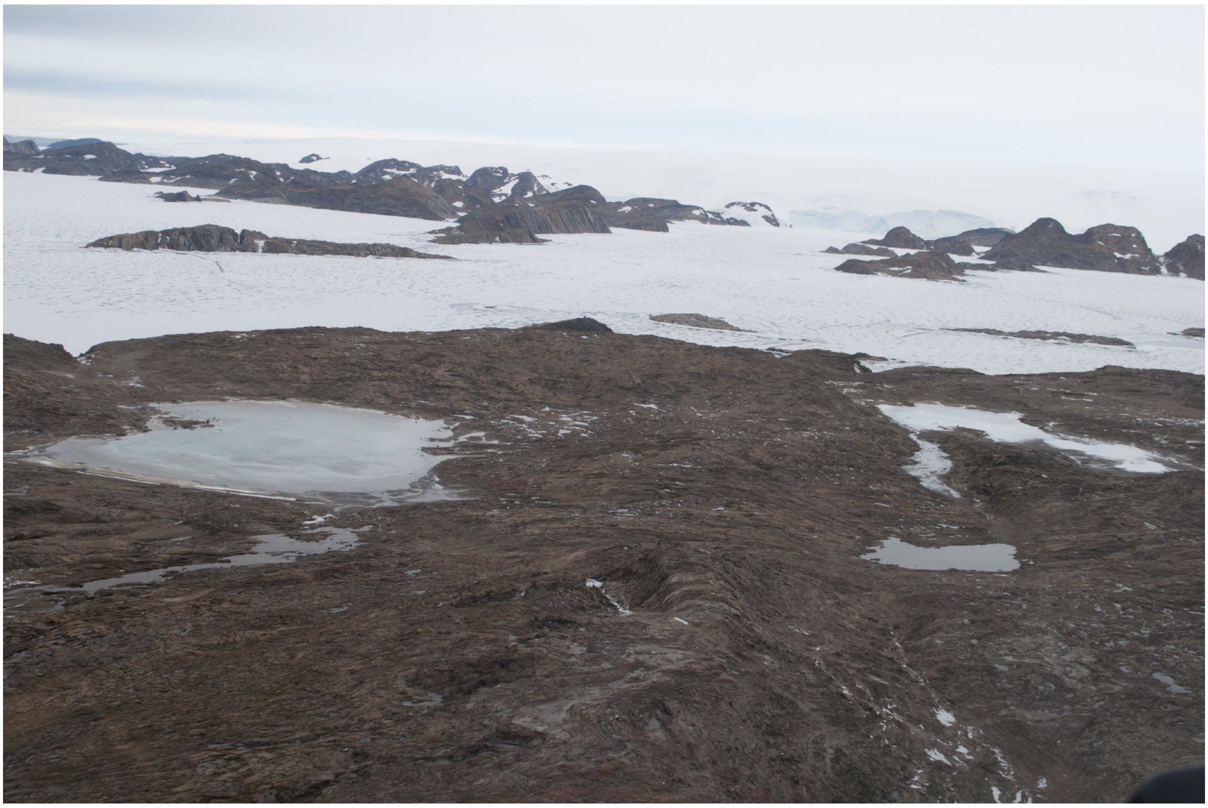

Torckler Island – Rauer 6 Lake (left) and Rauer 13 Lake (right)

<u>Rauer Islands: Rauer 13 Lake (Torckler Island)</u> (photo credits: Sarah Payne)

GPS Co-ordinates: S68˚53.37’, E077˚50.69’

Date: 11/1/2015

Time: 1.20pm

Weather: Cloudy, wind <5kn, Temp ~0-2˚C

Water Temperature: 9 ˚C

Approximate Depth of Water at Sampling Site: 10-15cm

Approximate Volume Filter: 50L filtered at 4.40pm on 12/1/2015

Sediment type (bottom of lake): Bottom had a sandy base but was totally covered in a slushy salt layer. This salt crystal slush sat on the bottom of the lake covering the sediment across the whole lake.

Sediment type (edge of the lake): The lake was high on the top of Torckler Island surrounded by open flat rocks.

Observations: This was a very open shallow lake (<30cm). Salt crystals also building up on the rocks around the lake where water is splashed from the lake. The sample was taken using a jug from a rock at the edge of the lake. Water was collected from the surface trying to get water from all depths but care was taken to get close to the bottom but not disturb the salt layer at the bottom.

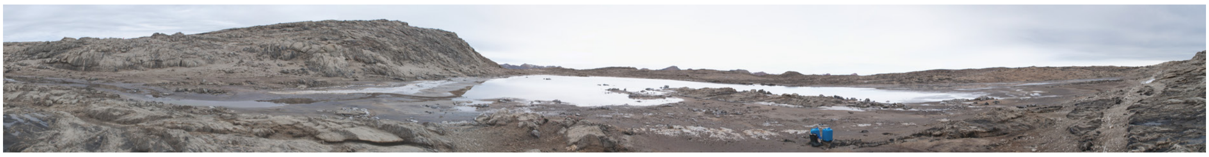

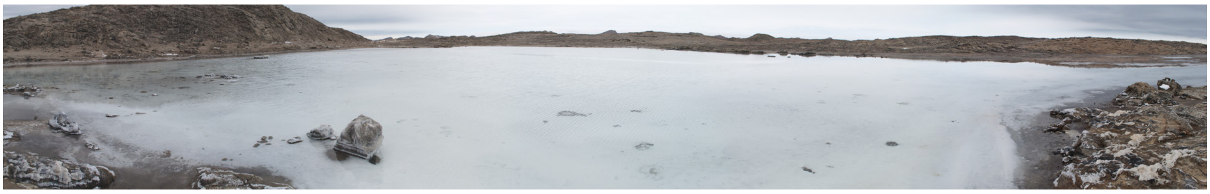

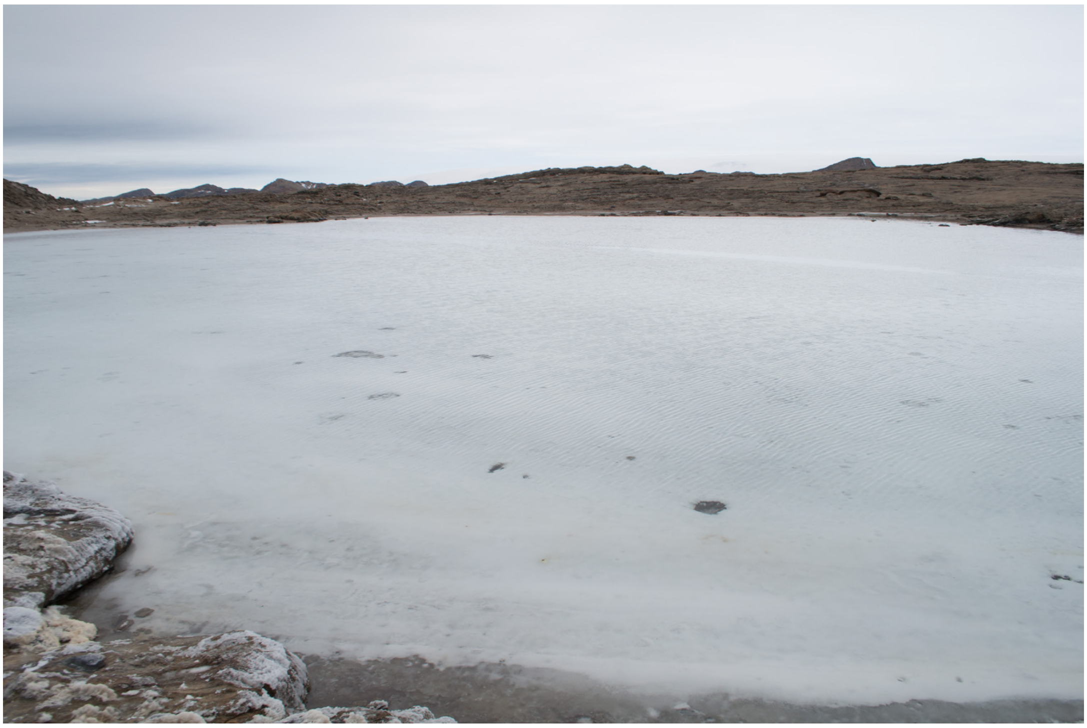

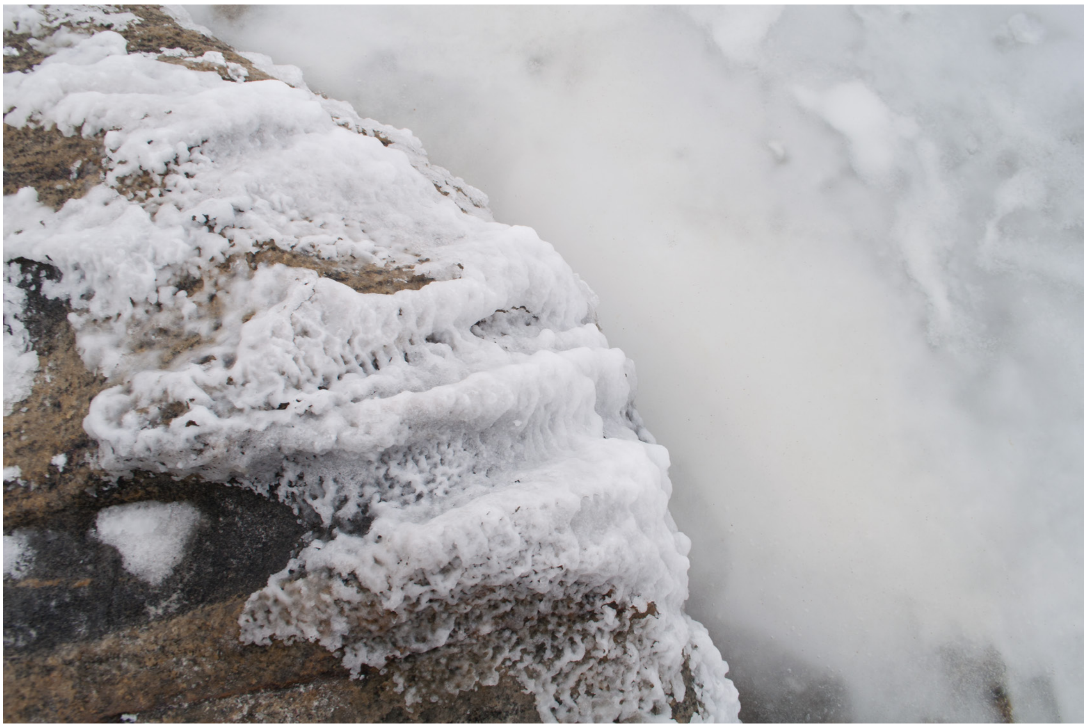

Salt on the rocks at the edge of the lake.

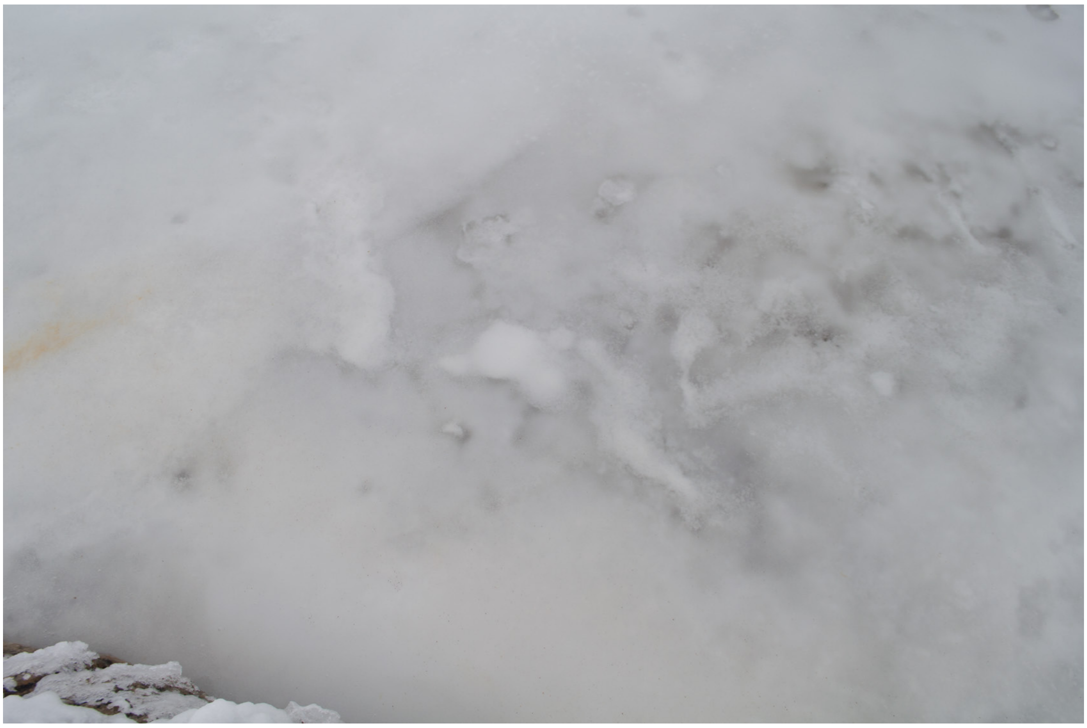
Salt slurry at the bottom on the lake

<u>Vestfold Hills: Club Lake</u> (photo credits: Sarah Payne)

Date: 26/11/2014

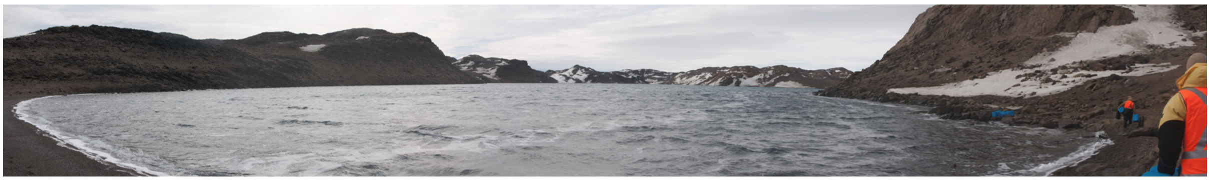

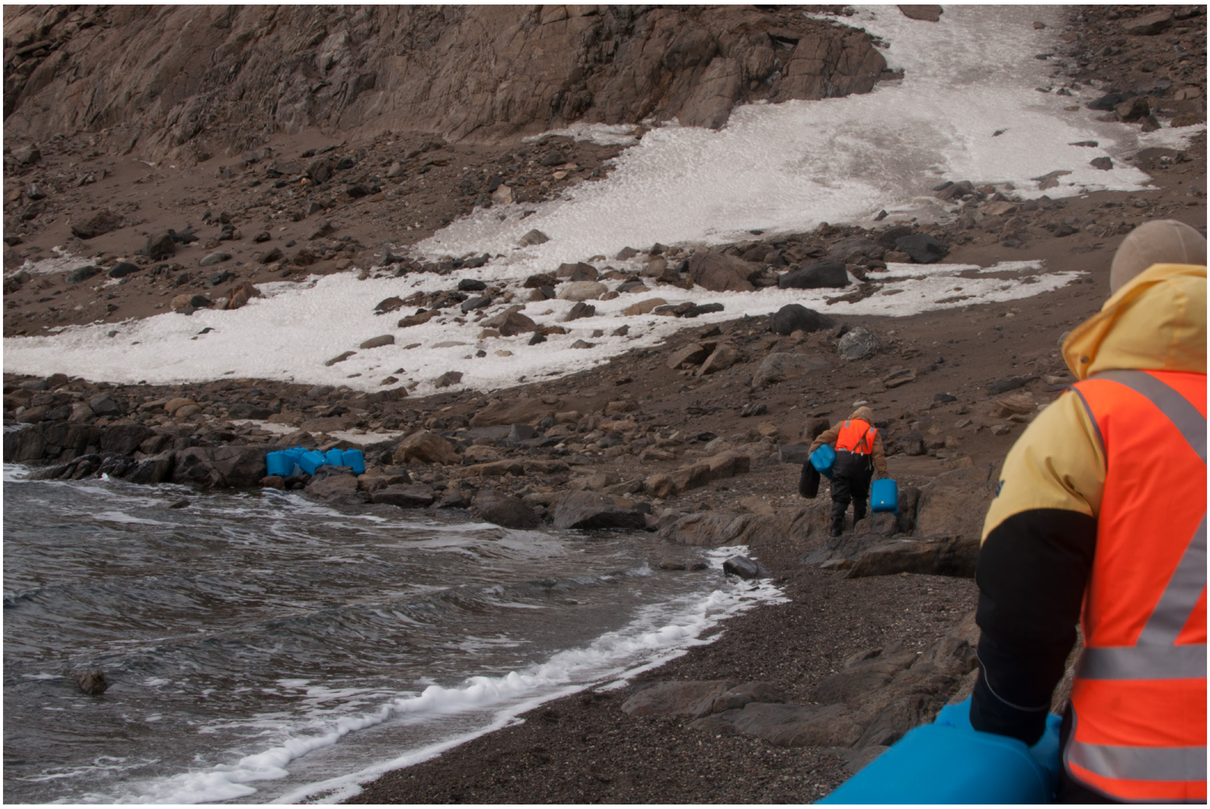

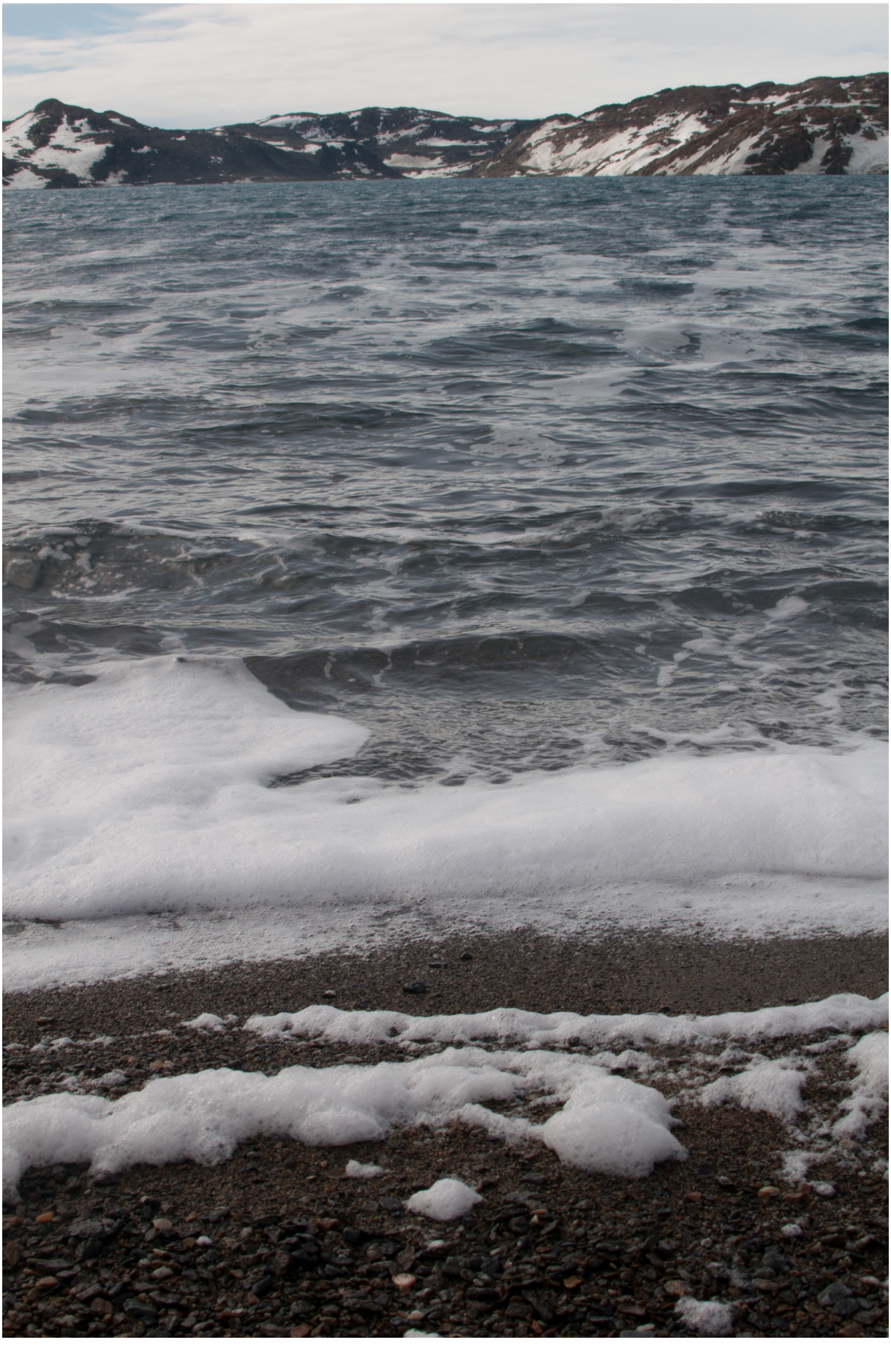

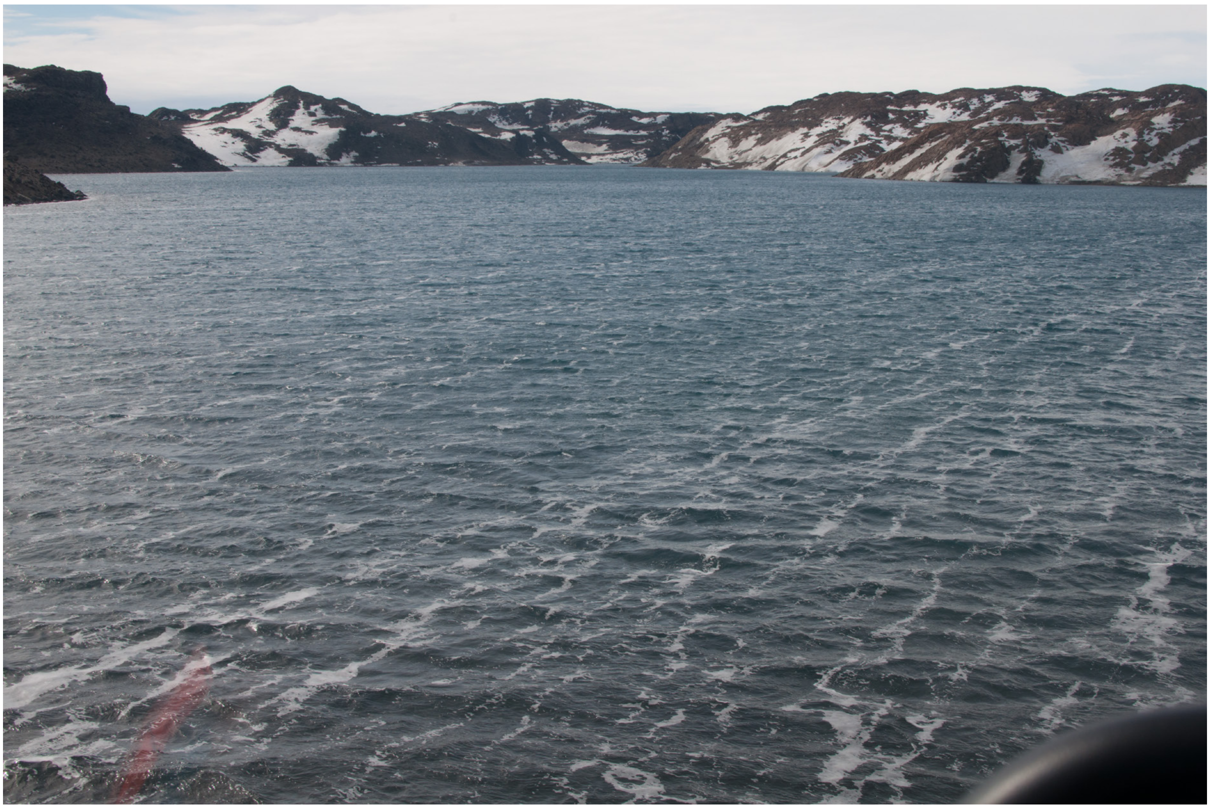

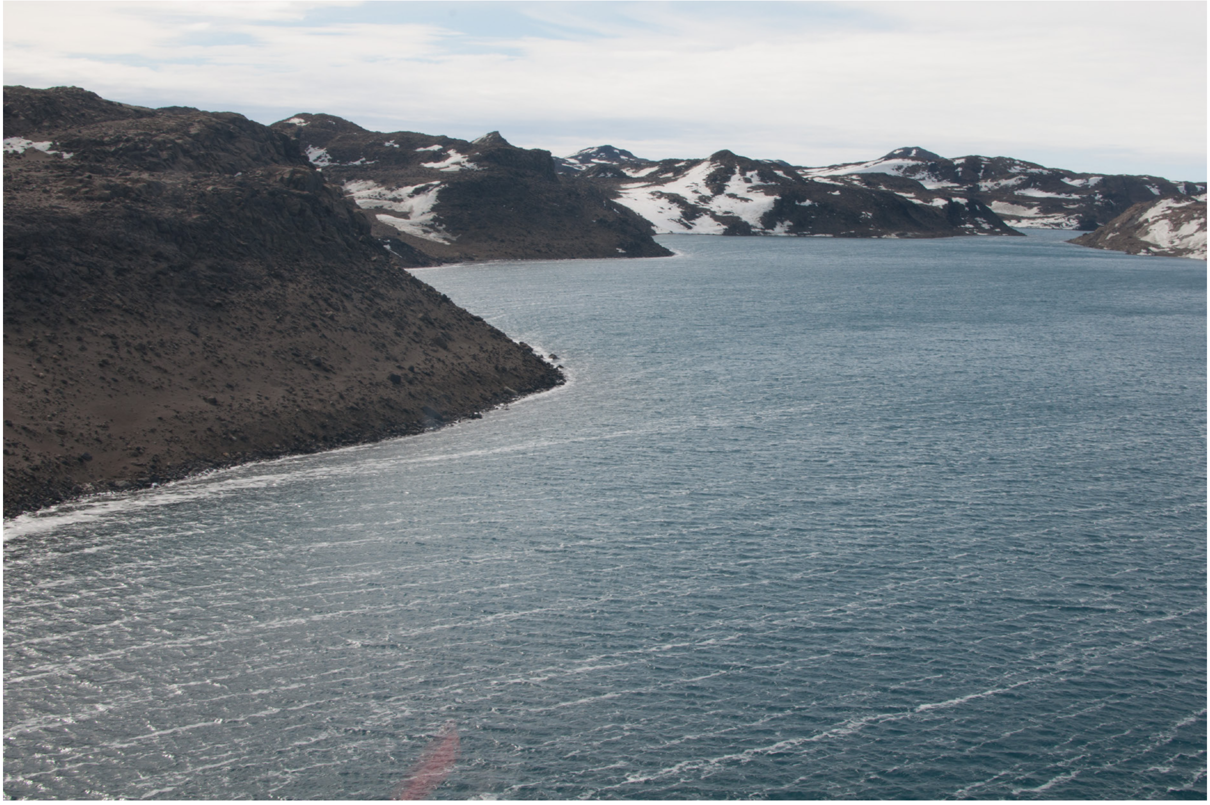

<u>Vestfold Hills: Deep Lake</u>

Date: 13/12/2013

(photo credits: Sarah Payne)

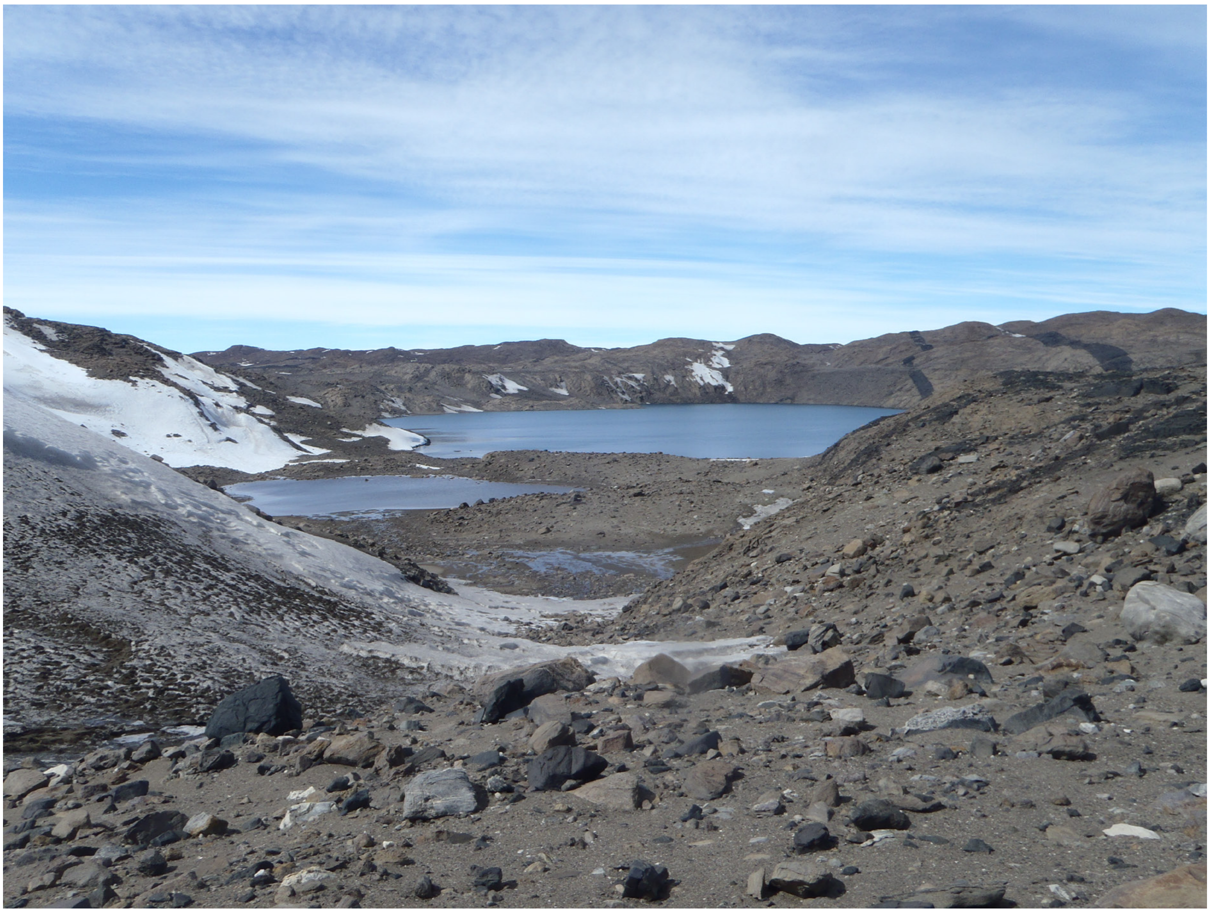

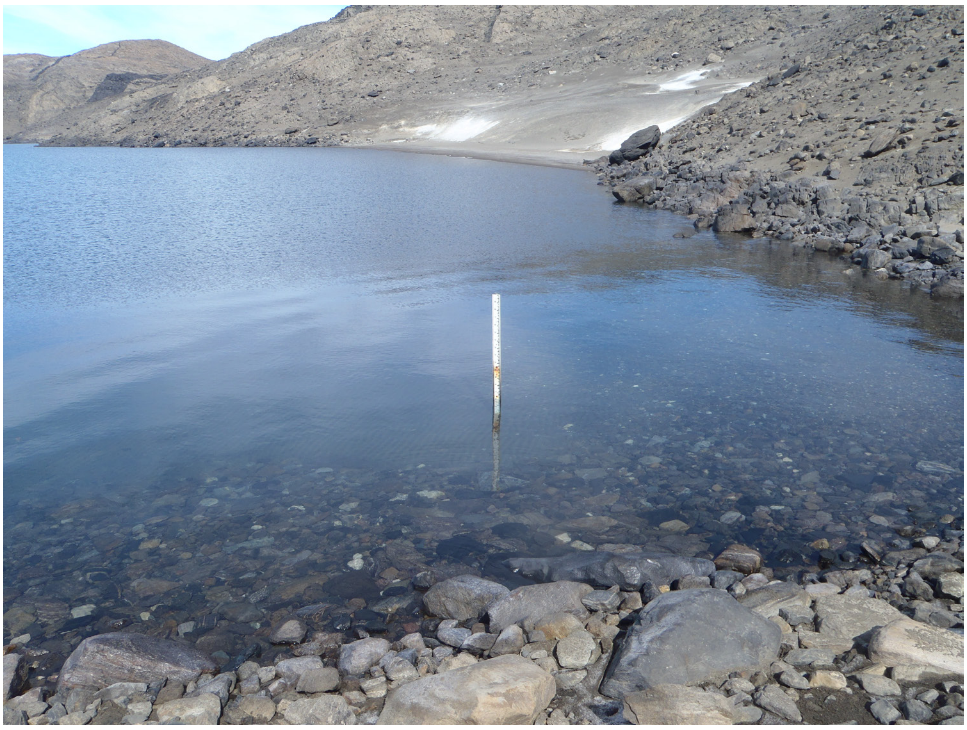

(photo credits: Alyce Hancock)

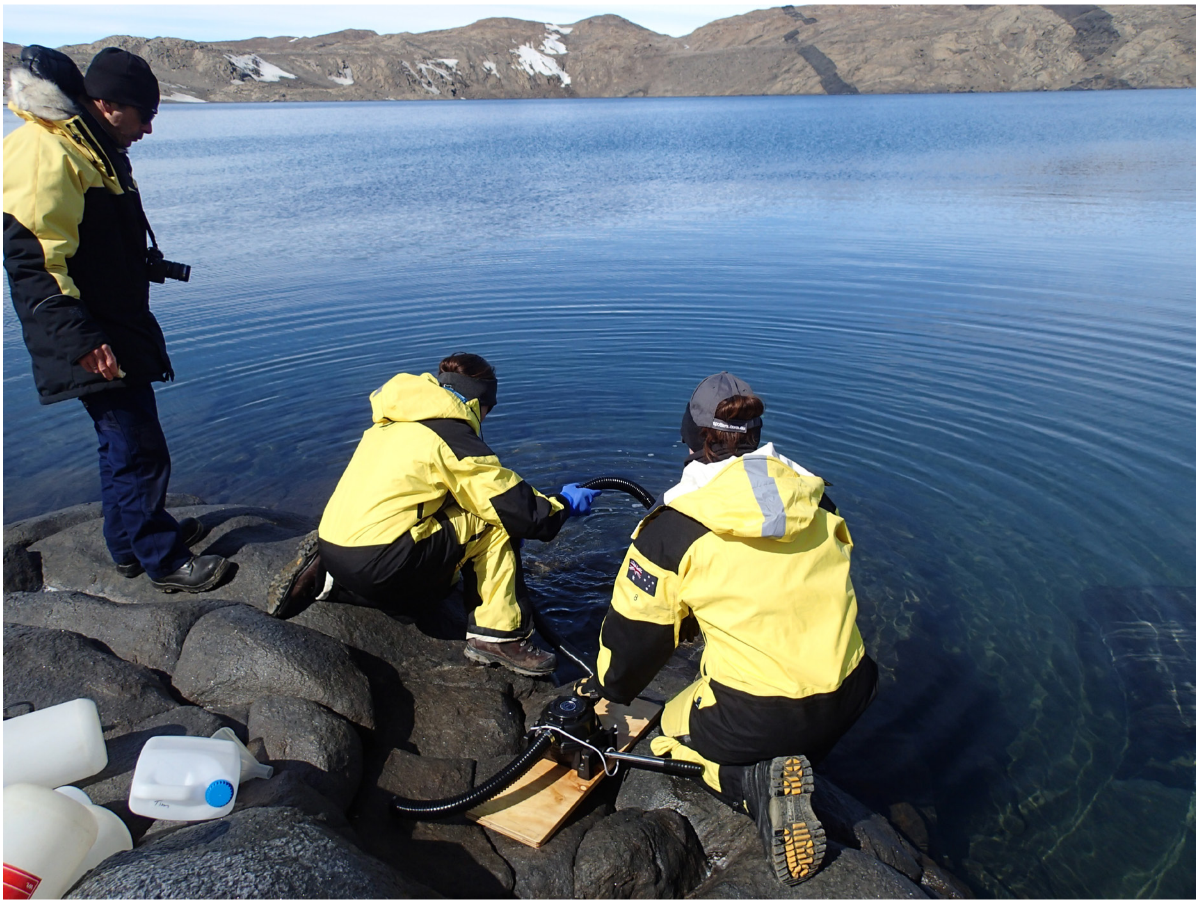

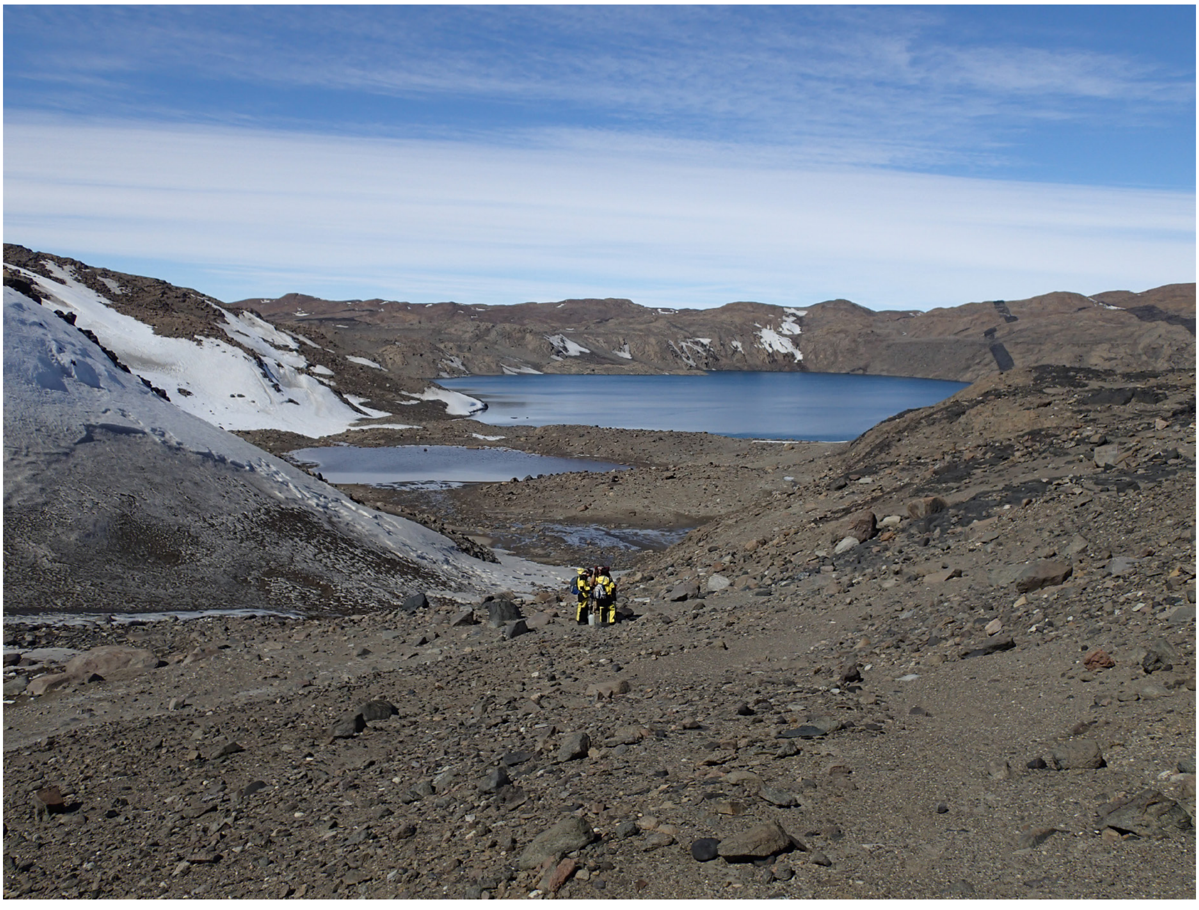

Deep Lake: 12/06/2014

(photo credits: Alyce Hancock)

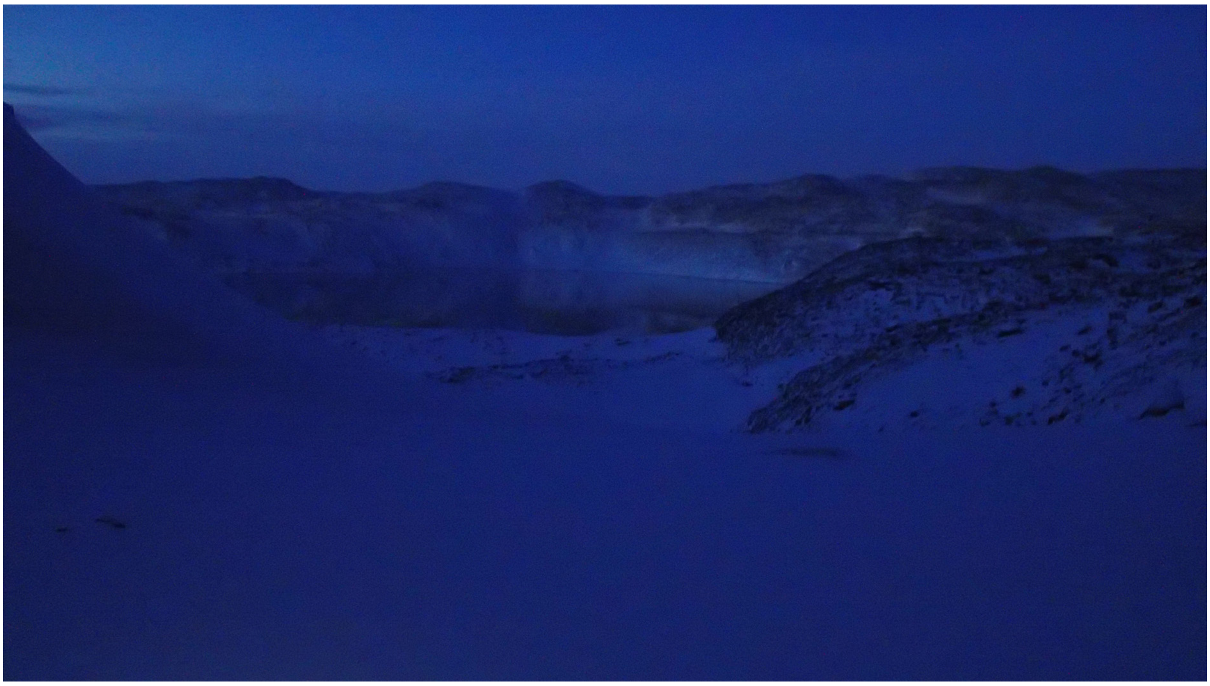

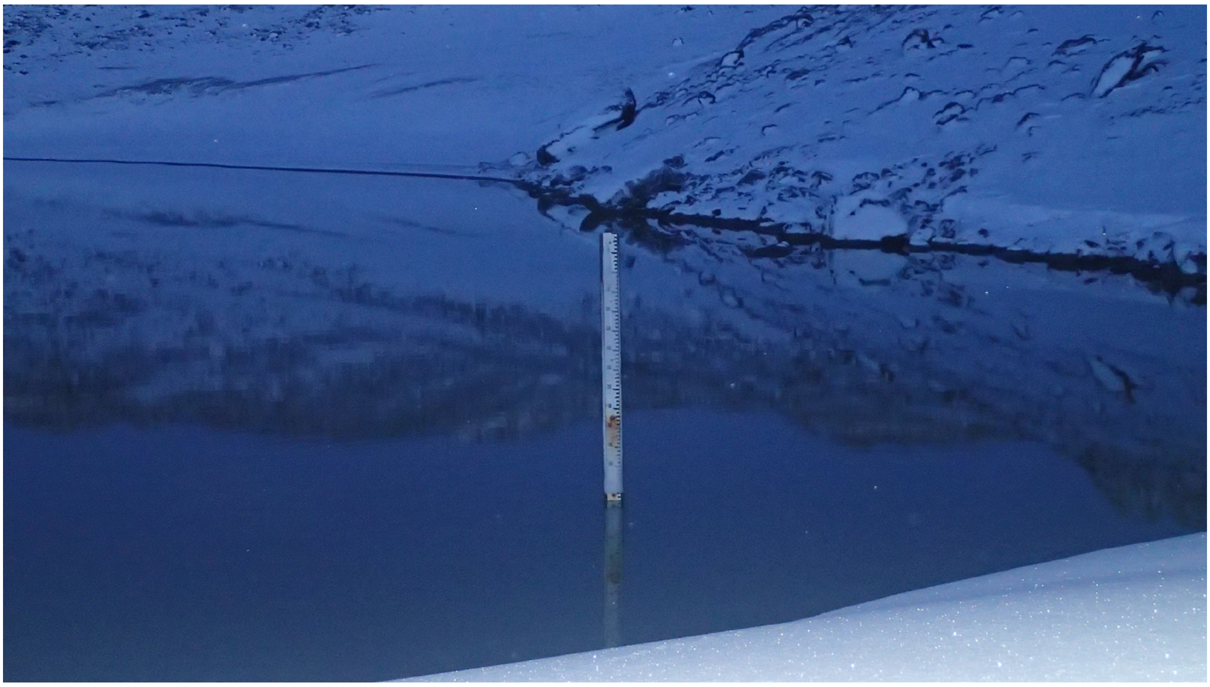

Deep Lake: 18/12/2014 (photo credits: Alyce Hancock)

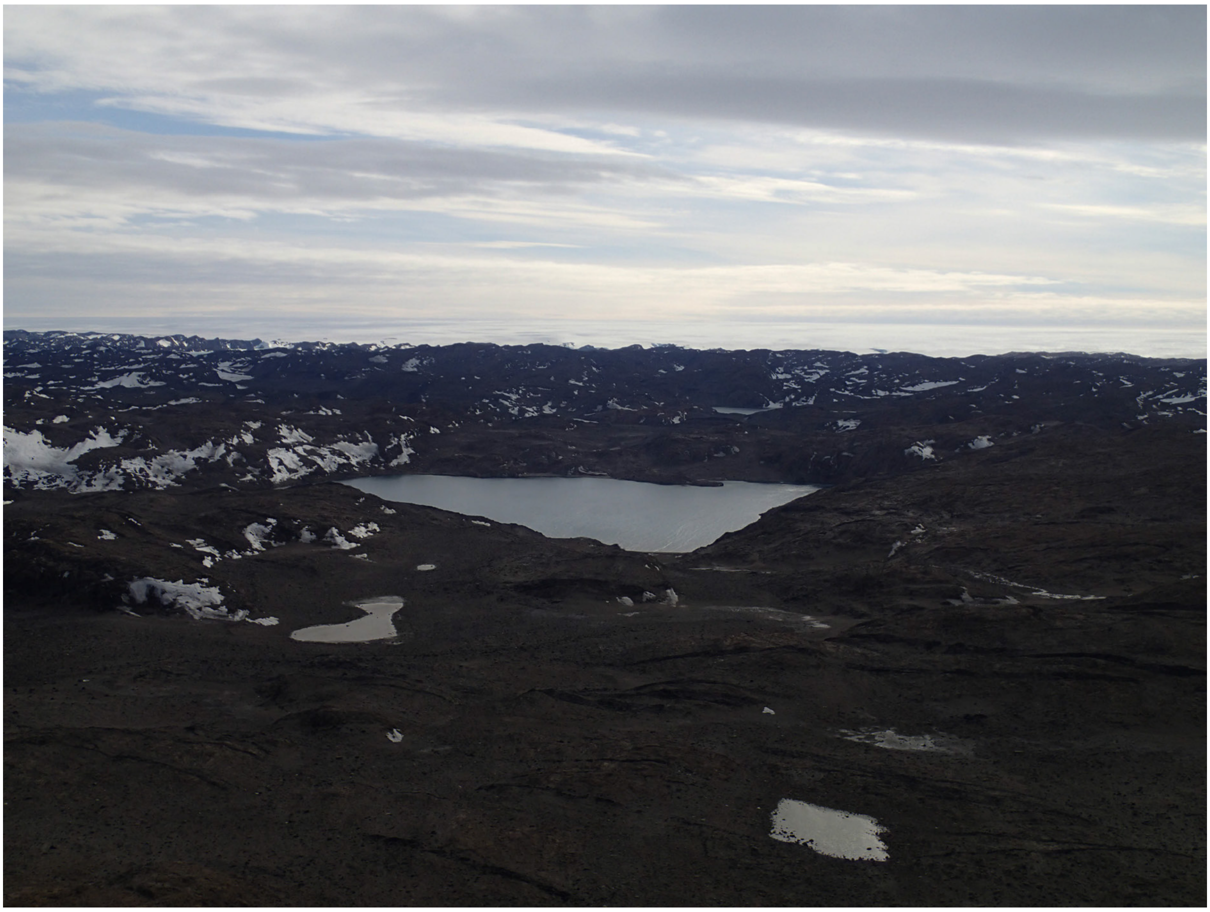

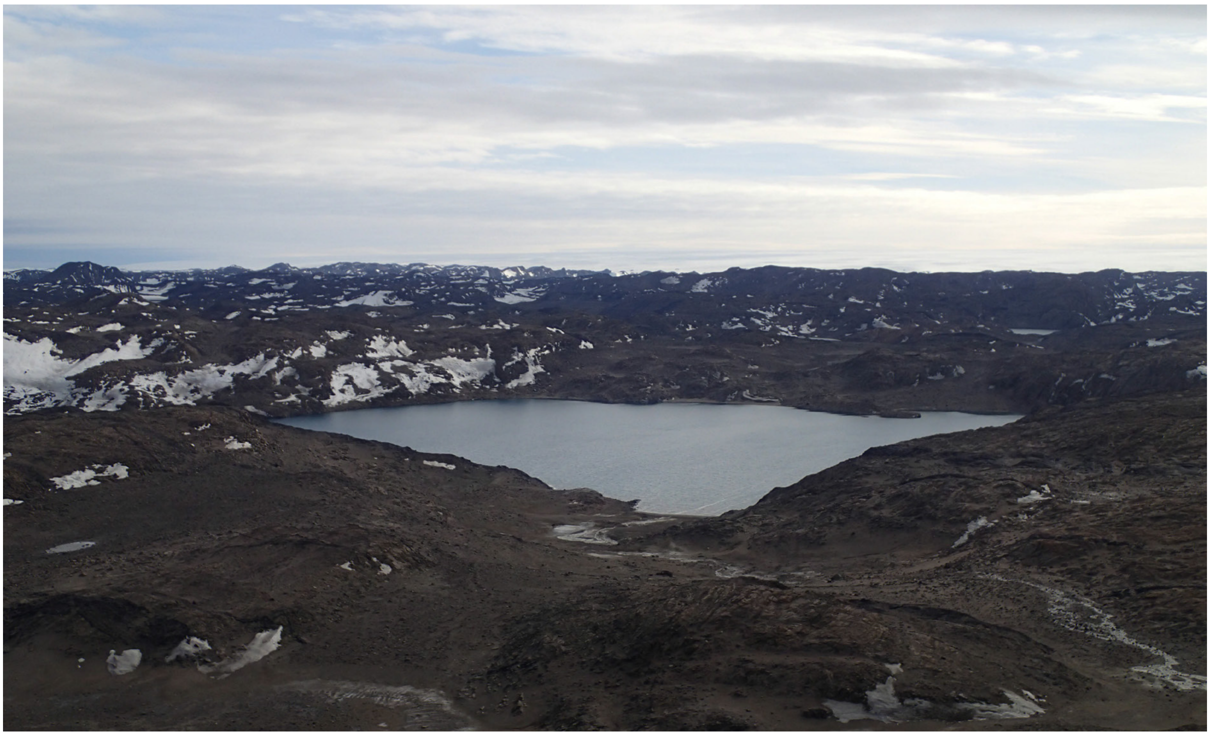

Supplement: Supplementary file 1 — Supplementary results: Sampling during the 2013–2015 season. (PDF 13433 kb) [file 40168_2018_495_MOESM1_ESM.pdf]
