# Supplementary material for: Genomic variation and biogeography of Antarctic haloarchaea
Source: Microbiome. 2018 Jun 20;6:113. doi: 10.1186/s40168-018-0495-3 (PMC6011602; doi:10.1186/s40168-018-0495-3)
Supplement: Supplementary file 2 — Figures S1-S8 and Tables S1-S13: Figure S1. Archaellin protein sequence alignment. Figure S2. arCOG functional classes of genes present on ACAM34 and R1S1 secondary replicons. Figure S3. arCOG functional classes of the genes within HIRs specific to R1S1 and DL1, DL31 and Hht. litchfieldiae tADL. Figure S4. New HIRs present in R1S1 that are shared with Hht. litchfieldiae tADL. Figure S5 Genomic islands on primary replicons of Antarctic haloarchaea. Figure S6. Metagenome coverage and HCA of selected secondary replicons. Figure S7. Contigs assembled from metagenomes mapped to replicons. Figure S8. Clustering and scaling of samples. Table S1. Description of the Rauer Islands and Vestfold Hills hypersaline lakes sampled in this study. Table S2. Antarctic lake metagenomes used in this study. Table S3. Unique transposases of ACAM34 and R1S1. Table S4. Unique protein-coding genes. Table S5. Unique sequence duplications and non-coding RNAs on the R1S1 primary replicon. Table S6. Regions with low sequence similarity between ACAM34 and R1S1 primary replicons. Table S7. New HIRs identified in R1S1. Table S8. HIR conserved between Hrr. lacusprofundi R1S1 and DL1 that encodes a type I-B CRISPR system. Table S9. R1S1 CRISPR spacer matching Hlac-Pro1 in ACAM34. Table S10. Presence of 16S rRNA gene sequences for known Antarctic haloarchaeal species in Antarctic hypersaline lakes. Table S11. Relative abundance of Hrr. lacusprofundi ACAM34, Hht. litchfieldiae tADL, DL31 and DL1 in Antarctic lake metagenomes. Table S12. Relative abundance of lake taxa assessed from read coverage and taxonomic assignment of contigs assembled from metagenome data. Table S13. Genome coverage and percent identity for contigs mapped to replicons of Hrr. lacusprofundi R1S1 and ACAM34, Hht. litchfieldiae tADL, DL31 and DL1. (PDF 7786 kb) [file 40168_2018_495_MOESM2_ESM.pdf]

Additional file 2

Genomic variation and biogeography of Antarctic haloarchaea

Bernhard Tschitschko, Susanne Erdmann, Matthew Z. DeMaere, Simon Roux, Pratibha Panwar, Michelle A. Allen, Timothy J. Williams, Sarah Brazendale, Alyce M. Hancock, Nicole Shapiro, Emiley A. Eloe-Fadrosh and Ricardo Cavicchioli

Supplementary Figures

Figure S1 Archaellin protein sequence alignment.
Figure S2 arCOG functional classes of genes present on ACAM34 and R1S1 secondary replicons.
Figure S3 arCOG functional classes of the genes within HIRs specific to R1S1 and DL1, DL31 and *Hht. litchfieldiae* tADL.
Figure S4 New HIRs present in R1S1 that are shared with *Hht. litchfieldiae* tADL.
Figure S5 Genomic islands on primary replicons of Antarctic haloarchaea.
Figure S6 Metagenome coverage and HCA of selected secondary replicons.
Figure S7 Contigs assembled from metagenomes mapped to replicons.
Figure S8 Clustering and scaling of samples.

Supplementary Tables

Table S1 Description of the Rauer Islands and Vestfold Hills hypersaline lakes sampled in this study.
Table S2 Antarctic lake metagenomes used in this study.
Table S3 Unique transposases of ACAM34 and R1S1.
Table S4 Unique protein-coding genes.
Table S5 Unique sequence duplications and non-coding RNAs on the R1S1 primary replicon.
Table S6 Regions with low sequence similarity between ACAM34 and R1S1 primary replicons.
Table S7 New HIRs identified in R1S1.
Table S8 HIR conserved between *Hrr. lacusprofundi* R1S1 and DL1 that encodes a type I-B CRISPR system.
Table S9 R1S1 CRISPR spacer matching Hlac-Pro1 in ACAM34.
Table S10 Presence of 16S rRNA gene sequences for known Antarctic haloarchaeal species in Antarctic hypersaline lakes.
Table S11 Relative abundance of *Hrr. lacusprofundi* ACAM34, *Hht. litchfieldiae* tADL, DL31 and DL1 in Antarctic lake metagenomes.
Table S12 Relative abundance of lake taxa assessed from read coverage and taxonomic assignment of contigs assembled from metagenome data.
Table S13 Genome coverage and percent identity for contigs mapped to replicons of *Hrr. lacusprofundi* R1S1 and ACAM34, *Hht. litchfieldiae* tADL, DL31 and DL1.

References

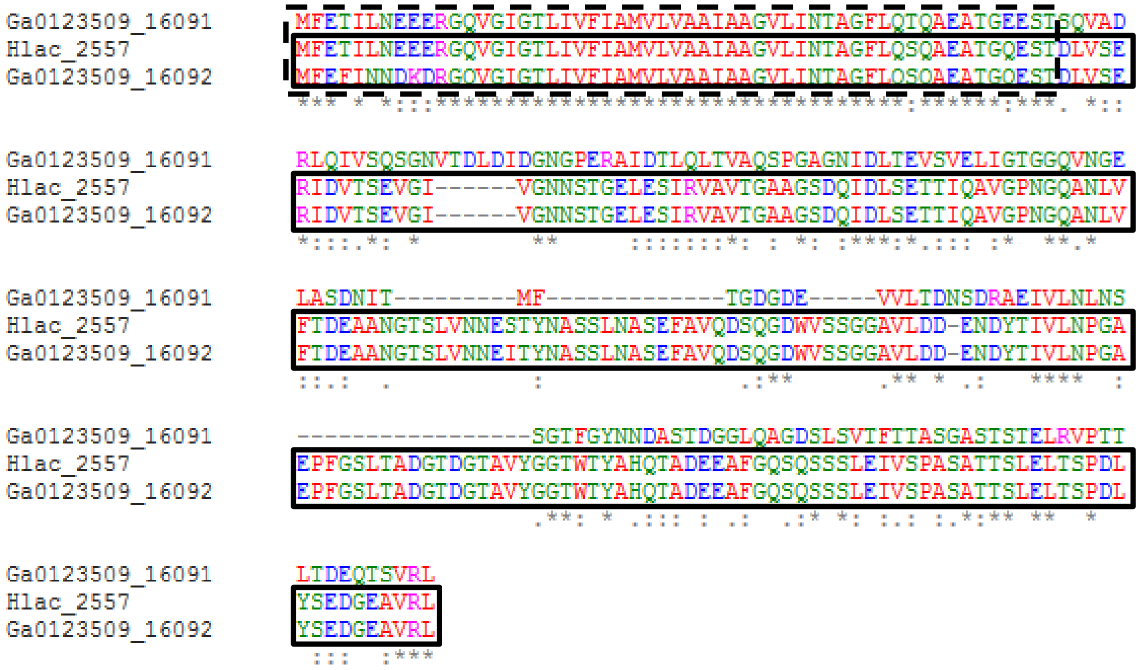

Figure S1 Archaellin protein sequence alignment. Multiple amino acid sequence alignment of the two R1S1 (Ga0123509_16091/16092) and the single ACAM34 (Hlac_2557) archaellin proteins. Regions of high sequence similarity between all three genes (black dashed line) and between Hlac_2557 and Ga0123509_16092 (full black line) are highlighted. Alignments performed using Clustal Omega [1]: amino acids identical in all three sequences (*); strong functional similarity (:); weak functional similarity (.).

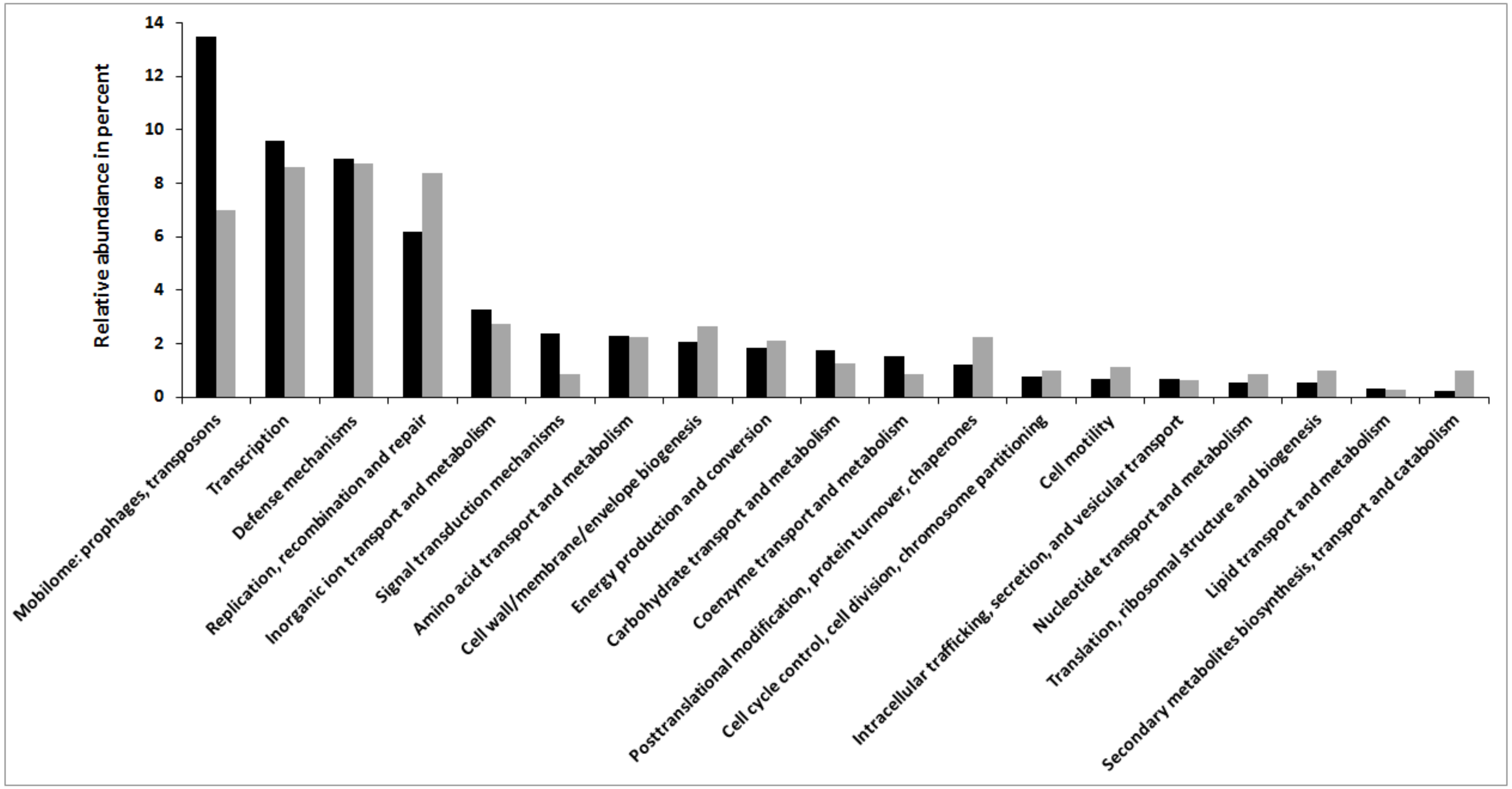

Figure S2 arCOG functional classes of genes present on ACAM34 and R1S1 secondary replicons. Relative abundance of genes assigned arCOG functional classes for ACAM34 (black) and R1S1 (grey). The representation of arCOG functional classes is broadly similar between the strains. The smaller proportion of genes belonging to the 'Mobilome: prophages, transposons' class in R1S1 is likely due to a lack of assembly of reads that match to transposase genes that are present with multiple copies. Not shown are genes assigned into 'General function prediction only' (ACAM34, 4% and R1S1, 5%), 'Function unknown' (ACAM34, 26; R1S1, 24%), and those not assigned any arCOG class (ACAM34, 12; R1S1, 17%).

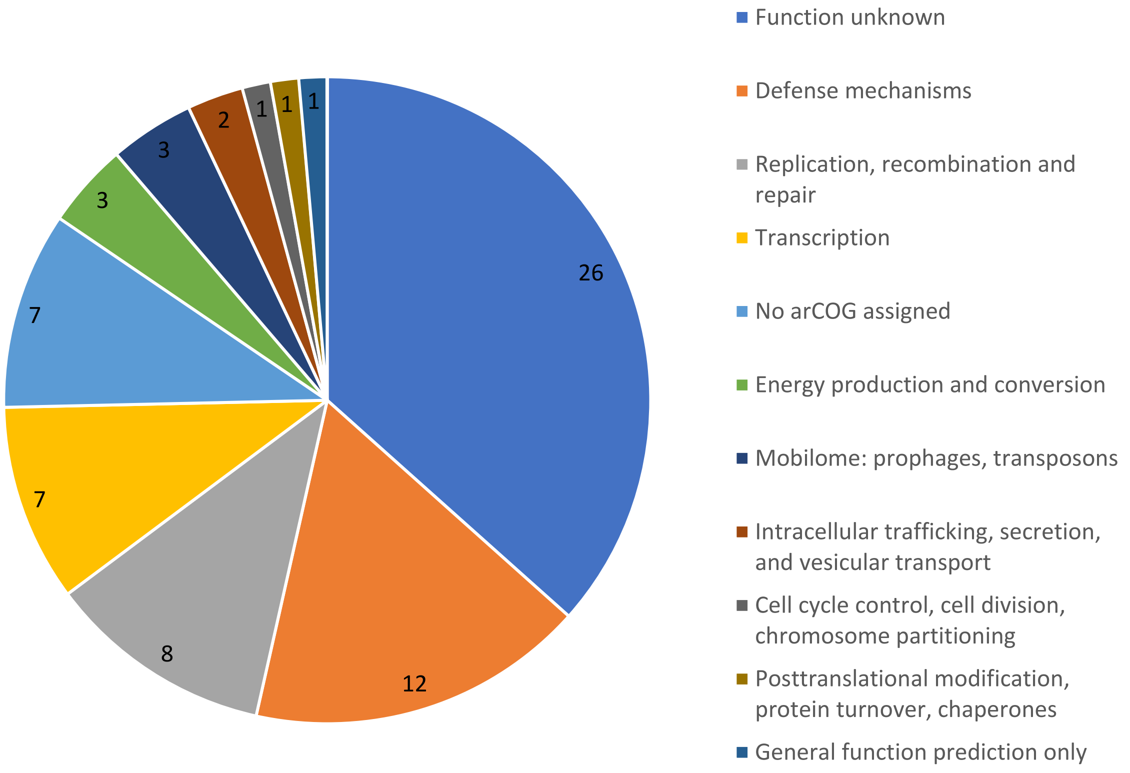

Figure S3 arCOG functional classes of the genes within HIRs specific to R1S1 and DL1, DL31 and *Hht. litchfieldiae* tADL. The eleven regions (2-14 kb in length) represent 67 kb of HIRs not present in ACAM34.

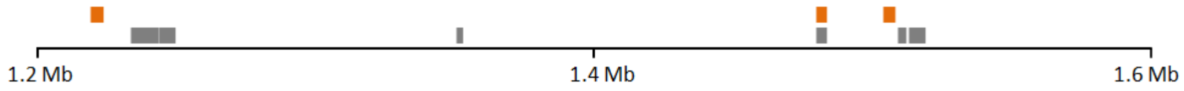

Figure S4 New HIRs present in R1S1 that are shared with *Hht. litchfieldiae* tADL. The figure shows a region of the *Hht. litchfieldiae* tADL replicon that contains new HIRs shared with R1S1 (orange blocks) that are in close proximity to previously described HIRs (grey blocks) [2].

a)

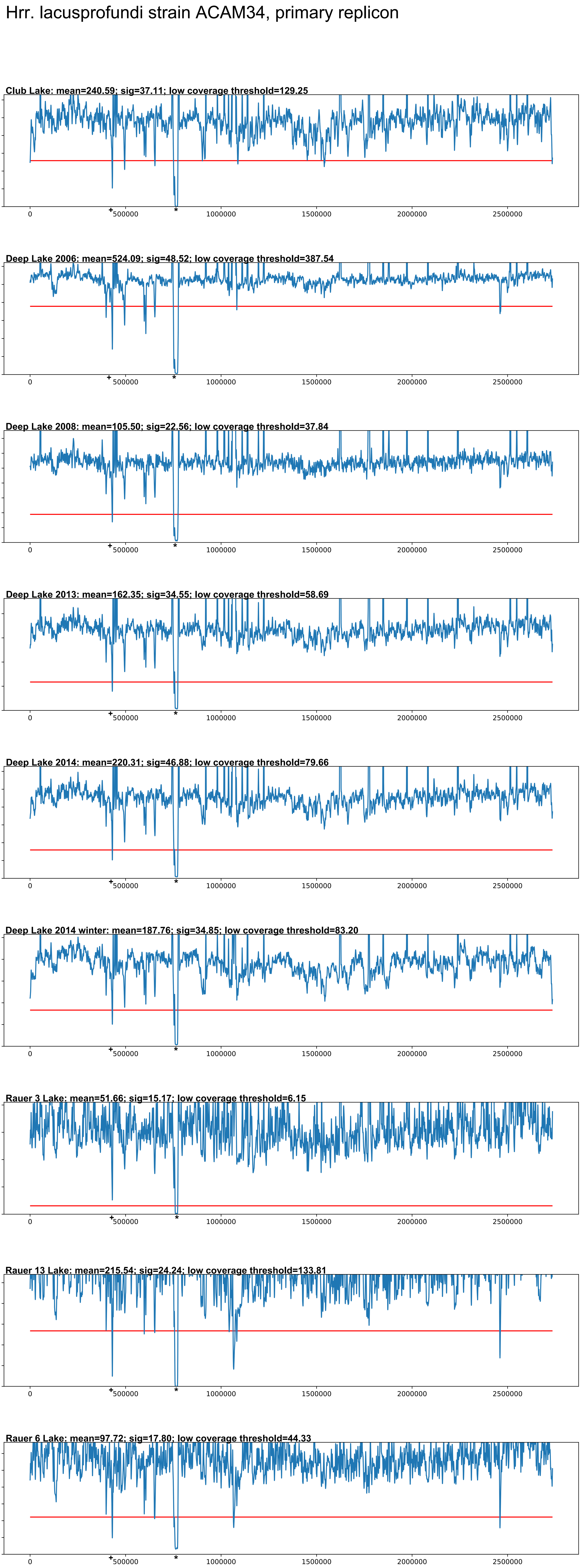
Hrr. lacusprofundi strain ACAM34, primary replicon
Club Lake: mean=240.59; sig=37.11; low coverage threshold=129.25
Deep Lake 2006: mean=524.09; sig=48.52; low coverage threshold=387.54
Deep Lake 2008: mean=105.50; sig=22.56; low coverage threshold=37.84
Deep Lake 2013: mean=162.35; sig=34.55; low coverage threshold=58.69
Deep Lake 2014: mean=220.31; sig=46.88; low coverage threshold=79.66
Deep Lake 2014 winter: mean=187.76; sig=34.85; low coverage threshold=83.20
Rauer 3 Lake: mean=51.66; sig=15.17; low coverage threshold=6.15
Rauer 13 Lake: mean=215.54; sig=24.24; low coverage threshold=133.81
Rauer 6 Lake: mean=97.72; sig=17.80; low coverage threshold=44.33

b)

DL31, primary replicon

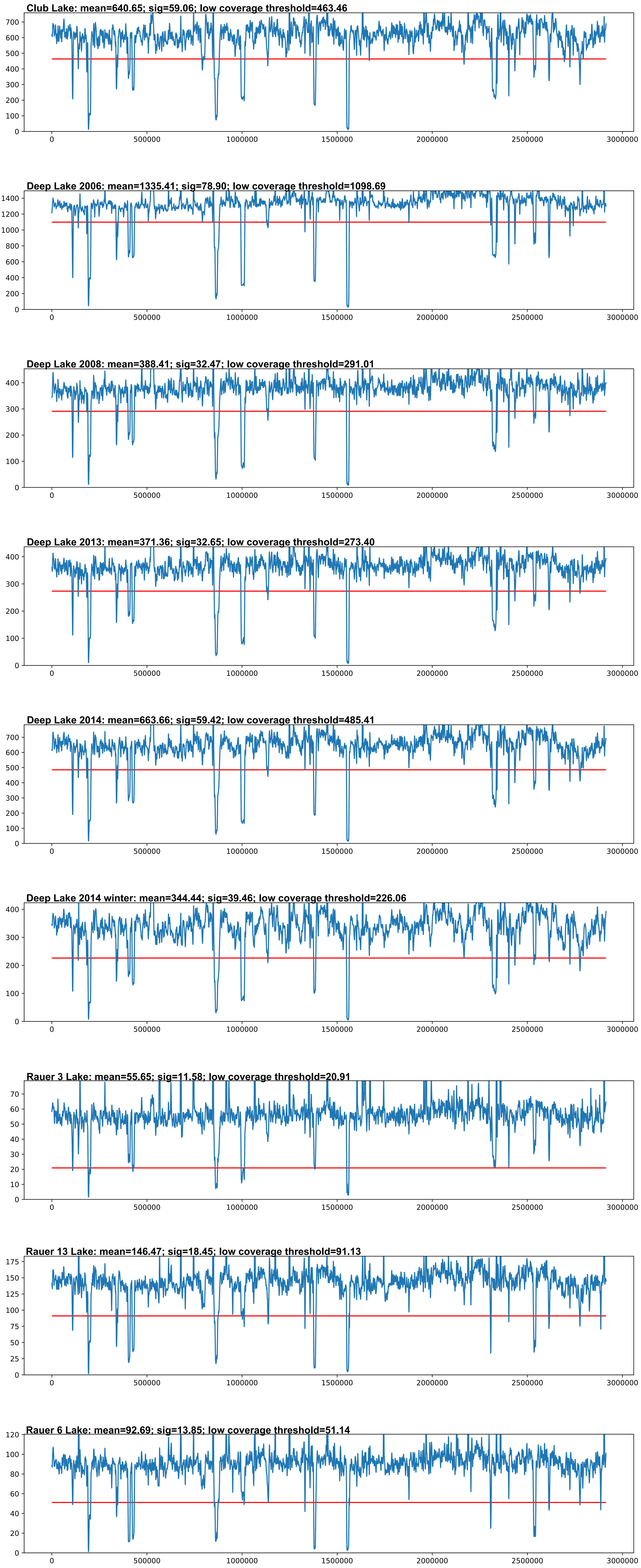

c)

Hht. litchfieldiae strain tADL, single replicon

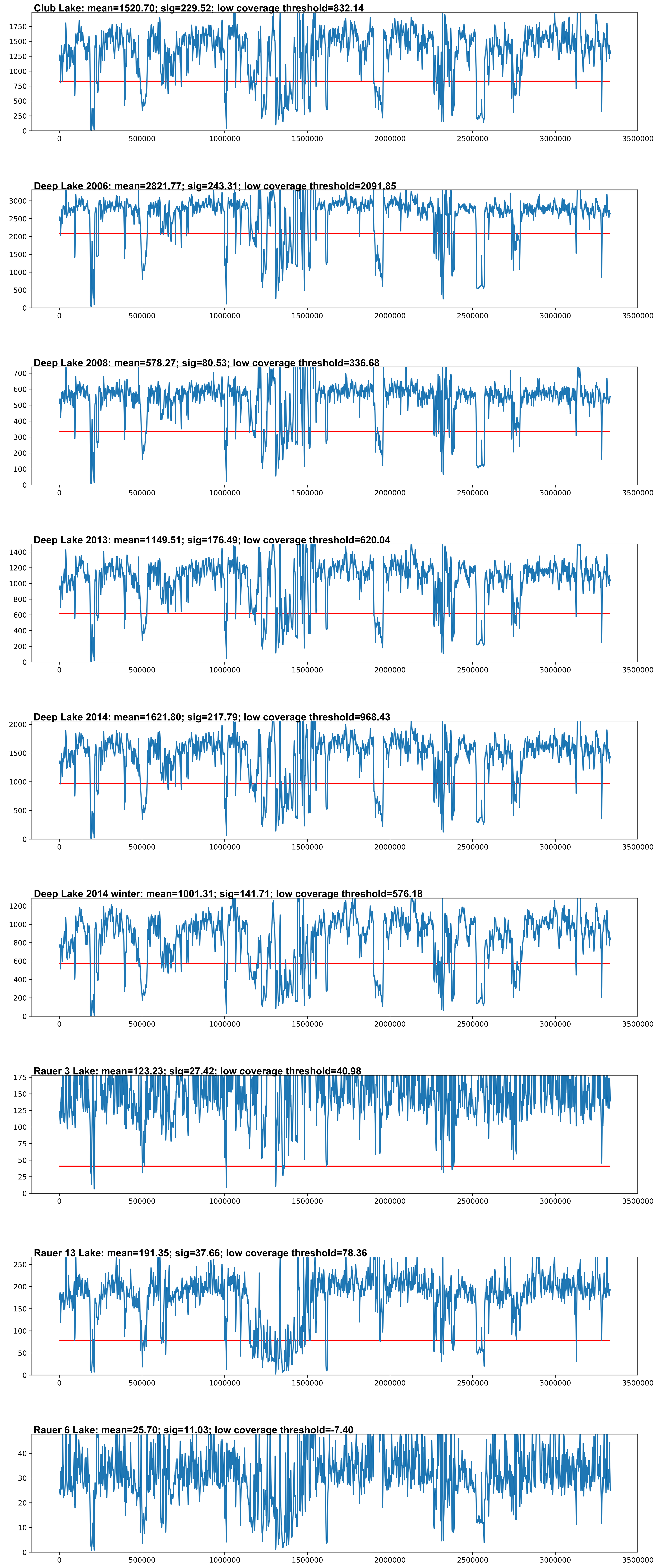

Figure S5 Genomic islands on primary replicons of Antarctic haloarchaea. Reads from nine pooled metagenomes (Additional file 2: Table S11) were mapped against the primary replicons of *Hrr. lacusprofundi* ACAM34 **(a)**, DL31 **(b)** and *Hht. litchfieldiae* tADL **(c)**. The plots show the coverage (y-axis) for each primary replicon and metagenome; the red line in each plot shows the coverage threshold (mean minus three standard deviations) that was used to define genomic islands. The primary replicon of *Hrr. lacusprofundi* ACAM34 had the least amount of genomic islands (average of ~31 kb), followed by DL31, and *Hht. litchfieldiae* tADL. Features on genomic islands of the ACAM34 primary replicon are highlighted: provirus Hlac-Pro1 (*) and S-layer gene (+). DL (Deep Lake).

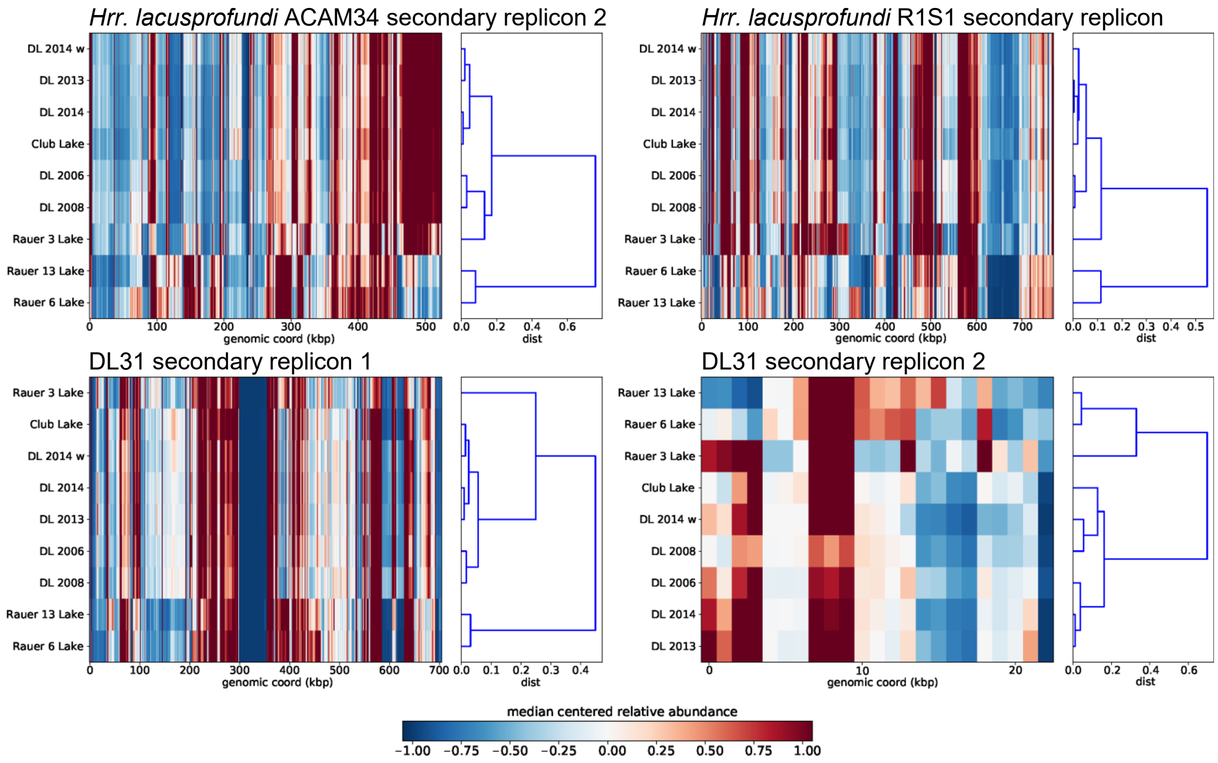

Figure S6 Metagenome coverage and HCA of selected secondary replicons. The figure shows the metagenome coverage and HCA of secondary replicons not included in Fig. 5. For R1S1, all secondary replicon contigs were concatenated into a single sequence. All metagenomes were from summer except for Deep Lake 2014 winter (w). DL (Deep Lake).

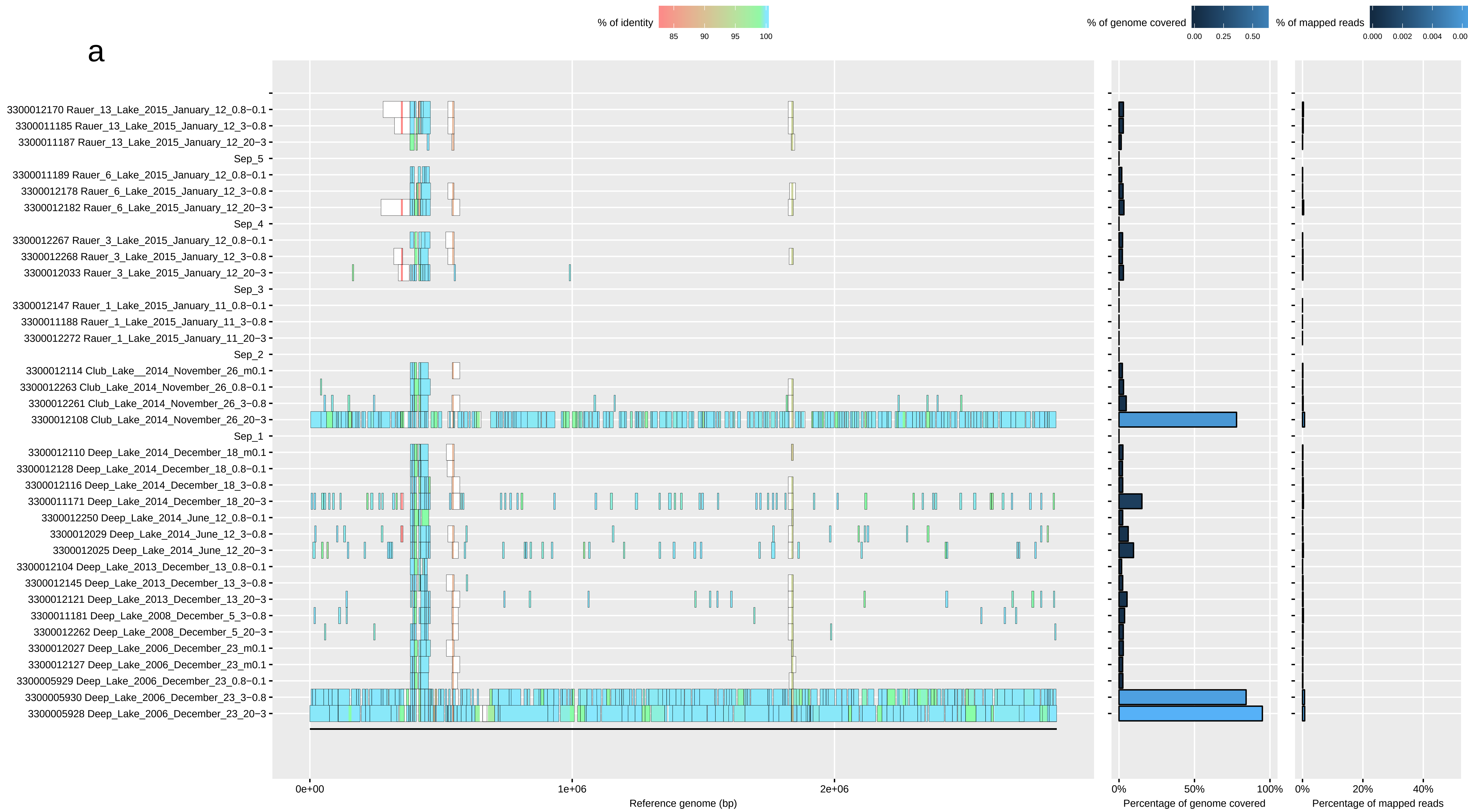

a
% of identity
85
90
95
100
% of genome covered
0.00
0.25
0.50
% of mapped reads
0.000
0.002
0.004
3300012170 Rauer_13_Lake_2015_January_12_0.8−0.1
3300011185 Rauer_13_Lake_2015_January_12_3−0.8
3300011187 Rauer_13_Lake_2015_January_12_20−3
Sep_5
3300011189 Rauer_6_Lake_2015_January_12_0.8−0.1
3300012178 Rauer_6_Lake_2015_January_12_3−0.8
3300012182 Rauer_6_Lake_2015_January_12_20−3
Sep_4
3300012267 Rauer_3_Lake_2015_January_12_0.8−0.1
3300012268 Rauer_3_Lake_2015_January_12_3−0.8
3300012033 Rauer_3_Lake_2015_January_12_20−3
Sep_3
3300012147 Rauer_1_Lake_2015_January_11_0.8−0.1
3300011188 Rauer_1_Lake_2015_January_11_3−0.8
3300012272 Rauer_1_Lake_2015_January_11_20−3
Sep_2
3300012114 Club_Lake__2014_November_26_m0.1
3300012263 Club_Lake_2014_November_26_0.8−0.1
3300012261 Club_Lake_2014_November_26_3−0.8
3300012108 Club_Lake_2014_November_26_20−3
Sep_1
3300012110 Deep_Lake_2014_December_18_m0.1
3300012128 Deep_Lake_2014_December_18_0.8−0.1
3300012116 Deep_Lake_2014_December_18_3−0.8
3300011171 Deep_Lake_2014_December_18_20−3
3300012250 Deep_Lake_2014_June_12_0.8−0.1
3300012029 Deep_Lake_2014_June_12_3−0.8
3300012025 Deep_Lake_2014_June_12_20−3
3300012104 Deep_Lake_2013_December_13_0.8−0.1
3300012145 Deep_Lake_2013_December_13_3−0.8
3300012121 Deep_Lake_2013_December_13_20−3
3300011181 Deep_Lake_2008_December_5_3−0.8
3300012262 Deep_Lake_2008_December_5_20−3
3300012027 Deep_Lake_2006_December_23_m0.1
3300012127 Deep_Lake_2006_December_23_m0.1
3300005929 Deep_Lake_2006_December_23_0.8−0.1
3300005930 Deep_Lake_2006_December_23_3−0.8
3300005928 Deep_Lake_2006_December_23_20−3
0e+00
1e+06
2e+06
Reference genome (bp)
0%
50%
100%
Percentage of genome covered
0%
20%
40%
Percentage of mapped reads

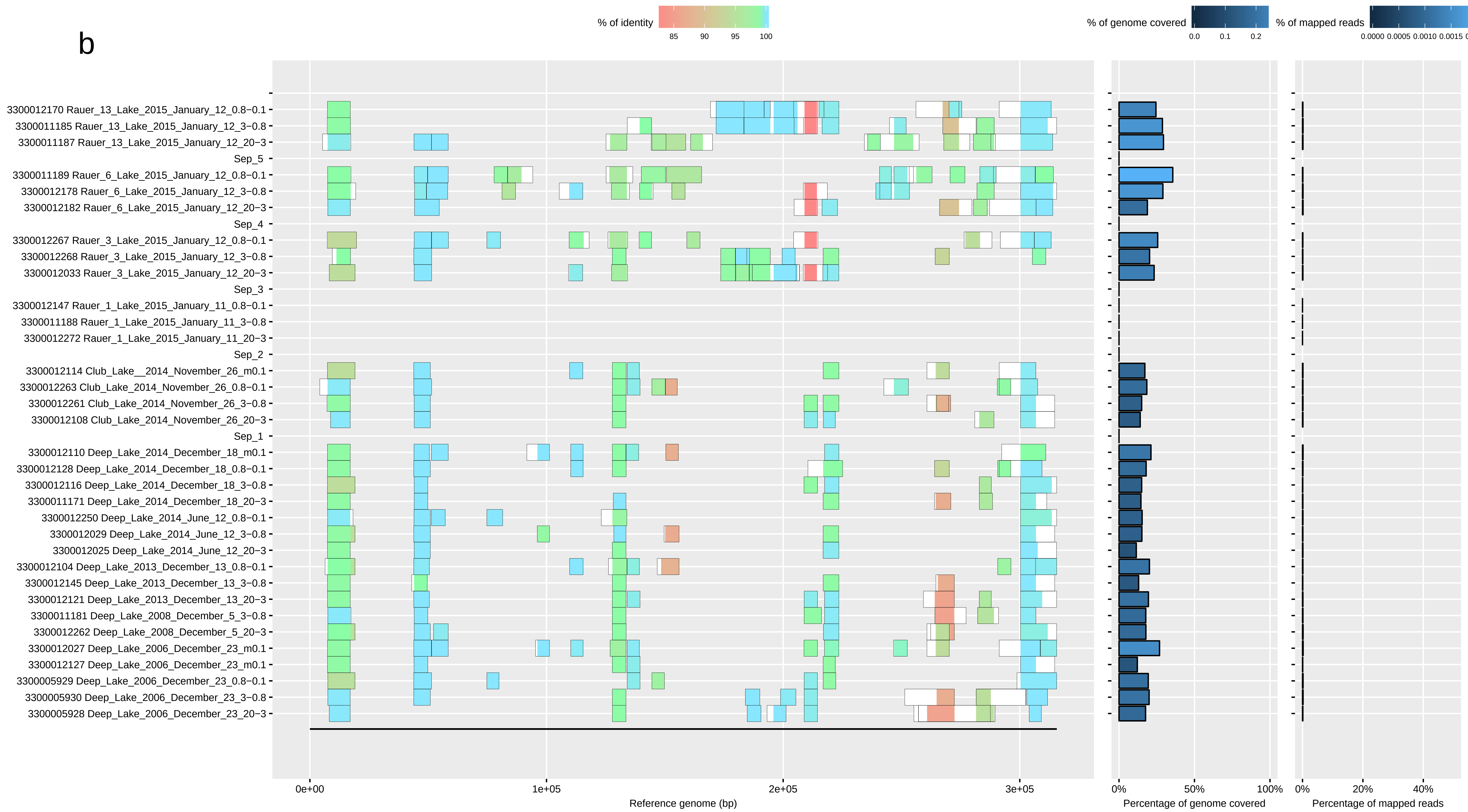

b
% of identity
85
90
95
100
% of genome covered
0.0
0.1
0.2
% of mapped reads
0.0000
0.0005
0.0010
0.0015
3300012170 Rauer_13_Lake_2015_January_12_0.8−0.1
3300011185 Rauer_13_Lake_2015_January_12_3−0.8
3300011187 Rauer_13_Lake_2015_January_12_20−3
Sep_5
3300011189 Rauer_6_Lake_2015_January_12_0.8−0.1
3300012178 Rauer_6_Lake_2015_January_12_3−0.8
3300012182 Rauer_6_Lake_2015_January_12_20−3
Sep_4
3300012267 Rauer_3_Lake_2015_January_12_0.8−0.1
3300012268 Rauer_3_Lake_2015_January_12_3−0.8
3300012033 Rauer_3_Lake_2015_January_12_20−3
Sep_3
3300012147 Rauer_1_Lake_2015_January_11_0.8−0.1
3300011188 Rauer_1_Lake_2015_January_11_3−0.8
3300012272 Rauer_1_Lake_2015_January_11_20−3
Sep_2
3300012114 Club_Lake__2014_November_26_m0.1
3300012263 Club_Lake_2014_November_26_0.8−0.1
3300012261 Club_Lake_2014_November_26_3−0.8
3300012108 Club_Lake_2014_November_26_20−3
Sep_1
3300012110 Deep_Lake_2014_December_18_m0.1
3300012128 Deep_Lake_2014_December_18_0.8−0.1
3300012116 Deep_Lake_2014_December_18_3−0.8
3300011171 Deep_Lake_2014_December_18_20−3
3300012250 Deep_Lake_2014_June_12_0.8−0.1
3300012029 Deep_Lake_2014_June_12_3−0.8
3300012025 Deep_Lake_2014_June_12_20−3
3300012104 Deep_Lake_2013_December_13_0.8−0.1
3300012145 Deep_Lake_2013_December_13_3−0.8
3300012121 Deep_Lake_2013_December_13_20−3
3300011181 Deep_Lake_2008_December_5_3−0.8
3300012262 Deep_Lake_2008_December_5_20−3
3300012027 Deep_Lake_2006_December_23_m0.1
3300012127 Deep_Lake_2006_December_23_m0.1
3300005929 Deep_Lake_2006_December_23_0.8−0.1
3300005930 Deep_Lake_2006_December_23_3−0.8
3300005928 Deep_Lake_2006_December_23_20−3
0e+00
1e+05
2e+05
3e+05
Reference genome (bp)
0%
50%
100%
Percentage of genome covered
0%
20%
40%
Percentage of mapped reads

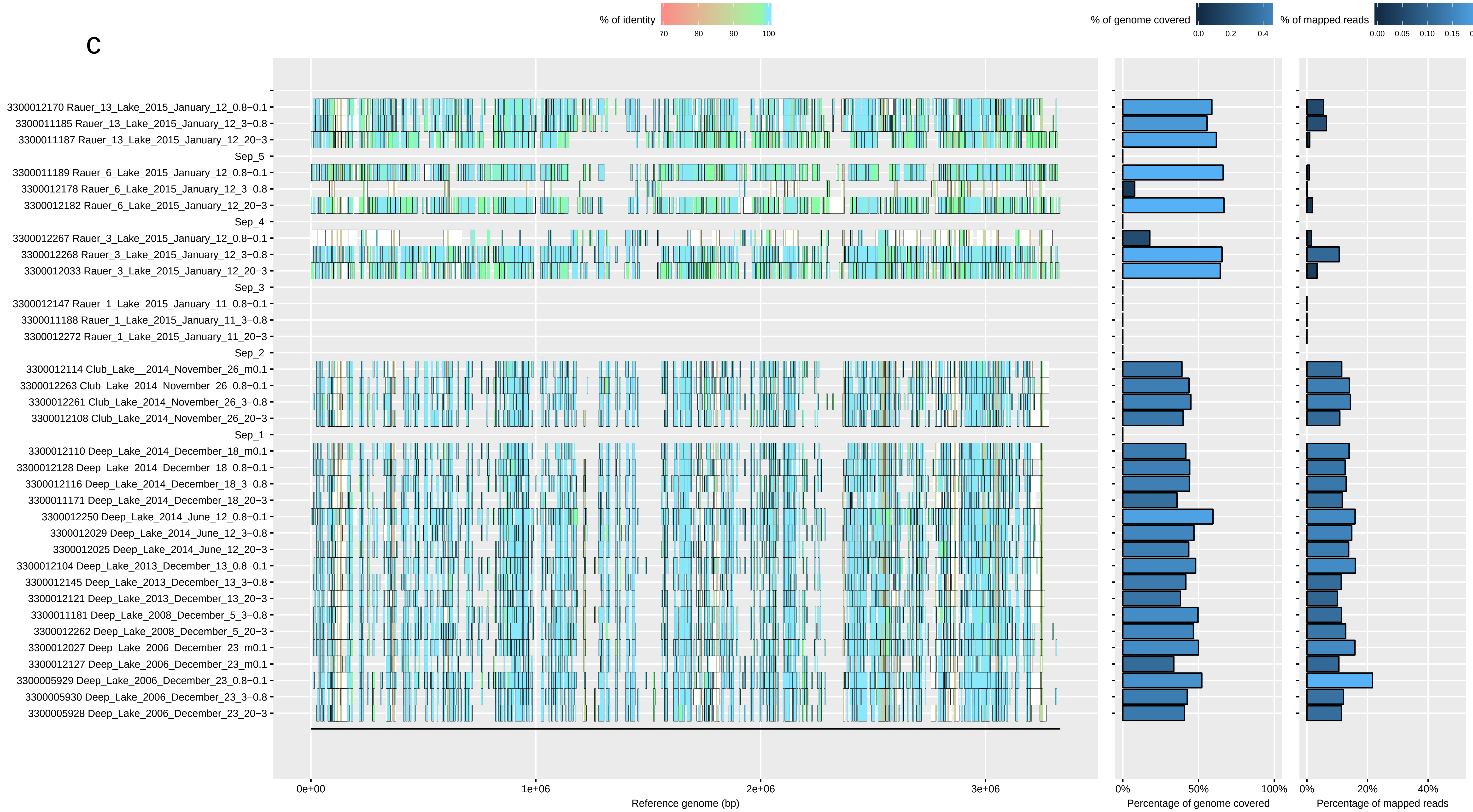

c
% of identity
70
80
90
100
% of genome covered
0.0
0.2
0.4
% of mapped reads
0.00
0.05
0.10
0.15
3300012170 Rauer_13_Lake_2015_January_12_0.8−0.1
3300011185 Rauer_13_Lake_2015_January_12_3−0.8
3300011187 Rauer_13_Lake_2015_January_12_20−3
Sep_5
3300011189 Rauer_6_Lake_2015_January_12_0.8−0.1
3300012178 Rauer_6_Lake_2015_January_12_3−0.8
3300012182 Rauer_6_Lake_2015_January_12_20−3
Sep_4
3300012267 Rauer_3_Lake_2015_January_12_0.8−0.1
3300012268 Rauer_3_Lake_2015_January_12_3−0.8
3300012033 Rauer_3_Lake_2015_January_12_20−3
Sep_3
3300012147 Rauer_1_Lake_2015_January_11_0.8−0.1
3300011188 Rauer_1_Lake_2015_January_11_3−0.8
3300012272 Rauer_1_Lake_2015_January_11_20−3
Sep_2
3300012114 Club_Lake__2014_November_26_m0.1
3300012263 Club_Lake_2014_November_26_0.8−0.1
3300012261 Club_Lake_2014_November_26_3−0.8
3300012108 Club_Lake_2014_November_26_20−3
Sep_1
3300012110 Deep_Lake_2014_December_18_m0.1
3300012128 Deep_Lake_2014_December_18_0.8−0.1
3300012116 Deep_Lake_2014_December_18_3−0.8
3300011171 Deep_Lake_2014_December_18_20−3
3300012250 Deep_Lake_2014_June_12_0.8−0.1
3300012029 Deep_Lake_2014_June_12_3−0.8
3300012025 Deep_Lake_2014_June_12_20−3
3300012104 Deep_Lake_2013_December_13_0.8−0.1
3300012145 Deep_Lake_2013_December_13_3−0.8
3300012121 Deep_Lake_2013_December_13_20−3
3300011181 Deep_Lake_2008_December_5_3−0.8
3300012262 Deep_Lake_2008_December_5_20−3
3300012027 Deep_Lake_2006_December_23_m0.1
3300012127 Deep_Lake_2006_December_23_m0.1
3300005929 Deep_Lake_2006_December_23_0.8−0.1
3300005930 Deep_Lake_2006_December_23_3−0.8
3300005928 Deep_Lake_2006_December_23_20−3
0e+00
1e+06
2e+06
3e+06
Reference genome (bp)
0%
50%
100%
Percentage of genome covered
0%
20%
40%
Percentage of mapped reads

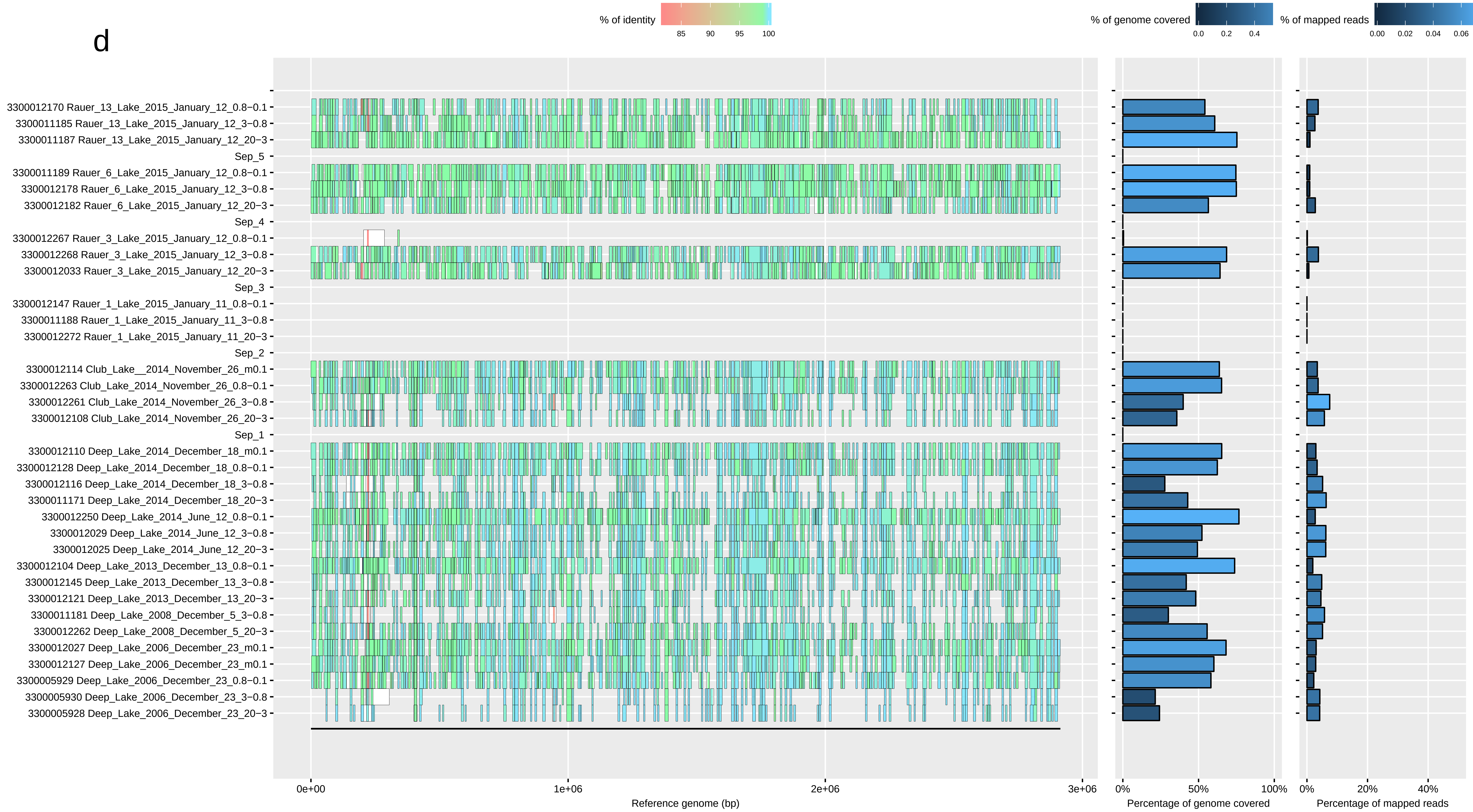
d
% of identity
85
90
95
100
% of genome covered
0.0
0.2
0.4
% of mapped reads
0.00
0.02
0.04
0.06
3300012170 Rauer_13_Lake_2015_January_12_0.8−0.1
3300011185 Rauer_13_Lake_2015_January_12_3−0.8
3300011187 Rauer_13_Lake_2015_January_12_20−3
Sep_5
3300011189 Rauer_6_Lake_2015_January_12_0.8−0.1
3300012178 Rauer_6_Lake_2015_January_12_3−0.8
3300012182 Rauer_6_Lake_2015_January_12_20−3
Sep_4
3300012267 Rauer_3_Lake_2015_January_12_0.8−0.1
3300012268 Rauer_3_Lake_2015_January_12_3−0.8
3300012033 Rauer_3_Lake_2015_January_12_20−3
Sep_3
3300012147 Rauer_1_Lake_2015_January_11_0.8−0.1
3300011188 Rauer_1_Lake_2015_January_11_3−0.8
3300012272 Rauer_1_Lake_2015_January_11_20−3
Sep_2
3300012114 Club_Lake__2014_November_26_m0.1
3300012263 Club_Lake_2014_November_26_0.8−0.1
3300012261 Club_Lake_2014_November_26_3−0.8
3300012108 Club_Lake_2014_November_26_20−3
Sep_1
3300012110 Deep_Lake_2014_December_18_m0.1
3300012128 Deep_Lake_2014_December_18_0.8−0.1
3300012116 Deep_Lake_2014_December_18_3−0.8
3300011171 Deep_Lake_2014_December_18_20−3
3300012250 Deep_Lake_2014_June_12_0.8−0.1
3300012029 Deep_Lake_2014_June_12_3−0.8
3300012025 Deep_Lake_2014_June_12_20−3
3300012104 Deep_Lake_2013_December_13_0.8−0.1
3300012145 Deep_Lake_2013_December_13_3−0.8
3300012121 Deep_Lake_2013_December_13_20−3
3300011181 Deep_Lake_2008_December_5_3−0.8
3300012262 Deep_Lake_2008_December_5_20−3
3300012027 Deep_Lake_2006_December_23_m0.1
3300012127 Deep_Lake_2006_December_23_m0.1
3300005929 Deep_Lake_2006_December_23_0.8−0.1
3300005930 Deep_Lake_2006_December_23_3−0.8
3300005928 Deep_Lake_2006_December_23_20−3
0e+00
1e+06
2e+06
3e+06
Reference genome (bp)
0%
50%
100%
Percentage of genome covered
0%
20%
40%
Percentage of mapped reads

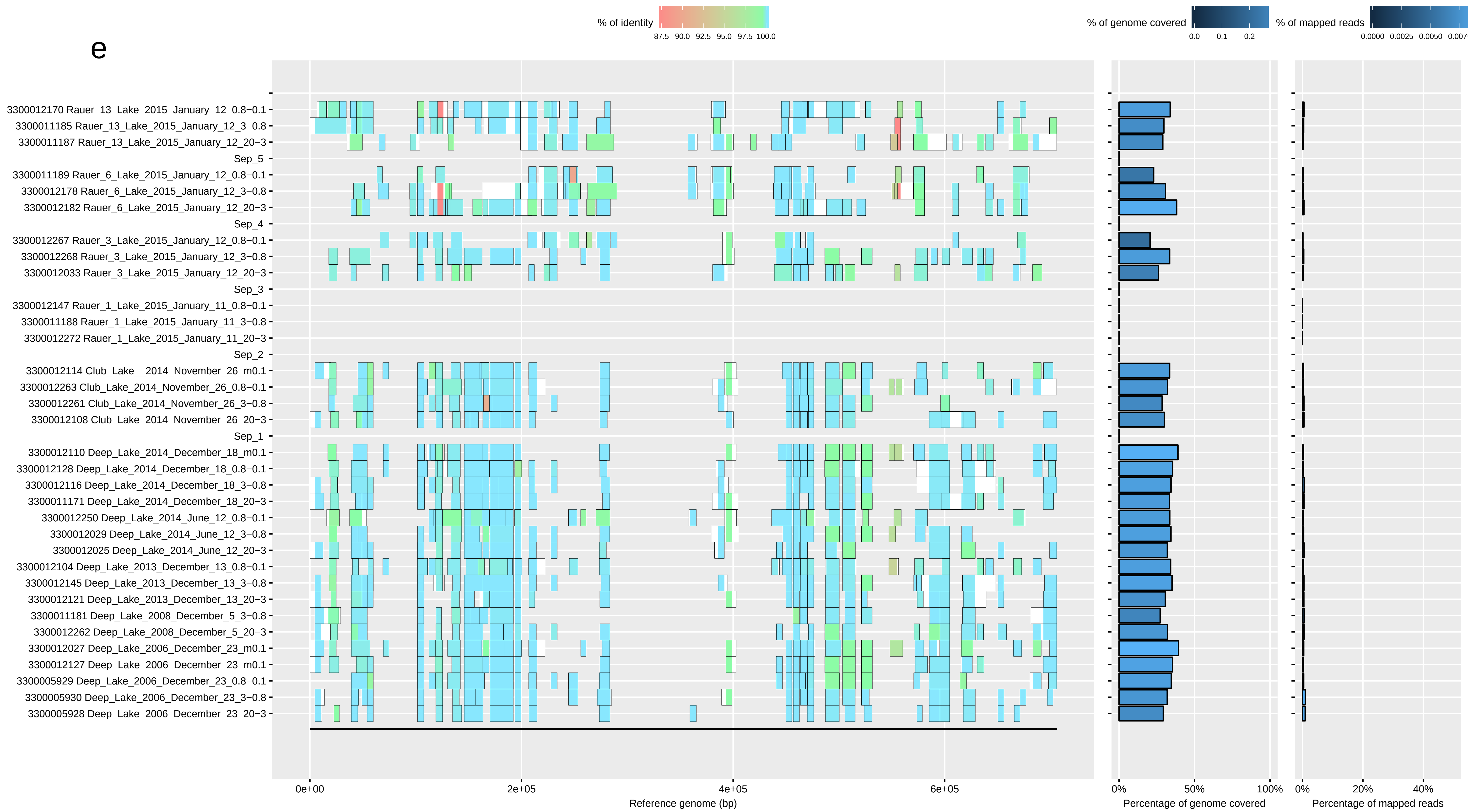
e
% of identity
87.5 90.0 92.5 95.0 97.5 100.0
% of genome covered
0.0 0.1 0.2
% of mapped reads
0.0000 0.0025 0.0050
3300012170 Rauer_13_Lake_2015_January_12_0.8−0.1
3300011185 Rauer_13_Lake_2015_January_12_3−0.8
3300011187 Rauer_13_Lake_2015_January_12_20−3
Sep_5
3300011189 Rauer_6_Lake_2015_January_12_0.8−0.1
3300012178 Rauer_6_Lake_2015_January_12_3−0.8
3300012182 Rauer_6_Lake_2015_January_12_20−3
Sep_4
3300012267 Rauer_3_Lake_2015_January_12_0.8−0.1
3300012268 Rauer_3_Lake_2015_January_12_3−0.8
3300012033 Rauer_3_Lake_2015_January_12_20−3
Sep_3
3300012147 Rauer_1_Lake_2015_January_11_0.8−0.1
3300011188 Rauer_1_Lake_2015_January_11_3−0.8
3300012272 Rauer_1_Lake_2015_January_11_20−3
Sep_2
3300012114 Club_Lake__2014_November_26_m0.1
3300012263 Club_Lake_2014_November_26_0.8−0.1
3300012261 Club_Lake_2014_November_26_3−0.8
3300012108 Club_Lake_2014_November_26_20−3
Sep_1
3300012110 Deep_Lake_2014_December_18_m0.1
3300012128 Deep_Lake_2014_December_18_0.8−0.1
3300012116 Deep_Lake_2014_December_18_3−0.8
3300011171 Deep_Lake_2014_December_18_20−3
3300012250 Deep_Lake_2014_June_12_0.8−0.1
3300012029 Deep_Lake_2014_June_12_3−0.8
3300012025 Deep_Lake_2014_June_12_20−3
3300012104 Deep_Lake_2013_December_13_0.8−0.1
3300012145 Deep_Lake_2013_December_13_3−0.8
3300012121 Deep_Lake_2013_December_13_20−3
3300011181 Deep_Lake_2008_December_5_3−0.8
3300012262 Deep_Lake_2008_December_5_20−3
3300012027 Deep_Lake_2006_December_23_m0.1
3300012127 Deep_Lake_2006_December_23_m0.1
3300005929 Deep_Lake_2006_December_23_0.8−0.1
3300005930 Deep_Lake_2006_December_23_3−0.8
3300005928 Deep_Lake_2006_December_23_20−3
0e+00
2e+05
4e+05
6e+05
Reference genome (bp)
0%
50%
100%
Percentage of genome covered
0%
20%
40%
Percentage of mapped reads

f

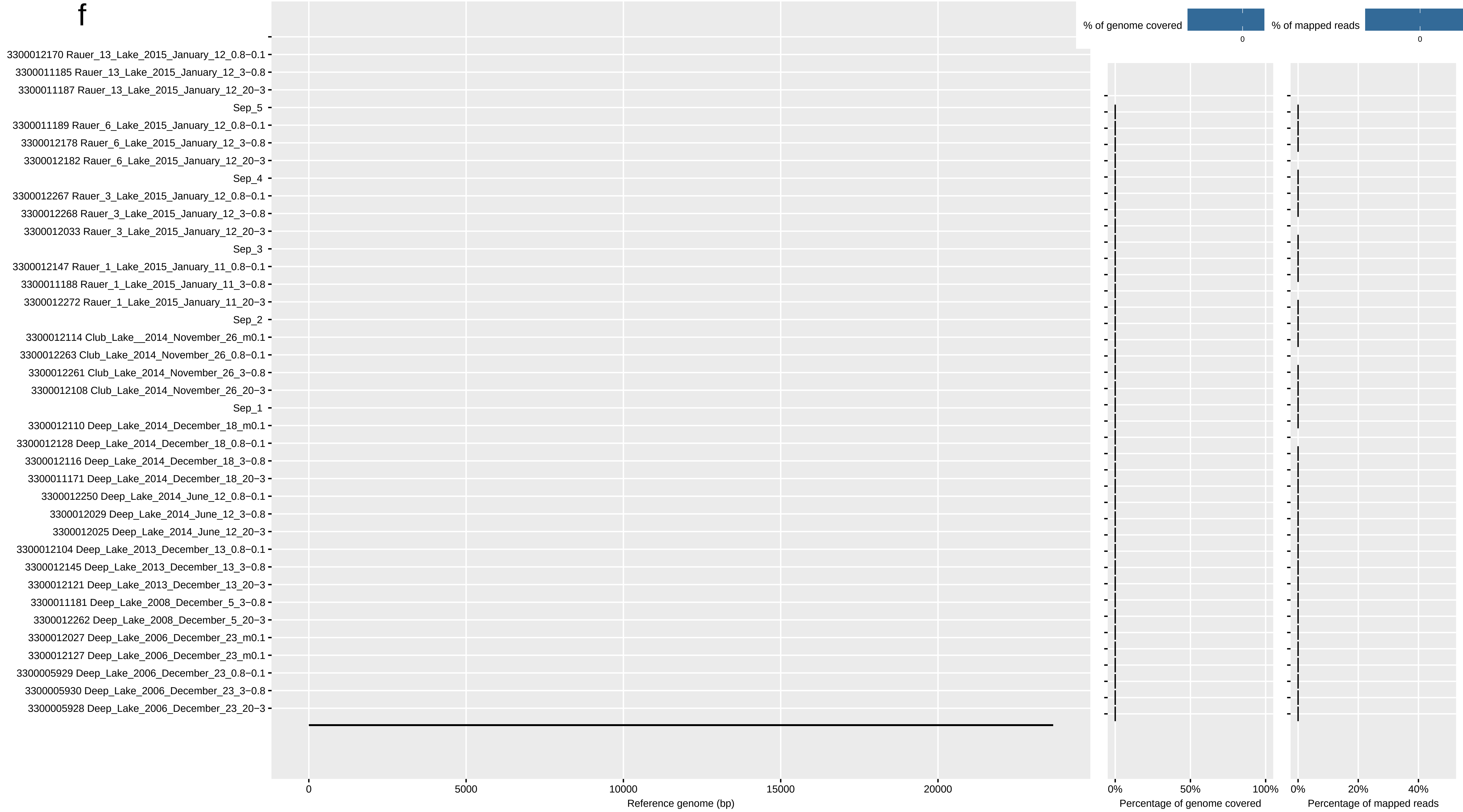

% of genome covered
0
% of mapped reads
0
3300012170 Rauer_13_Lake_2015_January_12_0.8−0.1
3300011185 Rauer_13_Lake_2015_January_12_3−0.8
3300011187 Rauer_13_Lake_2015_January_12_20−3
Sep_5
3300011189 Rauer_6_Lake_2015_January_12_0.8−0.1
3300012178 Rauer_6_Lake_2015_January_12_3−0.8
3300012182 Rauer_6_Lake_2015_January_12_20−3
Sep_4
3300012267 Rauer_3_Lake_2015_January_12_0.8−0.1
3300012268 Rauer_3_Lake_2015_January_12_3−0.8
3300012033 Rauer_3_Lake_2015_January_12_20−3
Sep_3
3300012147 Rauer_1_Lake_2015_January_11_0.8−0.1
3300011188 Rauer_1_Lake_2015_January_11_3−0.8
3300012272 Rauer_1_Lake_2015_January_11_20−3
Sep_2
3300012114 Club_Lake__2014_November_26_m0.1
3300012263 Club_Lake_2014_November_26_0.8−0.1
3300012261 Club_Lake_2014_November_26_3−0.8
3300012108 Club_Lake_2014_November_26_20−3
Sep_1
3300012110 Deep_Lake_2014_December_18_m0.1
3300012128 Deep_Lake_2014_December_18_0.8−0.1
3300012116 Deep_Lake_2014_December_18_3−0.8
3300011171 Deep_Lake_2014_December_18_20−3
3300012250 Deep_Lake_2014_June_12_0.8−0.1
3300012029 Deep_Lake_2014_June_12_3−0.8
3300012025 Deep_Lake_2014_June_12_20−3
3300012104 Deep_Lake_2013_December_13_0.8−0.1
3300012145 Deep_Lake_2013_December_13_3−0.8
3300012121 Deep_Lake_2013_December_13_20−3
3300011181 Deep_Lake_2008_December_5_3−0.8
3300012262 Deep_Lake_2008_December_5_20−3
3300012027 Deep_Lake_2006_December_23_m0.1
3300012127 Deep_Lake_2006_December_23_m0.1
3300005929 Deep_Lake_2006_December_23_0.8−0.1
3300005930 Deep_Lake_2006_December_23_3−0.8
3300005928 Deep_Lake_2006_December_23_20−3
0
5000
10000
15000
20000
Reference genome (bp)
0%
50%
100%
Percentage of genome covered
0%
20%
40%
Percentage of mapped reads

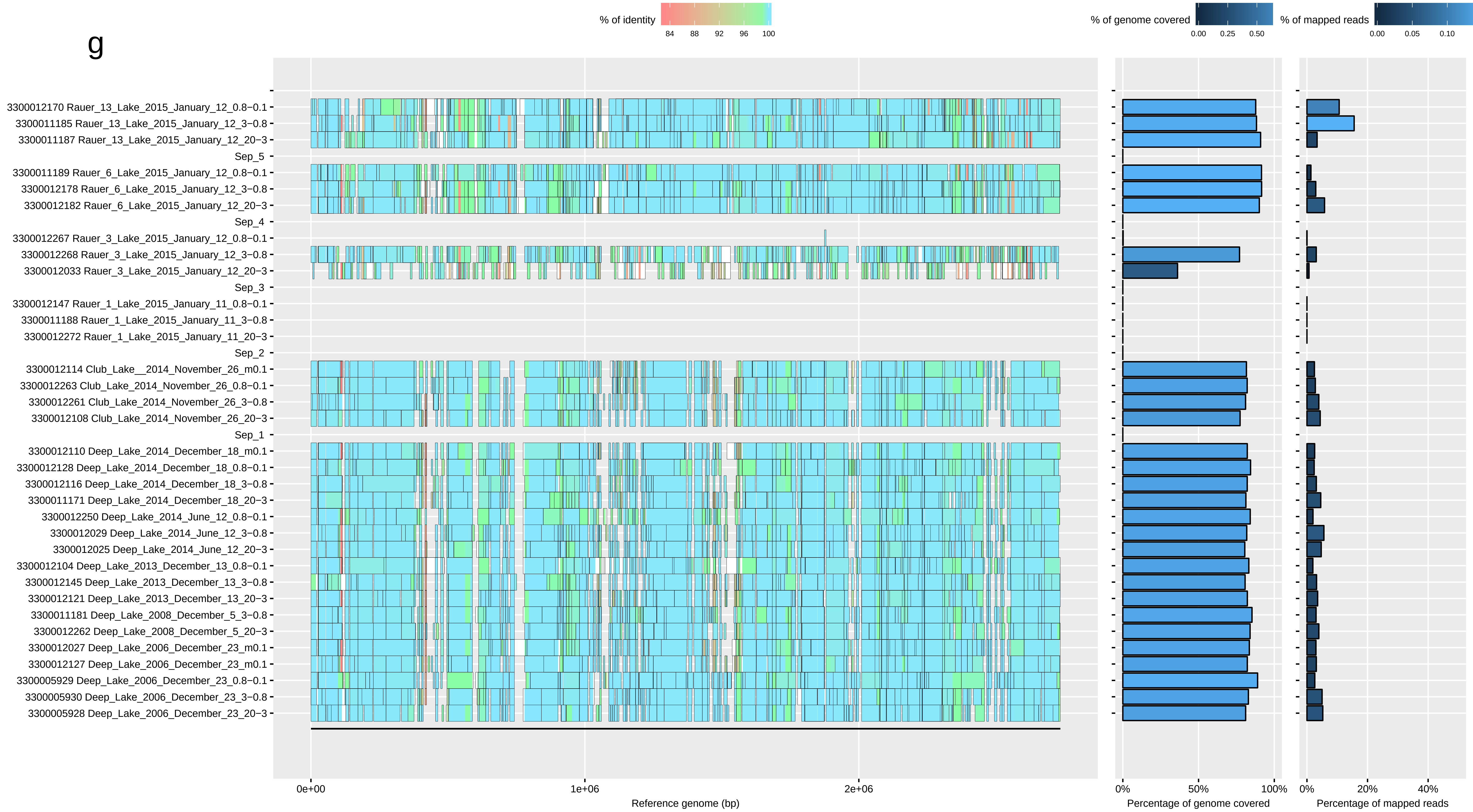
g
% of identity
84
88
92
96
100
% of genome covered
0.00
0.25
0.50
% of mapped reads
0.00
0.05
0.10
3300012170 Rauer_13_Lake_2015_January_12_0.8−0.1
3300011185 Rauer_13_Lake_2015_January_12_3−0.8
3300011187 Rauer_13_Lake_2015_January_12_20−3
Sep_5
3300011189 Rauer_6_Lake_2015_January_12_0.8−0.1
3300012178 Rauer_6_Lake_2015_January_12_3−0.8
3300012182 Rauer_6_Lake_2015_January_12_20−3
Sep_4
3300012267 Rauer_3_Lake_2015_January_12_0.8−0.1
3300012268 Rauer_3_Lake_2015_January_12_3−0.8
3300012033 Rauer_3_Lake_2015_January_12_20−3
Sep_3
3300012147 Rauer_1_Lake_2015_January_11_0.8−0.1
3300011188 Rauer_1_Lake_2015_January_11_3−0.8
3300012272 Rauer_1_Lake_2015_January_11_20−3
Sep_2
3300012114 Club_Lake__2014_November_26_m0.1
3300012263 Club_Lake_2014_November_26_0.8−0.1
3300012261 Club_Lake_2014_November_26_3−0.8
3300012108 Club_Lake_2014_November_26_20−3
Sep_1
3300012110 Deep_Lake_2014_December_18_m0.1
3300012128 Deep_Lake_2014_December_18_0.8−0.1
3300012116 Deep_Lake_2014_December_18_3−0.8
3300011171 Deep_Lake_2014_December_18_20−3
3300012250 Deep_Lake_2014_June_12_0.8−0.1
3300012029 Deep_Lake_2014_June_12_3−0.8
3300012025 Deep_Lake_2014_June_12_20−3
3300012104 Deep_Lake_2013_December_13_0.8−0.1
3300012145 Deep_Lake_2013_December_13_3−0.8
3300012121 Deep_Lake_2013_December_13_20−3
3300011181 Deep_Lake_2008_December_5_3−0.8
3300012262 Deep_Lake_2008_December_5_20−3
3300012027 Deep_Lake_2006_December_23_m0.1
3300012127 Deep_Lake_2006_December_23_m0.1
3300005929 Deep_Lake_2006_December_23_0.8−0.1
3300005930 Deep_Lake_2006_December_23_3−0.8
3300005928 Deep_Lake_2006_December_23_20−3
0e+00
1e+06
2e+06
Reference genome (bp)
0%
50%
100%
Percentage of genome covered
0%
20%
40%
Percentage of mapped reads

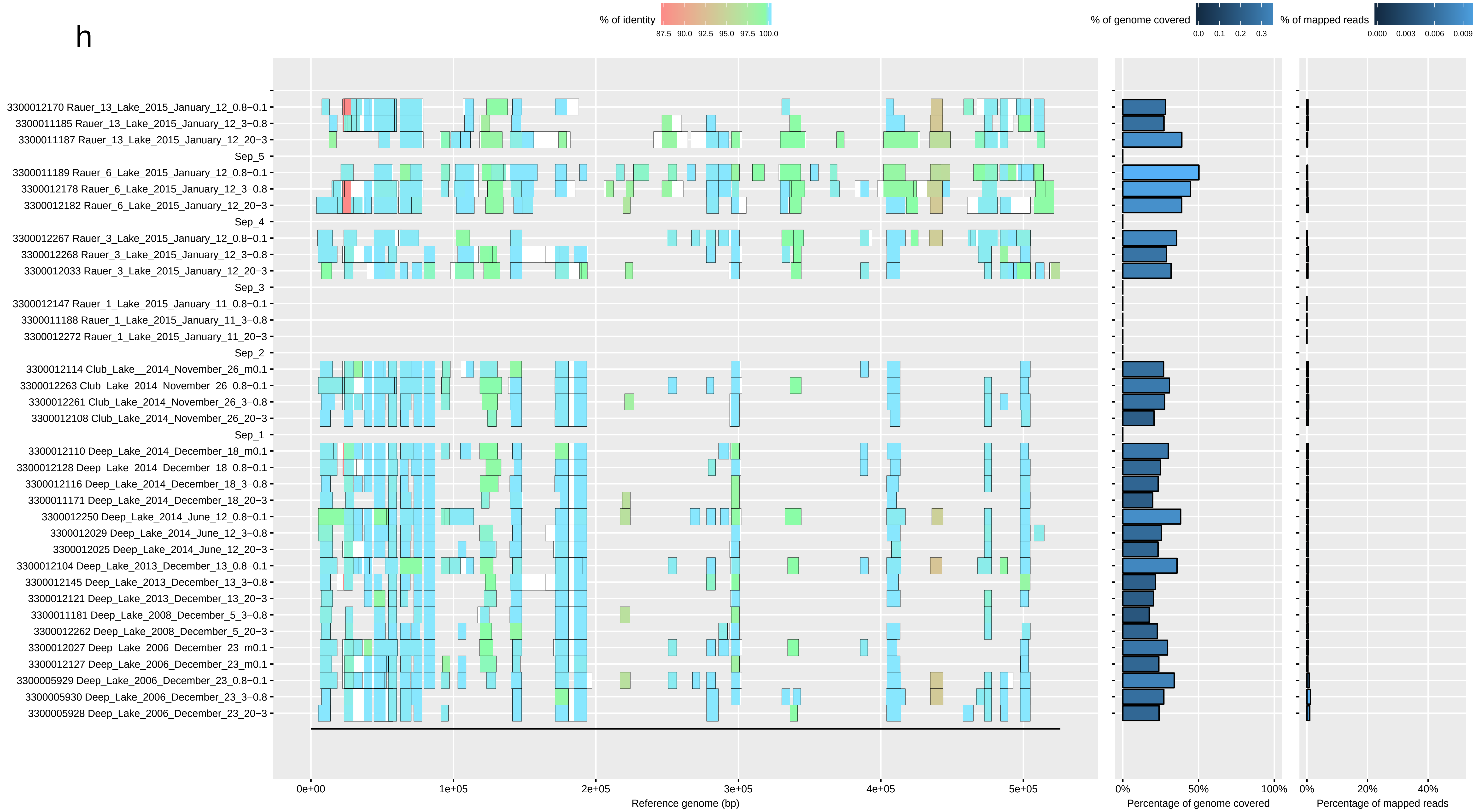
h
% of identity
87.5 90.0 92.5 95.0 97.5 100.0
% of genome covered
0.0 0.1 0.2 0.3
% of mapped reads
0.000 0.003 0.006 0.009
3300012170 Rauer_13_Lake_2015_January_12_0.8−0.1
3300011185 Rauer_13_Lake_2015_January_12_3−0.8
3300011187 Rauer_13_Lake_2015_January_12_20−3
Sep_5
3300011189 Rauer_6_Lake_2015_January_12_0.8−0.1
3300012178 Rauer_6_Lake_2015_January_12_3−0.8
3300012182 Rauer_6_Lake_2015_January_12_20−3
Sep_4
3300012267 Rauer_3_Lake_2015_January_12_0.8−0.1
3300012268 Rauer_3_Lake_2015_January_12_3−0.8
3300012033 Rauer_3_Lake_2015_January_12_20−3
Sep_3
3300012147 Rauer_1_Lake_2015_January_11_0.8−0.1
3300011188 Rauer_1_Lake_2015_January_11_3−0.8
3300012272 Rauer_1_Lake_2015_January_11_20−3
Sep_2
3300012114 Club_Lake__2014_November_26_m0.1
3300012263 Club_Lake_2014_November_26_0.8−0.1
3300012261 Club_Lake_2014_November_26_3−0.8
3300012108 Club_Lake_2014_November_26_20−3
Sep_1
3300012110 Deep_Lake_2014_December_18_m0.1
3300012128 Deep_Lake_2014_December_18_0.8−0.1
3300012116 Deep_Lake_2014_December_18_3−0.8
3300011171 Deep_Lake_2014_December_18_20−3
3300012250 Deep_Lake_2014_June_12_0.8−0.1
3300012029 Deep_Lake_2014_June_12_3−0.8
3300012025 Deep_Lake_2014_June_12_20−3
3300012104 Deep_Lake_2013_December_13_0.8−0.1
3300012145 Deep_Lake_2013_December_13_3−0.8
3300012121 Deep_Lake_2013_December_13_20−3
3300011181 Deep_Lake_2008_December_5_3−0.8
3300012262 Deep_Lake_2008_December_5_20−3
3300012027 Deep_Lake_2006_December_23_m0.1
3300012127 Deep_Lake_2006_December_23_m0.1
3300005929 Deep_Lake_2006_December_23_0.8−0.1
3300005930 Deep_Lake_2006_December_23_3−0.8
3300005928 Deep_Lake_2006_December_23_20−3
0e+00
1e+05
2e+05
3e+05
4e+05
5e+05
Reference genome (bp)
0%
50%
100%
Percentage of genome covered
0%
20%
40%
Percentage of mapped reads

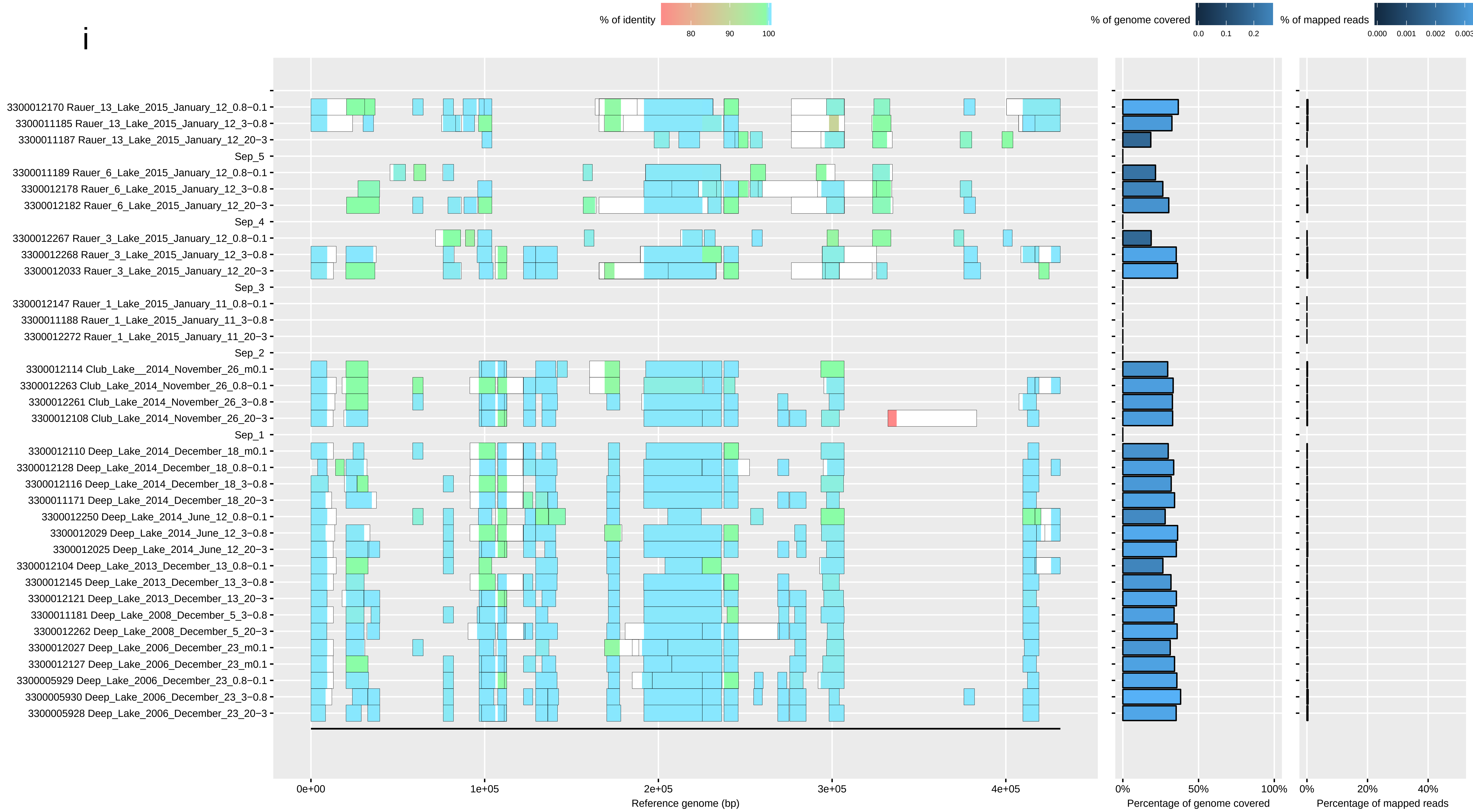

i
% of identity
80
90
100
% of genome covered
0.0
0.1
0.2
% of mapped reads
0.000
0.001
0.002
0.003
3300012170 Rauer_13_Lake_2015_January_12_0.8−0.1
3300011185 Rauer_13_Lake_2015_January_12_3−0.8
3300011187 Rauer_13_Lake_2015_January_12_20−3
Sep_5
3300011189 Rauer_6_Lake_2015_January_12_0.8−0.1
3300012178 Rauer_6_Lake_2015_January_12_3−0.8
3300012182 Rauer_6_Lake_2015_January_12_20−3
Sep_4
3300012267 Rauer_3_Lake_2015_January_12_0.8−0.1
3300012268 Rauer_3_Lake_2015_January_12_3−0.8
3300012033 Rauer_3_Lake_2015_January_12_20−3
Sep_3
3300012147 Rauer_1_Lake_2015_January_11_0.8−0.1
3300011188 Rauer_1_Lake_2015_January_11_3−0.8
3300012272 Rauer_1_Lake_2015_January_11_20−3
Sep_2
3300012114 Club_Lake__2014_November_26_m0.1
3300012263 Club_Lake_2014_November_26_0.8−0.1
3300012261 Club_Lake_2014_November_26_3−0.8
3300012108 Club_Lake_2014_November_26_20−3
Sep_1
3300012110 Deep_Lake_2014_December_18_m0.1
3300012128 Deep_Lake_2014_December_18_0.8−0.1
3300012116 Deep_Lake_2014_December_18_3−0.8
3300011171 Deep_Lake_2014_December_18_20−3
3300012250 Deep_Lake_2014_June_12_0.8−0.1
3300012029 Deep_Lake_2014_June_12_3−0.8
3300012025 Deep_Lake_2014_June_12_20−3
3300012104 Deep_Lake_2013_December_13_0.8−0.1
3300012145 Deep_Lake_2013_December_13_3−0.8
3300012121 Deep_Lake_2013_December_13_20−3
3300011181 Deep_Lake_2008_December_5_3−0.8
3300012262 Deep_Lake_2008_December_5_20−3
3300012027 Deep_Lake_2006_December_23_m0.1
3300012127 Deep_Lake_2006_December_23_m0.1
3300005929 Deep_Lake_2006_December_23_0.8−0.1
3300005930 Deep_Lake_2006_December_23_3−0.8
3300005928 Deep_Lake_2006_December_23_20−3
0e+00
1e+05
2e+05
3e+05
4e+05
Reference genome (bp)
0%
50%
100%
Percentage of genome covered
0%
20%
40%
Percentage of mapped reads

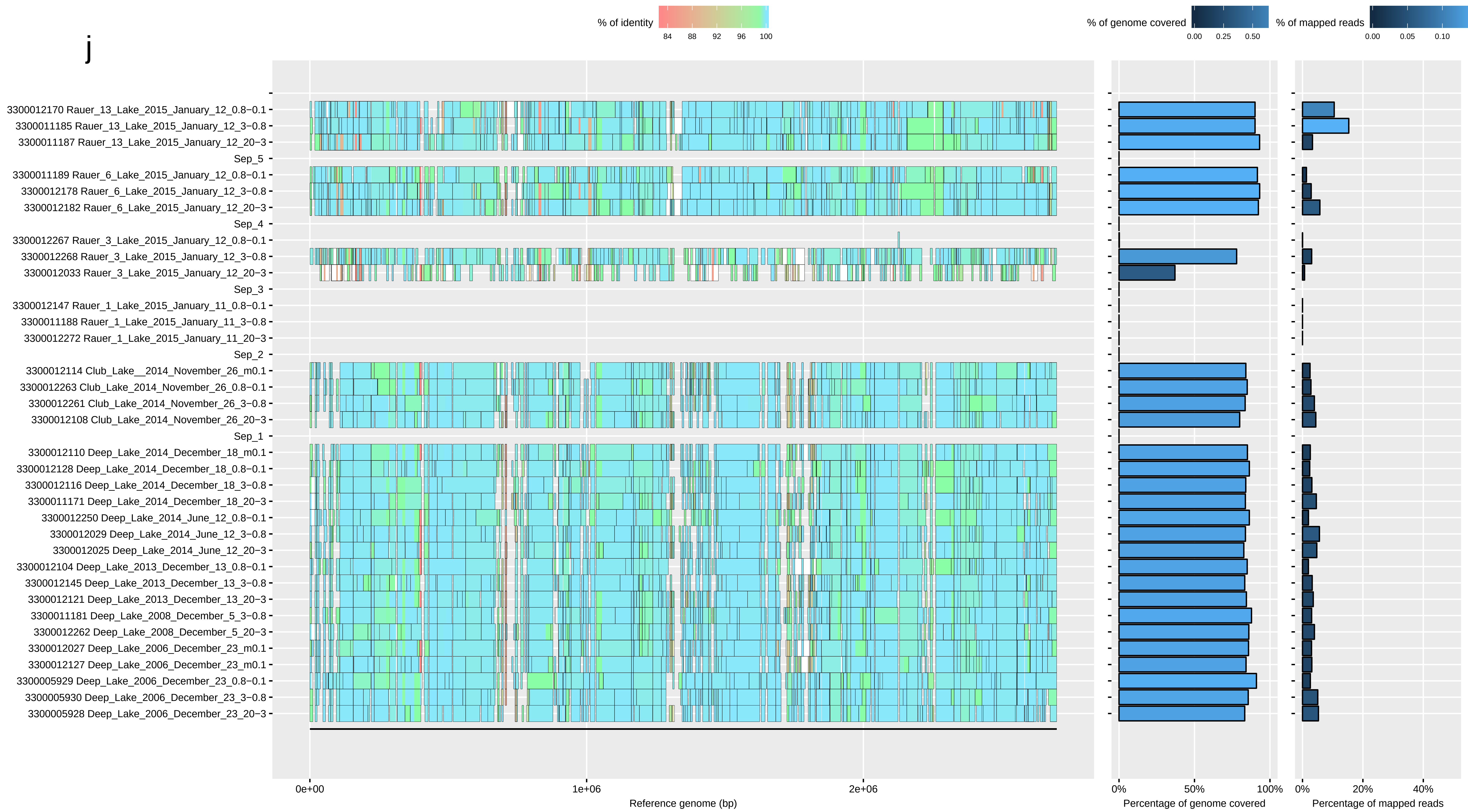

j
% of identity
84
88
92
96
100
% of genome covered
0.00
0.25
0.50
% of mapped reads
0.00
0.05
0.10
3300012170 Rauer_13_Lake_2015_January_12_0.8−0.1
3300011185 Rauer_13_Lake_2015_January_12_3−0.8
3300011187 Rauer_13_Lake_2015_January_12_20−3
Sep_5
3300011189 Rauer_6_Lake_2015_January_12_0.8−0.1
3300012178 Rauer_6_Lake_2015_January_12_3−0.8
3300012182 Rauer_6_Lake_2015_January_12_20−3
Sep_4
3300012267 Rauer_3_Lake_2015_January_12_0.8−0.1
3300012268 Rauer_3_Lake_2015_January_12_3−0.8
3300012033 Rauer_3_Lake_2015_January_12_20−3
Sep_3
3300012147 Rauer_1_Lake_2015_January_11_0.8−0.1
3300011188 Rauer_1_Lake_2015_January_11_3−0.8
3300012272 Rauer_1_Lake_2015_January_11_20−3
Sep_2
3300012114 Club_Lake__2014_November_26_m0.1
3300012263 Club_Lake_2014_November_26_0.8−0.1
3300012261 Club_Lake_2014_November_26_3−0.8
3300012108 Club_Lake_2014_November_26_20−3
Sep_1
3300012110 Deep_Lake_2014_December_18_m0.1
3300012128 Deep_Lake_2014_December_18_0.8−0.1
3300012116 Deep_Lake_2014_December_18_3−0.8
3300011171 Deep_Lake_2014_December_18_20−3
3300012250 Deep_Lake_2014_June_12_0.8−0.1
3300012029 Deep_Lake_2014_June_12_3−0.8
3300012025 Deep_Lake_2014_June_12_20−3
3300012104 Deep_Lake_2013_December_13_0.8−0.1
3300012145 Deep_Lake_2013_December_13_3−0.8
3300012121 Deep_Lake_2013_December_13_20−3
3300011181 Deep_Lake_2008_December_5_3−0.8
3300012262 Deep_Lake_2008_December_5_20−3
3300012027 Deep_Lake_2006_December_23_m0.1
3300012127 Deep_Lake_2006_December_23_m0.1
3300005929 Deep_Lake_2006_December_23_0.8−0.1
3300005930 Deep_Lake_2006_December_23_3−0.8
3300005928 Deep_Lake_2006_December_23_20−3
0e+00
1e+06
2e+06
Reference genome (bp)
0%
50%
100%
Percentage of genome covered
0%
20%
40%
Percentage of mapped reads

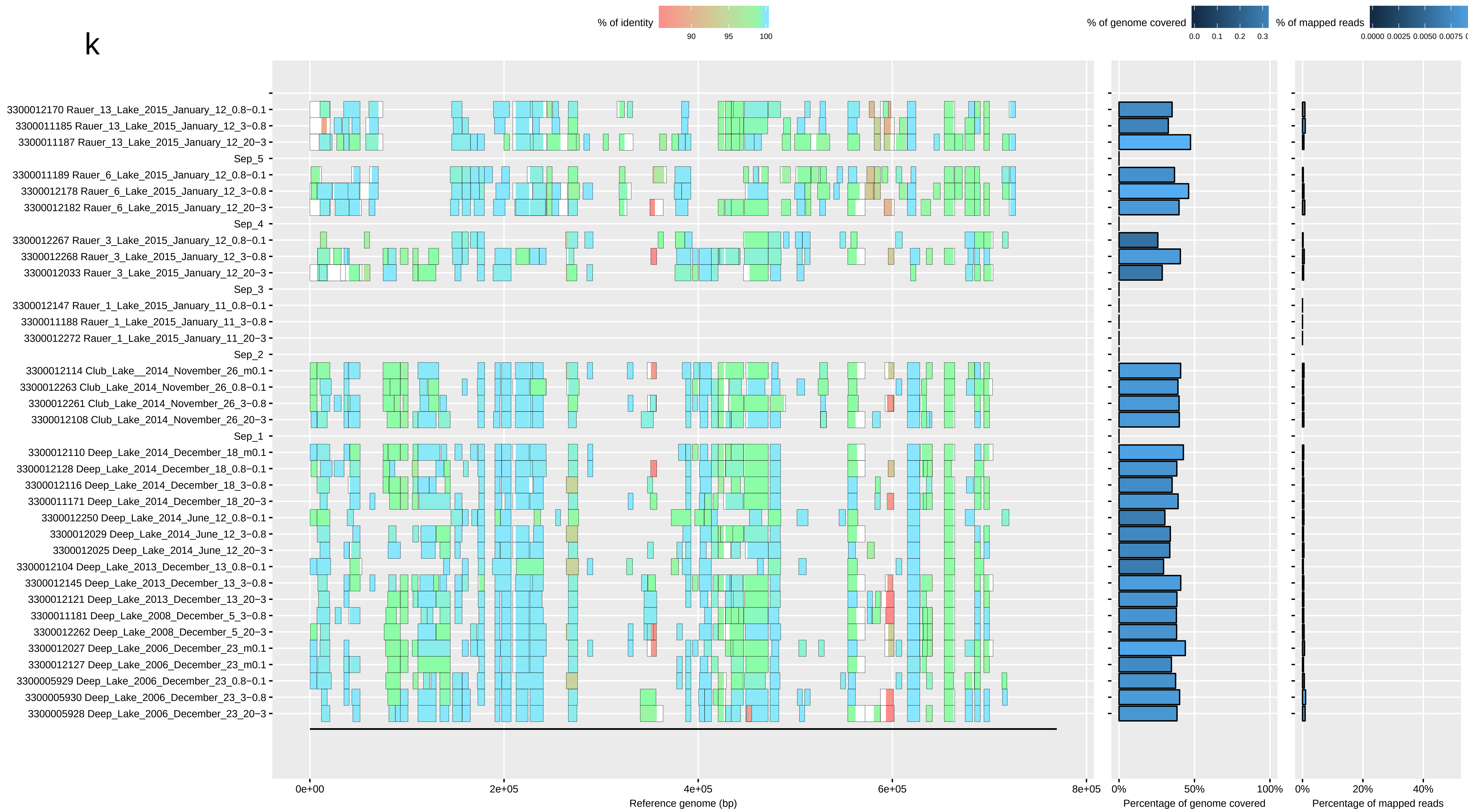
k
% of identity
90
95
100
% of genome covered
0.0
0.1
0.2
0.3
% of mapped reads
0.0000
0.0025
0.0050
0.0075
3300012170 Rauer_13_Lake_2015_January_12_0.8−0.1
3300011185 Rauer_13_Lake_2015_January_12_3−0.8
3300011187 Rauer_13_Lake_2015_January_12_20−3
Sep_5
3300011189 Rauer_6_Lake_2015_January_12_0.8−0.1
3300012178 Rauer_6_Lake_2015_January_12_3−0.8
3300012182 Rauer_6_Lake_2015_January_12_20−3
Sep_4
3300012267 Rauer_3_Lake_2015_January_12_0.8−0.1
3300012268 Rauer_3_Lake_2015_January_12_3−0.8
3300012033 Rauer_3_Lake_2015_January_12_20−3
Sep_3
3300012147 Rauer_1_Lake_2015_January_11_0.8−0.1
3300011188 Rauer_1_Lake_2015_January_11_3−0.8
3300012272 Rauer_1_Lake_2015_January_11_20−3
Sep_2
3300012114 Club_Lake__2014_November_26_m0.1
3300012263 Club_Lake_2014_November_26_0.8−0.1
3300012261 Club_Lake_2014_November_26_3−0.8
3300012108 Club_Lake_2014_November_26_20−3
Sep_1
3300012110 Deep_Lake_2014_December_18_m0.1
3300012128 Deep_Lake_2014_December_18_0.8−0.1
3300012116 Deep_Lake_2014_December_18_3−0.8
3300011171 Deep_Lake_2014_December_18_20−3
3300012250 Deep_Lake_2014_June_12_0.8−0.1
3300012029 Deep_Lake_2014_June_12_3−0.8
3300012025 Deep_Lake_2014_June_12_20−3
3300012104 Deep_Lake_2013_December_13_0.8−0.1
3300012145 Deep_Lake_2013_December_13_3−0.8
3300012121 Deep_Lake_2013_December_13_20−3
3300011181 Deep_Lake_2008_December_5_3−0.8
3300012262 Deep_Lake_2008_December_5_20−3
3300012027 Deep_Lake_2006_December_23_m0.1
3300012127 Deep_Lake_2006_December_23_m0.1
3300005929 Deep_Lake_2006_December_23_0.8−0.1
3300005930 Deep_Lake_2006_December_23_3−0.8
3300005928 Deep_Lake_2006_December_23_20−3
0e+00
2e+05
4e+05
6e+05
8e+05
Reference genome (bp)
0%
50%
100%
Percentage of genome covered
0%
20%
40%
Percentage of mapped reads

Figure S7 Contigs assembled from metagenomes mapped to replicons. DL1 primary (**a**) and secondary (**b**) replicons; *Hht. litchfieldiae* tADL single replicon (**c**); DL31 primary (**d**) and secondary (**e**, **f**) replicons; *Hrr. lacusprofundi* ACAM34 primary (**g**) and secondary (**h**, **i**) replicons; *Hrr. lacusprofundi* R1S1 primary (**j**) and secondary (**k**) replicons. Note: the smallest replicon of DL31 (~24 kb) did not recruit any contigs (**f**); contigs for the R1S1 secondary replicons were concatenated (**k**).

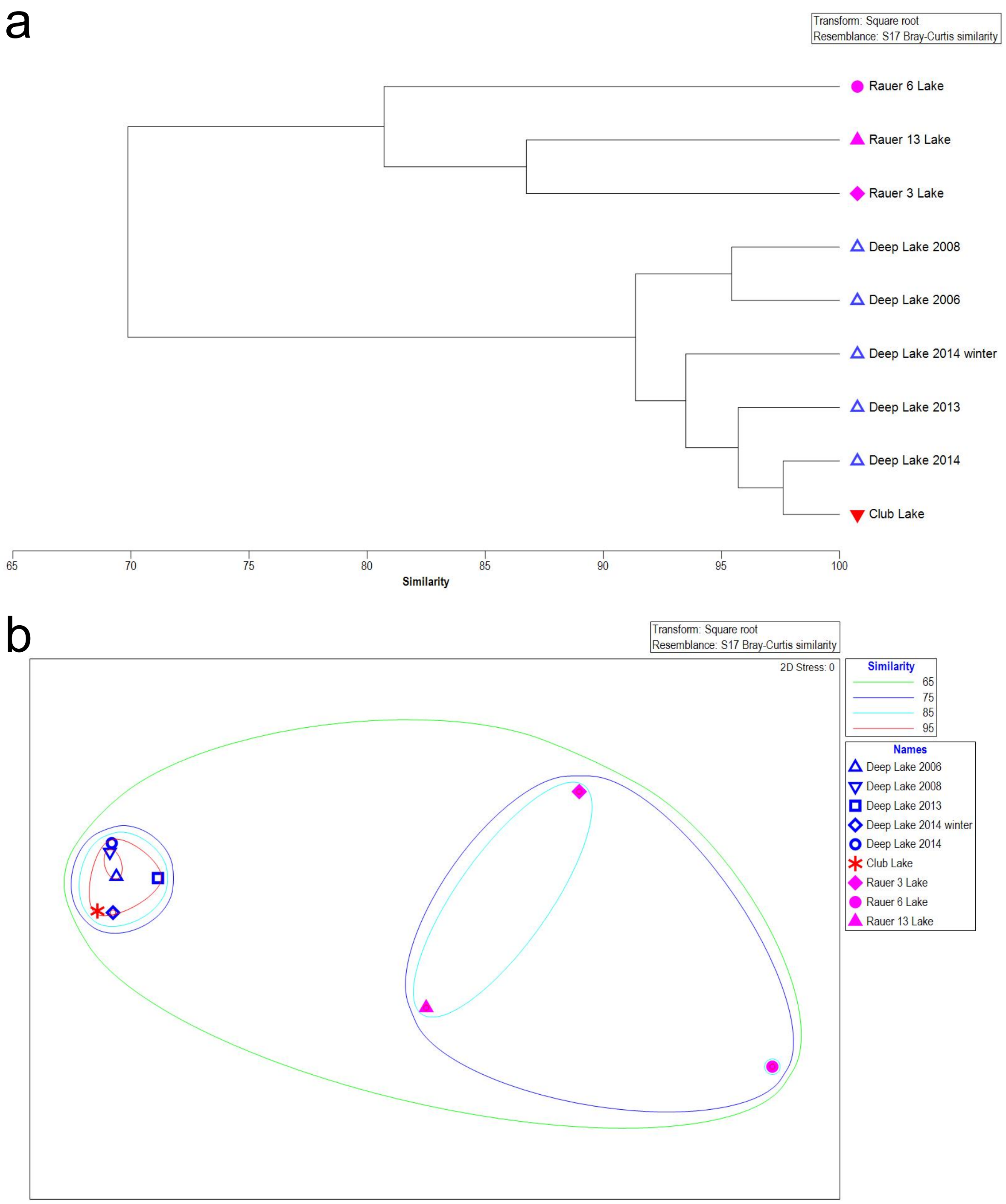

Figure S8 Clustering and scaling of samples. **a** The dendrogram depicts the clustering of the relative species abundances of taxa in five samples from Deep Lake, one from Club Lake, and one sample each from lakes in the Rauer Islands (Rauer 3, 6 and 13). Abundances were obtained from the coverages of the metagenome contigs assigned to species level, and relative abundances calculated as percentages of the total species abundance for each sample (Additional file 2: Table S12). The data were square root transformed prior to UPGMA clustering of the samples. The x-axis denotes Bray-Curtis similarity. **b** Two-dimensional scaling of the samples from (**a**) based on Bray-Curtis similarity of the square root transformed data. The UPGMA cluster was overlayed on the nMDS plot, with similarity shown at 65, 75, 85, and 95%.

Table S1 Description of the Rauer Islands and Vestfold Hills hypersaline lakes sampled in this study.

[1] **Sampling date**	[2] **Water volume filtered (L)**	[3] **Sampling coordinates (degrees Lat Long)**	[4] **Unofficial names**	[5] **New unofficial names**	[5] **Antarctic Gazetter name**	[4] **Depth max (m)**	[4] **Cond. (ms cm^{-1})**	[6] **Cond. (ms cm^{-1})**	[6] **Density (g 100 ml^{-1})**	[6] **Temp. (°C)**
**Rauer Islands lakes**										
11 Jan 2015 summer	50	-68.8081667 77.8550500	Rauer 1 Lake			7	129	178	112.3	6
11 Jan 2015 summer	50	-68.8146667 77.8273333	Rauer 3 Lake		Cavities Lake	2.5	115	164	111.5	11
11 Jan 2015 summer	50	-68.8878333 77.8395000	Rauer 6 Lake			1.5	130	119	107.6	9
11 Jan 2015 summer	50	-68.8895000 77.8448333		Rauer 13 Lake				180	113.7	9
**Vestfold Hills lakes**										
26 Nov 2014 'summer'	125 (265)	-68.5417333 78.2467667			Club Lake	nd		190	118.6	nd
23 Dec 2006 summer	55 (90)	-68.56276003 78.18827			Deep Lake	36		nd	nd	nd
5 Dec 2008 summer	50	-68.5573389 78.1868417			Deep Lake	36		nd	nd	nd
13 Dec 2013 summer	50	-68.5558667 78.1913917			Deep Lake	36		205	117.0	4
12 June 2014 winter	70	-68.5558667 78.1913917			Deep Lake	36		176	nd	nd
18 Dec 2014 summer	100 (175)	-68.56276003 78.18827			Deep Lake	36		192	117.1	2

See Additional file 1 for a description (with photographs) of the 2013-2015 lake sampling expedition.

[1] In addition to precise dates of sampling, seasons are shown. Club Lake was sampled 26 Nov which is by definition of the austral seasons, the end of spring (shown as 'summer') – at this latitude, this date equates to 24 h sunlight.

[2] Volume of water from the lakes used for capturing biomass by sequential size filtration. Surface samples were taken, although note specific details for Rauer Island lakes in Additional file 1. Values in parentheses are the volumes of water concentrated by tangential flow filtration.

[3] Photographs of lakes and their satellite map locations are shown in Figure S1. A full description of the sampling sites including photos is provided in Additional file 1.

[4] For Rauer Islands lakes, data from Hodgson *et al.,* 2001 [5]. Lake names, depth and conductivity from this study are shown.

[5] Unnamed Rauer Islands lakes were provided an unofficial name by utilizing the naming system of Hodgson *et al.,* 2001 [5]. The unofficial names (Rauer 'number' Lake) are used exclusively in this study as only one Rauer Island lake has an official name listed in the Australian Antarctic Gazetteer (https://data.aad.gov.au/aadc/gaz/).

[6] Conductivity and temperature of the water at the time of sampling are shown. As an alternative measure of salinity, density is also shown. Density was recorded as the weight of 100 mL of water weighed in a volumetric flask using a fine (four place) balance at room temperature (20°C) standardized against 100 mL of deionised water. Temperature was measured with a glass thermometer; nd, not determined.

Table S2 Antarctic lake metagenomes used in this study.

Lake name	Year	Month - Day	Size fraction	IMG genome ID	Gold Analysis Project ID	Assembled metagenome size (bp)
Deep Lake	2006	December 23rd	20 - 3 µm	3300005928	Ga0075105	127444550
Deep Lake	2006	December 23rd	3 - 0.8 µm	3300005930	Ga0075106	188685715
Deep Lake	2006	December 23rd	0.8 - 0.1 µm	3300005929	Ga0075107	119306653
Deep Lake	2006	December 23rd	< 0.1 µm	3300012127	Ga0136608	110118418
Deep Lake	2006	December 23rd	< 0.1 µm	3300012027	Ga0136609	79462683
Deep Lake	2008	December 5th	20 - 3 µm	3300012262	Ga0136606	86277388
Deep Lake	2008	December 5th	3 - 0.8 µm	3300011181	Ga0136607	101045660
Deep Lake	2013	December 13th	20 - 3 µm	3300012121	Ga0136565	100145597
Deep Lake	2013	December 13th	3 - 0.8 µm	3300012145	Ga0136566	161744775
Deep Lake	2013	December 13th	0.8 - 0.1 µm	3300012104	Ga0136567	67783456
Deep Lake	2014	June 12th	20 - 3 µm	3300012025	Ga0136571	73641348
Deep Lake	2014	June 12th	3 - 0.8 µm	3300012029	Ga0136572	100880304
Deep Lake	2014	June 12th	0.8 - 0.1 µm	3300012250	Ga0136573	60323580
Deep Lake	2014	December 18th	20 - 3 µm	3300011171	Ga0136580	70990421
Deep Lake	2014	December 18th	3 - 0.8 µm	3300012116	Ga0136581	88575811
Deep Lake	2014	December 18th	0.8 - 0.1 µm	3300012128	Ga0136582	107097935
Deep Lake	2014	December 18th	< 0.1 µm	3300012110	Ga0136593	102592878
Club Lake	2014	November 26th	20 - 3 µm	3300012108	Ga0136562	83416154
Club Lake	2014	November 26th	3 - 0.8 µm	3300012261	Ga0136563	71167471
Club Lake	2014	November 26th	0.8 - 0.1 µm	3300012263	Ga0136564	86361097
Club Lake	2014	November 26th	< 0.1 µm	3300012114	Ga0136595	84310686
Rauer 1 Lake	2015	January 11th	20 - 3 µm	3300012272	Ga0136586	317629528
Rauer 1 Lake	2015	January 11th	3 - 0.8 µm	3300011188	Ga0136587	339573373
Rauer 1 Lake	2015	January 11th	0.8 - 0.1 µm	3300012147	Ga0136588	153987876
Rauer 3 Lake	2015	January 12th	20 - 3 µm	3300012033	Ga0136589	282086351
Rauer 3 Lake	2015	January 12th	3 - 0.8 µm	3300012268	Ga0136590	171063290
Rauer 3 Lake	2015	January 12th	0.8 - 0.1 µm	3300012267	Ga0136591	157623777
Rauer 6 Lake	2015	January 12th	20 - 3 µm	3300012182	Ga0136556	338478872
Rauer 6 Lake	2015	January 12th	3 - 0.8 µm	3300012178	Ga0136557	319376726
Rauer 6 Lake	2015	January 12th	0.8 - 0.1 µm	3300011189	Ga0136558	329351461
Rauer 13 Lake	2015	January 12th	20 - 3 µm	3300011187	Ga0136596	244559929
Rauer 13 Lake	2015	January 12th	3 - 0.8 µm	3300011185	Ga0136597	201429522
Rauer 13 Lake	2015	January 12th	0.8 - 0.1 µm	3300012170	Ga0136598	251362283

Table S3 Unique transposase genes of *Hrr. lacusprofundi* strains ACAM34 and R1S1.

ACAM34			R1S1			
**Position**	**Length in bp**	**Locus tags**	**Position**	**Length in bp**	**Locus tags**	**Comments**
-	-	-	220497…221663	1167	Ga0123509_160215	
53104…54566	1463	Hlac_0054	-	-	-	
-	-	-	726583..727833	1251	Ga0123509_160717	
452870…454709	1840	Hlac_0436/ 0437	-	-	-	
597160…598692	1533	Hlac_0580	-	-	-	Within a region of low similarity (Table S4)
-	-	-	914035…915183	1149	Ga0123509_160913	
919624…920622	999	Hlac_0921	-	-	-	
1015778…1017239	1462	Hlac_1024	-	-	-	
-	-	-	1328455…1329885	1431	Ga0123509_1601346	This transposase gene lies within the ACAM34 gene Hlac_1071 (hypothetical with five-bladed β-propellor domain found in some glycosyl hydrolases); within a region of low similarity (Table S4)
1137543…1139396	1854	Hlac_1128	-	-	-	
1222965…1223963	999	Hlac_1212	-	-	-	
-	-	-	1707365…1708804	1440	Ga0123509_1601717	
1501533…1502171	639	Hlac_1494	-	-	-	
1525929…1526567	639	Hlac_1512	-	-	-	
-	-	-	1848367…1849545	1179	Ga0123509_1601858	
1625006…1626467	1462	Hlac_1606	-	-	-	
-	-	-	1918636…1919808	1173	Ga0123509_1601929	
1771722…1772970	1249	Hlac_1756	-	-	-	
1772965…1774372	1408	Hlac_1757	-	-	-	
1775301…1776702	1402	Hlac_1759	-	-	-	This transposase gene lies within the R1S1 gene Ga0123509_1602045 which is annotated as putative replication initiator protein (rep)

1847940…1849401	1462	Hlac_1840	-	-	-	
			2257564…2258808	1245	Ga0123509_1602307	Within a region of low similarity (Table S4)
2082149…2083550	1402	Hlac_2092	-	-	-	
2238850…2240187	1338	Hlac_2250	-	-	-	
-	-	-	2581257…2582977	1721	Ga0123509_1602633/ 1602634	This transposase gene lies within the ACAM34 gene Hlac_2333 which is annotated as an uncharacterised membrane protein.

All transposase genes which are not present at the equivalent genomic position in the other strain are listed.

Table S4 Unique protein-coding genes on *Hrr. lacusprofundi* ACAM 34 and R1S1 primary replicons.

ACAM34			R1S1			
**Position**	**Length in bp**	**Locus tags**	**Position**	**Length in bp**	**Locus tags**	**Annotation and comments**
-	-	-	85713…86333	620	Ga0123509_16091	Two consecutive archaellin genes (*flaB*) in R1S1 where ACAM34 has only one archaellin gene
-	-	-	782723…783296	574	Ga0123509_160778	Putative cell surface protein, absent in ACAM34
605333…606991	1659	Hlac_0587	-	-	-	α-amylase, absent in R1S1
750801…779997	29197	Hlac_0736 to 0775	1038242…1039459	1217	Ga0123509_1601043	Provirus Hlac-Pro1 in ACAM34. R1S1 contains a gene encoding for a putative cell surface protein at the equivalent location
1057147…1057488	342	Hlac_1060	-	-	-	Protein with ArsR-like helix-turn-helix (DNA-binding) domain, absent in R1S1; within a region of low similarity (Table S6)
1081868…1082583	716	Hlac_1081/ 1082	-	-	-	Putative toxin-antitoxin (TA) system, absent in R1S1
1972868…1973351	484	Hlac_1975 (partly)	-	-	-	Part of hypothetical protein and intergenic region, absent in R1S1

Eight protein-coding genes in seven distinct genomic loci on the primary replicons were identified as unique to either ACAM34 or R1S1.

Table S5 Unique sequence duplications and non-coding RNAs on the R1S1 primary replicon.

Position	Length in bp	Locus tags	Comments and annotation
75437…76027 and 76016…76606	591	Ga0123509_16078 to 16081	Duplication of 591 nt in R1S1; affected ORFs are annotated as ERCC domain protein and hypothetical proteins
403081…403459	379	Ga0123509_160398 to 160401	Four identical tRNA-Asp copies side by side in R1S1; only two copies in ACAM34
1169239…1169367	128	Ga0123509_1601183	HgcC family ncRNA, absent in ACAM34
2581257…2582977	1721	Ga0123509_1602632	HgcC family ncRNA. Adjacent to the ncRNA are two transposase genes which are absent in ACAM34 (Ga0123509_1602633/ 1602634; Table S3)

These types of unique sequences were not identified on the ACAM34 primary replicon.

Table S6 Regions with low sequence similarity between ACAM34 and R1S1 primary replicons.

	ACAM34		R1S1			
#	Position	Locus tags	Position	Locus tags	ID	Comments and annotation of encoded genes
1	114519 …123903	Hlac_0109 to 0118	403285 …412681	Ga0123509 _160402 to _1060413	95%	Acetoin utilization deacetylase AcuC; archaeal histone-like protein; replication factor A1; tRNA-Arg; sodium/proton antiporter, CPA1 family (TC2.A.36); NAD+ dependent glucose-6-phosphate dehydrogenase; putative cell surface protein (cytoplasmic, transmembrane and non-cytoplasmic domains); hypothetical (DUF309 domain); hypothetical (uncharacterized protein family SepF-related domain); hypothetical
2	426616 …428965	Hlac_0409 (partly) to 0411	715497 …717777	Ga0123509 _160708 (partly) to 160710	93%	Hypothetical (WD40/YVTN repeat-like-containing domain, oligoxyloglucan reducing end-specific cellobiohydrolase superfamily); integral membrane protein-like protein; putative transcription regulator, CopG/Arc/MetJ family
	428966… 432296	Hlac_0412	717778 …720739	Ga0123509 _160711	54%	S-layer glycoprotein
3	596866 …597159	Hlac_0579	885886 …886179	Ga0123509 _160884	93%	Hypothetical (multiple transmembrane and non-cytoplasmic domains)
	597160 …598692	Hlac_0580	-	-	-	Transposase gene, absent in R1S1
	598833 …605292	Hlac_0581 to 0586	886275 …892719	Ga0123509 _160885 to 160891	89%	Dipeptidyl aminopeptidase/acylaminoacyl peptidase; glycosyltransferase; halocyanin domain-containing protein; phosphoribosyltransferase; MoxR-like ATPase; tRNA_Ser; hypothetical

4	1045360 …1056892	Hlac_1051 to 1059	1302658 …1314181	Ga0123509 _1601325 to 1601334	93%	Xaa-Pro aminopeptidase; phosphoglycerate dehydrogenase; amidohydrolase; 4-aminobutyrate aminotransferase; choline/carnitine/betaine transport; amidohydrolase; alcohol dehydrogenase zinc-binding domain protein; capsule biosynthesis protein; hypothetical
	1057147 …1057488	Hlac_1060	-	-	-	Hypothetical protein, absent in R1S1;
	1057665 …1071621	Hlac_1061 to 1071	1314574 …1328460	Ga0123509 _1601335 to 1601345	95%	Putative membrane protein; oligosaccharyltransferase STT3 subunit AglB; glycosyl transferase family 2; glycosyl transferase group 1; sulfatase; glycosyl transferase group 1; hypothetical (alkaline-phosphatase-like core domain); polysaccharide biosynthesis protein; formyl transferase; hypothetical with five-bladed β-propellor domain found in some glycosyl hydrolases
	-	-	1328455 …1329885	Ga0123509 _1601346	-	Transposase gene, absent in ACAM34
	1071622 …1081793	Hlac_1072 to 1080	1329886 …1340080	Ga0123509 _1601347 to 1601355	95%	NAD-dependent epimerase/dehydratase; NAD-dependent epimerase/dehydratase; UDP-sulfoquinovose synthase; transcriptional regulator TrmB; hypothetical; ORC complex protein Cdc6/Orc1; glutamine--fructose-6-phosphate transanimase; nucleotidyl transferase; hypothetical
5	1999910 …2007189	Hlac_2006 to 2013	2249787 …2257210	Ga0123509 _1602299 to 1602306	91%	Membrane protein involved in the export of O-antigen and teichoic acid; transcriptional regulator, TetR family; succinylglutamate desuccinylase; arginine decarboxylase; proteasome-activating nucleotidase; response regulator receiver protein; hypothetical
	-	-	2257564 …2258808	Ga0123509 _1602307	-	Transposase gene, absent in ACAM34
	2007190 …2008357	Hlac_2014	2258824 …2259992	Ga0123509 _1602308	95%	Peptidase M29 aminopeptidase I

Annotations are from IMG.

Table S7 New HIRs identified in R1S1.

HIR length (bp)	ID (%)	R1S1 contig	start	end	DL species/replicon	start	end
13970	99.87	Ga0123509_141	127	14096	DL1/HalDL1_Contig37 (secondary replicon)	278097	264129
9603	100	Ga0123509_133	1	9603	DL31/DL31_replicon 2 (secondary replicon)	101664	111266
9388	99.93	Ga0123509_155	22279	31666	DL1/HalDL1_Contig37 (secondary replicon)	16880	7493
6021	100	Ga0123509_135	58	6078	DL31/DL31_replicon 2 (secondary replicon)	363470	357450
4800	99.98	Ga0123509_151	21705	26504	DL31/DL31_replicon 2 (secondary replicon)	451899	456698
4386	100	Ga0123509_137	3750	8135	*Hht. litchfieldiae* tADL/Hlit_replicon (single replicon)	1224426	1220041
4302	100	Ga0123509_154	263	4564	DL1/HalDL1_Contig37 (secondary replicon)	97783	102084
4225	99.95	Ga0123509_135	6073	10297	DL31/DL31_replicon 2 (secondary replicon)	297162	292938
4064	99.98	Ga0123509_137	8125	12188	*Hht. litchfieldiae* tADL/Hlit_replicon (single replicon)	1504145	1508208
3694	*99.89*	*Ga0123509_141*	*1501*	*5194*	*Hht. litchfieldiae tADL/Hlit_replicon (single replicon)*	*1483561*	*1479868*
3469	99.94	Ga0123509_145	10141	13609	DL1/HalDL1_Contig37 (secondary replicon)	250326	246858
2330	100	Ga0123509_120	1	2330	DL31/DL31_replicon 2 (secondary replicon)	679626	677297

HIRs shared between the R1S1 secondary replicon contigs and *Hht. litchfieldiae* tADL, DL31 or DL1. The region highlighted in italics is part of the 14 kb HIR shared between R1S1 and DL1. Contig and replicon names are from IMG.

Table S8 HIR conserved between *Hrr. lacusprofundi* R1S1 and DL1 that encodes a type I-B CRISPR system.

	Hrr. lacusprofundi **R1S1**	**DL1**	**ID**
**Cas6**	Ga0123509_15595	HalDL1_3060	100%
**Cas8b**	Ga0123509_15594	HalDL1_3061	100%
**Cas7**	Ga0123509_15593	HalDL1_3062	100%
**Cas5h**	Ga0123509_15592	HalDL1_3063	100%
**Cas3**	Ga0123509_15591	HalDL1_3064	99.8%
**Cas4**	Ga0123509_15590	HalDL1_3065	99.5%
**Cas1**	Ga0123509_15589	HalDL1_3066	100%
**Cas2**	Ga0123509_15588	HalDL1_3067	100%
**Repeat**	ACTTCAACCCCACGAGGGTACGTCTGAAAC	ACTTCAACCCCACGAGGGTACGTCTGAAAC	100%

The table lists the Cas protein names, the corresponding IMG locus tags for R1S1 and DL1, and the CRISPR repeat sequences. ID refers to amino acid identity for Cas proteins and nucleotide identity for spacer sequences.

Table S9 R1S1 CRISPR spacer matching Hlac-Pro1 in ACAM34.

	R1S1		ACAM34	
	**Coordinates**	**Annotation**	**Coordinates**	**Annotation**
CTCAACATCCCCGCAGGGTGACCTGGCCTTCCGGA	20539…20573; secondary replicon contig Ga0123509_155	CRISPR spacer	756383…756349; primary replicon	Part of Hlac-Pro1: intergenic region between Hlac_R0018 (tRNA_Phe) and Hlac_0748 (hypothetical)

Table S10 Presence of 16S rRNA gene sequences for known Antarctic haloarchaeal species in Antarctic hypersaline lakes.

	Hrr. lacusprofundi	***Hht. litchfieldiae***	**DL31**	**DL1**
Deep Lake	yes	yes	yes	yes
Club Lake	yes	yes	yes	yes
Rauer 1 Lake	no	no	no	no
Rauer 3 Lake	yes	yes	yes	yes
Rauer 6 Lake	yes	yes	yes	no
Rauer 13 Lake	yes	yes	yes	yes

Presence (yes) or absence (no) of 16S rRNA genes sequences in the metagenomes of the six lakes studied, determined using BLAST.

Table S11 Relative abundance of *Hrr. lacusprofundi* ACAM34, *Hht. litchfieldiae* tADL, DL31 and DL1 in Antarctic lake metagenomes.

	***Hrr. lacusprofundi* ACAM34**	***Hht. litchfieldiae* tADL**	**DL31**	**DL1**
Deep Lake 2014	9 (227)	63 (1555)	27 (662)	0.5 (13)
Deep Lake 2008	10 (108)	53 (570)	36 (388)	0.8 (9)
Deep Lake 2013	10 (167)	67 (1118)	22 (371)	0.6 (10)
Club Lake	10 (241)	62 (1426)	27 (632)	0.8 (19)
Deep Lake 2006	11 (527)	59 (2760)	29 (1343)	0.6 (28)
Deep Lake 2014 winter	13 (191)	63 (920)	24 (346)	0.7 (11)
Rauer 3 Lake	22 (56)	53 (135)	22 (56)	4 (10)
Rauer 13 Lake	43 (251)	32 (185)	25 (146)	0.5 (3)
Rauer 6 Lake	47 (107)	12 (28)	40 (91)	0.9 (2)

Relative abundance is shown as a percentage of the median coverage values (shown in parentheses) of metagenome reads mapped onto the primary replicons.

Table S12 Relative abundance of lake taxa assessed from read coverage and taxonomic assignment of contigs assembled from metagenome data.

	Halohasta litchfieldiae	***Halorubrum lacusprofundi***	**DL31**	**DL1**	**Other archaea**	**Bacteria**	**Eucarya**	**Viruses**
Deep Lake 2006	45	11	27	0.5	9	3	0.5	4
Deep Lake 2008	36	14	29	1	11	3	0.5	4
Deep Lake 2013	45	13	13	0.7	17	3	0.5	7
Deep Lake 2014 winter	38	10	14	0.7	24	3	0.1	10
Deep Lake 2014	44	12	20	0.7	15	2	0.4	6
Club Lake 2014	44	11	18	0.7	19	2	0.2	6
Rauer 1 Lake 2015	0	0	0	0	0	66	10	24
Rauer 3 Lake 2015	19	12	8	3	5	42	8	4
Rauer 6 Lake 2015	3	3	9	0.3	3	66	5	10
Rauer 13 Lake 2015	16	9	13	0.6	14	35	3	10

Abundances were obtained from the coverages of the metagenome contigs assigned to species level, and relative abundances shown as percentages of the total species abundance for each sample.

Table S13 Genome coverage and percent identity for contigs mapped to replicons of *Hrr. lacusprofundi* R1S1 and ACAM34, *Hht. litchfieldiae* tADL, DL31 and DL1.

		% of genome covered by contigs (hits of ≥5kb)	Average percentage of identity of hits
Hrr. lacusprofundi **ACAM34**	**Primary_NC_012029**	**82**	**99.4**
	Secondary_NC_012028	27	99.7
	Secondary_NC_012030	33	99.8
Hrr. lacusprofundi **R1S1**	**Primary_Ga0123509_160**	**84**	**99.3**
	Secondary_contigs	48	99.6
Hht. litchfieldiae **tADL**	**Primary_Contig32**	**44**	**97.9**
**DL31**	**Primary_Contig115**	**56**	**99.6**
	Secondary_Contig113	0	-
	Secondary_Contig114	32	99.8
**DL1**	**Primary_HalDL1_Contig38**	**2.7**	**98.1**
	Secondary_HalDL1_Contig37	18	97.8

References

1. McWilliam H, Li W, Uludag M, Squizzato S, Park YM, Buso N et al. Analysis tool web services from the EMBL-EBI. Nucleic Acids Res. 2013;41:W597-600.
2. DeMaere MZ, Williams TJ, Allen MA, Brown MV, Gibson JA, Rich J et al. High level of intergenera gene exchange shapes the evolution of haloarchaea in an isolated Antarctic lake. Proc Natl Acad Sci U S A. 2013;110:16939–16944.
3. Hodgson DA, Vyverman W, Sabbe K. Limnology and biology of saline lakes in the Rauer Islands, eastern Antarctica. Antarctic Sci. 2001;13:255-270.
